# Prospective longitudinal evaluation of treatment-related toxicity and health-related quality of life during the first year of treatment for pediatric acute lymphoblastic leukemia

**DOI:** 10.1186/s12885-022-10072-x

**Published:** 2022-09-15

**Authors:** Clarissa E. Schilstra, Karen McCleary, Joanna E. Fardell, Mark W. Donoghoe, Emma McCormack, Rishi S. Kotecha, Richard De Abreu Lourenco, Shanti Ramachandran, Ruelleyn Cockcroft, Rachel Conyers, Siobhan Cross, Luciano Dalla-Pozza, Peter Downie, Tamas Revesz, Michael Osborn, Frank Alvaro, Claire E. Wakefield, Glenn M. Marshall, Marion K. Mateos, Toby N. Trahair

**Affiliations:** 1 Discipline of Paediatrics and Child Health, School of Clinical Medicine, UNSW Medicine & Health, UNSW Sydney, Kensington, NSW Australia; 2 Behavioural Sciences Unit, Kids Cancer Centre, Sydney Children’s Hospitala, High Street, Randwick, NSW 2031 Australia; 3 Kids Cancer Centre, Sydney Children’s Hospital, High Street, Randwick, NSW 2031 Australia; 4 Stats Central, Mark Wainwright Analytical Centre, UNSW Sydney, Kensington, NSW Australia; 5 Department of Clinical Haematology, Oncology, Blood and Marrow Transplantation, Perth Children’s Hospital, Nedlands, WA Australia; 6 Telethon Kids Cancer Centre, Telethon Kids Institute, University of Western Australia, Perth, WA Australia; 7 Curtin Medical School, Curtin University, Perth, WA Australia; 8 Centre for Health Economics Research and Evaluation, University of Technology Sydney, Sydney, NSW Australia; 9 School of Paediatrics and Child Health, University of Western Australia, Perth, WA Australia; 10 Starship Children’s Hospital, Auckland, New Zealand; 11 Royal Children’s Hospital, Melbourne, VIC Australia; 12 Department of Paediatrics, Melbourne University, Melbourne, VIC Australia; 13 Murdoch Children’s Research Insitute, Parkville, VIC Australia; 14 Children’s Haematology Oncology Centre, Christchurch Hospital, Christchurch, New Zealand; 15 Cancer Centre for Children, the Children’s Hospital at Westmead, Westmead, NSW Australia; 16 Monash Children’s Hospital, Clayton, VIC Australia; 17 Women’s and Children’s Hospital, Adelaide, SA Australia; 18 University of Adelaide, Adelaide, SA Australia; 19 John Hunter Children’s Hospital, New Lambton Heights, NSW Australia; 20 Children’s Cancer Institute, Lowy Cancer Research Centre, UNSW Sydney, Kensington, NSW Australia

**Keywords:** ALL, Health related quality of life, Treatment related toxicity, Quality of life, Psychosocial, Child, Registries

## Abstract

**Background:**

Pediatric acute lymphoblastic leukemia (ALL) therapy is accompanied by treatment-related toxicities (TRTs) and impaired quality of life. In Australia and New Zealand, children with ALL are treated with either Children’s Oncology Group (COG) or international Berlin-Frankfurt-Munster (iBFM) Study Group-based therapy. We conducted a prospective registry study to document symptomatic TRTs (venous thrombosis, neurotoxicity, pancreatitis and bone toxicity), compare TRT outcomes to retrospective TRT data, and measure the impact of TRTs on children’s general and cancer-related health-related quality of life (HRQoL) and parents’ emotional well-being.

**Methods:**

Parents of children with newly diagnosed ALL were invited to participate in the ASSET (Acute Lymphoblastic Leukaemia Subtypes and Side Effects from Treatment) study and a prospective, longitudinal HRQoL study. TRTs were reported prospectively and families completed questionnaires for general (Healthy Utility Index Mark 3) and cancer specific (Pediatric Quality of Life Inventory (PedsQL)-Cancer Module) health related quality of life as well the Emotion Thermometer to assess emotional well-being.

**Results:**

Beginning in 2016, 260 pediatric patients with ALL were enrolled on the TRT registry with a median age at diagnosis of 59 months (range 1–213 months), 144 males (55.4%), majority with Pre-B cell immunophenotype, *n* = 226 (86.9%), 173 patients (66.5%) treated according to COG platform with relatively equal distribution across risk classification sub-groups. From 2018, 79 families participated in the HRQoL study through the first year of treatment. There were 74 TRT recorded, reflecting a 28.5% risk of developing a TRT. Individual TRT incidence was consistent with previous studies, being 7.7% for symptomatic VTE, 11.9% neurotoxicity, 5.4% bone toxicity and 5.0% pancreatitis. Children’s HRQoL was significantly lower than population norms throughout the first year of treatment. An improvement in general HRQoL, measured by the HUI3, contrasted with the lack of improvement in cancer-related HRQoL measured by the PedsQL Cancer Module over the first 12 months. There were no persisting differences in the HRQoL impact of COG compared to iBFM therapy.

**Conclusions:**

It is feasible to prospectively monitor TRT incidence and longitudinal HRQoL impacts during ALL therapy. Early phases of ALL therapy, regardless of treatment platform, result in prolonged reductions in cancer-related HRQoL.

**Supplementary Information:**

The online version contains supplementary material available at 10.1186/s12885-022-10072-x.

## Introduction

Survival for pediatric acute lymphoblastic leukaemia (ALL) has made excellent gains with five-year overall survival ≥90% [1, 2]. ALL treatment is prolonged, lasting up to 3 years, with many treatment-related toxicities (TRTs) [1, 2]. Nearly 40% of ALL patients experience ≥1 TRT. The incidence of venous thromboembolism being ≈5% [3–6], neurotoxicity (central and/or peripheral) 3–28% [7–10], osteonecrosis 6–17%, fractures 8–28% [11] and pancreatitis 5–10% [12–15]. Some TRTs are life-threatening and many contribute to late effects that negatively impact health related quality of life (HRQoL) [14, 16–20].

While advances in ALL risk classification and treatment have occurred [2], TRT research is an emerging discipline [14, 18]. Survival achieved by different ALL consortia are equivalent [2], but with limited comparative data on TRT incidence and HRQoL impacts across different protocols. Tentative evidence suggests that HRQoL is worst early in treatment, when increased risk of toxicity is more likely, with improvement over time [19–22]. Children on treatment report poorer HRQoL than those who have completed treatment [21] and compared to healthy children [23]. Few studies have taken a longitudinal approach to monitoring HRQoL in ALL [23–30]. Two studies used a cancer-related HRQoL measure, making it difficult to draw conclusions regarding treatment-specific impacts, such as pain or nausea, on HRQoL [23, 27].

In an era where it is possible to achieve excellent survival, we suggest that TRT incidence, patient and parent HRQoL, and financial cost of therapy should be considered when evaluating the overall risks and benefits of alternative treatments. In considering HRQoL and parents’ emotional wellbeing during ALL treatment, we hypothesise that it is possible that the HRQoL impact across different treatment platforms is likely equivalent during early more intensive treatment, due to the broad similarities in the early treatment. However, later on, HRQoL differences may emerge, particularly for more intensive or prolonged maintenance protocols *eg* containing steroid pulses, vincristine and intrathecal chemotherapy. Recent Dutch HRQoL data during maintenance therapy, has shown that more intensive maintenance therapy, including intermittent dexamethasone, is associated with impaired HRQoL and higher distress compared to less intensive maintenance [31].

We previously undertook a retrospective analysis of TRT in ALL, the ERASE (Evaluation of Risk of ALL Treatment-Related Side-Effects) study, a multi-center study of 1251 children (1–18 years), consecutively diagnosed with ALL or lymphoblastic lymphoma, at six Australian Centers between 1998 and 2013 [6, 10, 32]. ERASE focused on developing clinical and/or genetic risk prediction models for TRTs [6, 10, 32]. Symptomatic TRTs of interest were venous thromboembolism, neurotoxicity, bone toxicity and pancreatitis. ERASE did not involve HRQoL monitoring. The prospective ASSET (Acute Lymphoblastic Leukaemia Subtypes and Side Effects from Treatment) study was designed to validate risk prediction models from ERASE and prospectively document longitudinal HRQoL. The research goals are to prospectively identify ALL patients at increased TRT risk, develop interventions to ameliorate TRT risk, capture whole of life impacts from ALL and its treatment, and to identify the costs of managing ALL and associated TRTs.

The ASSET study provides an opportunity to compare HRQoL between different treatment protocols within a broadly uniform healthcare system. Here, we aim to confirm the capacity of the ASSET registry to prospectively capture TRTs; compare rates of TRTs with the retrospective ERASE study; confirm the feasibility to prospectively capture children’s HRQoL and parents’ emotional well-being, examine whether children’s HRQoL and parents’ emotional well-being differed significantly across COG and iBFM treatment protocols and compare ASSET HRQoL outcomes to those documented in previous literature.

## Methods

### Participants

#### ASSET treatment related toxicity study

In January 2016, the ASSET study opened to recruitment at 9 Australian and New Zealand centers, the HRQoL substudy opened to recruitment in October 2018. Inclusion criteria included: newly diagnosed ALL or mixed phenotype acute leukaemia (MPAL), age ≤ 18 years and enrolment within 90 days of starting treatment. Patients with relapsed ALL/MPAL were excluded. Patients and their parents were identified by their treating clinicians and invited to participate by a research coordinator. The majority centers (7 of 9) used COG-based therapy with iBFM-based therapy in the others 2 centers (Table 1). The study was approved by the HNE HREC (2019/ETH00693) and was conducted according to the Australian National Statement on Ethical Conduct in Human Research (2007) [33]. Informed consent was obtained from participants or their parents and/or legal guardians. Data, including clinical features, treatment, ALL outcomes and symptomatic TRT incidence and management, was collected prospectively by the local clinicians and entered into a web-based registry (WebSpirit). Targeted symptomatic TRTs including venous thromboembolism, neurotoxicity (central and peripheral), bone toxicity and pancreatitis were prospectively documented to compare to the ERASE study. Follow-up data collection including 6-monthly updates of symptomatic TRTs, clinical progress and survival including relapse, second malignancy and/or death. TRTs were prospectively identified, graded and reported by treating clinicians at each center using international definitions and the National Cancer Institute Common Toxicology Criteria for Adverse Events (NCI-CTCAE) version 4.03 [17, 34].

**Table 1:** Participating centers in the ASSET study and COG, iBFM and Interfant based ALL treatment programs

Center	ALL treatment approach & treatment programs
Sydney Children’s Hospital	iBFM
Children’s Hospital at Westmead	iBFM
John Hunter Children’s Hospital	COG
Perth Children’s Hospital	COG
Monash Children’s Hospital	COG
The Royal Children’s Hospital	COG
Women’s and Children’s Hospital	COG
Christchurch Hospital	COG
Starship Children’s Hospital	COG
COG, iBFM and Interfant based treatment programs used in Australian and New Zealand Centers	COG A5971, ANZCCSG Study VII, ANZCHOG Study 8, AIEOP-BFM ALL 2009-Study 9, BFM-95, COG AALL0031, COG AALL0232, COG AALL0331, COG ALL0434, COG AALL08P1, COG ALL0932, COG AALL1131, CCG1882, CCG1952, CCG1961, CCG1991, Interfant-99 and Interfant-06

#### ASSET health related quality of life study

**Fig. 1:**
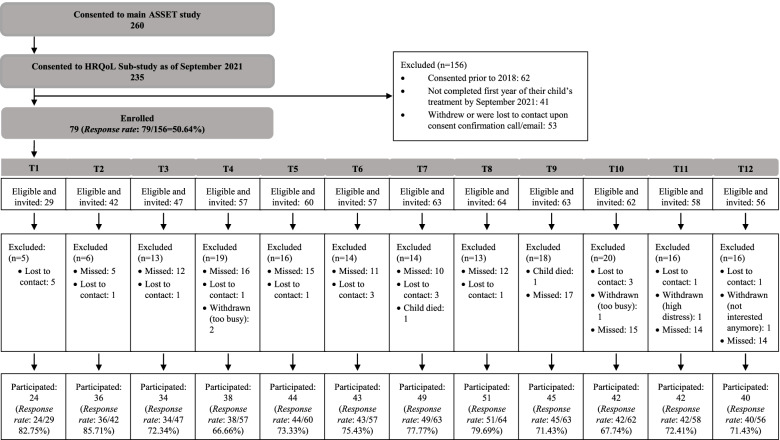
ASSET HRQoL Sub-Study Recruitment and Participation. Sevety-nine families consented and provided at least one questionnaire response across the first year of treatment. Sample size at each time point post diagnosis is shown. Response rate at each time point is calculated by considering the number of participants in the total sample that were within the specified time frame for a particular questionnaire, and who were invited and provided data at that time point. For example, at T3, 47 participants of the total 79 were within 2 weeks of 3 months post diagnosis and were invited to complete a survey. Of these 47, 34 provided data, 12 missed completing the questionnaire within the allocated time frame (2 weeks) and one was lost to contact

Patients enrolled on ASSET, except at one site (due to staffing requirements), were invited to participate in the HRQoL study. Following consent, eligible parents and children/young people were sent an invitation email to complete their first study questionnaire via Research Electronic Data Capture (REDCap) [35, 36]. The first questionnaire was sent within 100 days of the child or young person’s diagnosis and participants were given up to 14 days to complete it (Fig. 1). Due to delayed study staff recruitment, ASSET HRQoL study commenced data collection in October 2018. HRQoL was assessed monthly during the child or young person’s first year of treatment. Response rates were tracked for the first 12 questionnaires, which cover the first 12 months of treatment: T1 = 1 month post-diagnosis, T2 = 2 months post-diagnosis, etc. Participants were contacted by phone and/or email if they missed two consecutive questionnaires. A follow-up log was maintained to track reasons for missed questionnaires.

Patients’ ALL treatment was determined by the treating clinical team following local institutional practices and could include enrolment on current COG, iBFM or Interfant therapeutic clinical trials or treatment according to published trials (Table 1) [37–52].

### HRQoL measures

Demographics and clinical information were collected from the medical records and from both parents and young people through purpose-designed questions asking about the child or young person’s sex, date of birth, and time since diagnosis.

Cancer-related HRQoL was assessed using The Pediatric Quality of Life Inventory (PedsQL)-Cancer Module (child, youth, and parent proxy versions) [53]. The PedsQL-Cancer Module assesses cancer-related HRQoL and has 23 items with eight scales, scored from 0 to 4. The scales assess children, youth, and parents’ perceptions of young people’s levels of pain, nausea, procedural anxiety, treatment anxiety, worry, cognitive problems, changed physical appearance and communication. Individual item scores were transformed (0 = 100, 1 = 75, 2 = 50, 3 = 25, 4 = 0), and subscale mean scores were calculated. An overall HRQoL score was generated by calculating the mean of the subscale scores. Higher scores indicate higher HRQoL.

General HRQoL was assessed using the Healthy Utility Index Mark 3 (HUI3) [54]. The HUI3 assesses general HRQoL across domains of vision, hearing, speech, ambulation, dexterity, emotion, cognition and pain. HRQoL across those domains is scored from 1 to 6 and an overall health utility score is generated: a utility score of zero indicate worst possible HRQoL (death) and a score of one indicates best possible HRQoL (perfect health). We report here on outcomes from the parent-proxy versions of the PedsQL and HUI3 measures.

We assessed parents’ emotional well-being using the Emotion Thermometer (ET) [55, 56]. The ET has been used for monitoring emotional well-being in the context of cancer, assessing anxiety, depression, anger, and need for help on scales from 0 to 10 [57]. A score of ≥5 indicates significant distress. Additional questions ask whether an individual is currently receiving help, and if not, whether they would like additional help. For this study, parents requesting additional help were offered: (i) for the research team to flag their concerns with their treating team, (ii) to receive an email outlining recommended steps for accessing care for their concerns, or (iii) the option to speak with a psychologist on the research team for further assistance.

### Analysis

To confirm the capacity of the ASSET registry to prospectively capture TRTs and HRQoL data in the first 12 months of treatment, we analyzed TRT incidence and HRQoL outcomes. TRT frequencies were calculated to determine the incidence of TRTs in the ASSET study for comparison to the ERASE study [6, 10, 32]. Changes in children’s HRQoL and parents’ emotional well-being throughout the first year of treatment were documented. This included calculation of descriptive statistics for the parent proxy PedsQL subscale scores, the ET subscale scores, and the parent proxy PedsQL and HUI overall scores, at each time point.

To address whether the HRQoL study provided a feasible method of data collection for researchers and participants, the overall parent response rate at T1 and retention rates at each HRQoL data collection time point were calculated. Time required of the study coordinator to manage the data collection was considered. To compare HRQoL across COG and iBFM protocols, independent samples t-tests were calculated to compare the mean HRQoL or parents’ emotional well-being over time. To compare ASSET HRQoL outcomes to the literature, independent samples t-tests were conducted to compare HRQoL outcome scores across three shared timepoints.

We used minimum clinically important difference (MCID) to determine whether general HRQoL between treatment platforms is broadly equivalent or meaningfully different. To compare ASSET participant outcomes to population norms for the HUI, the MCID of 0.03 was subtracted from the age-specific population norm means to generate a threshold score [58]. We then calculated the percentage of participants scoring below that threshold at each time point.

## Results

### Patient demographics, outcome and TRT incidence

Between January 2016–September 2020, 260 pediatric ALL patients aged ≤18 years were enrolled on ASSET. Of these, 108 (41.5%) were enrolled on therapeutic trials through COG (*n* = 90), iBFM (*n* = 12) or Interfant (*n* = 6). The remaining 152 participants were treated according to published protocols from COG (*n* = 83), iBFM (*n* = 68) or Interfant (*n* = 1).

Seventy-four symptomatic TRT episodes were recorded, and three participants had ≥2 TRTs (Table 2). The TRT incidence in ASSET was 28.5%. The incidence of symptomatic venous thromboembolism, neurotoxicity, bone-related toxicity, and pancreatitis were 7.7, 11.9, 5.4 and 5.0% respectively (Table 2). Most TRTs were Grade 2–3 in severity, and other than bone toxicity, occurred within the first few months of treatment.

**Table 2:** Demographics, treatment characteristics and incidence of treatment-related toxicities in ERASE and ASSET cohorts

Baseline Information	ERASE (***n*** = 1251)		ASSET (***N*** = 260)		
	***N***	%	***N***	%	***P*** value
Median age at diagnosis + range (months)	59 (9–227)		59 (1–213)		0.4457^*^
Median duration of follow up + range (months)	79 (3–186)		28 (1–62)		< 0.001^*^
OS at 3 years	95.5 ± 0.6		98.3 ± 0.8		0.1511
LFS at 3 years	90.7 ± 0.8		97.4 ± 1.2		0.0016
EFS at 3 years	87.2 ± 1.0		96.3 ± 1.4		0.0031
Gender					
Male	671	53.6	144	55.4	0.6071^†^ (Χ^2^ 0.26)
Immunophenotype					
Pre B-cell	1082	86.5	226	86.9	0.5740^†^ (Χ^2^ 0.3)
T-cell	151	12.1	33	12.7	
MPAL/Other	18	1.4	1	0.4	
Treatment Platform					
COG	218	17.4	173	66.5	< 0.0001^†^ (Χ^2^ 233.3)
BFM	1033	82.6	80	30.8	
Interfant	0	0.0	7	2.7	
Risk Classification					
Low/Standard Risk	515	41.2	82	31.5	< 0.0001^†^ (Χ^2^ 20.8)
Medium/Average/ Intermediate Risk	446	35.7	83	31.9	
High/Very High Risk	264	21.1	81	31.2	
Unknown	26	2.1	14	5.4	
Toxicity					
Symptomatic venous thromboembolism	68	5.4	20	7.7	0.1488
Neurotoxicity (≥ grade 3)	111	8.9	31	11.9	0.1319
Bone	239	19.1	14	5.4	0.0001
Pancreatitis	48	3.8	13	5.0	0.3695

*OS* Overall survival, *LFS* Leukaemia Free Survival, *EFS* Event Free Survival

^*^Mann-Whitney Test (independent samples)

^†^Chi-squared test

The baseline demographic, treatment characteristics, and toxicity data from this prospective study were broadly comparable. The major differences were the shorter follow-up, the inclusion of patients with infant ALL and a higher proportion of patients with high/very high risk ALL in ASSET (Table 2). In ERASE, the incidence of symptomatic TRTs was in keeping with previous published reports, with venous thromboembolism in 5.4% [3, 6, 32, 59], ≥grade 3 neurotoxicity (central and/or peripheral) in 8.9% [8, 10, 60, 61], bone toxicity (avascular necrosis and/or fractures) in 19.1% [62, 63] and pancreatitis in 3.8% of patients [64, 65]. There was no significant difference in the incidence of symptomatic venous thromboembolism, neurotoxicity and pancreatitis between ERASE and ASSET (Table 2). There was a significantly lower incidence of bone toxicity in the ASSET study (Table 2) which most likely reflected the shorter duration of follow-up.

### Longitudinal HRQoL and emotional wellbeing in the first 12 months of ALL treatment

Recruitment to the ASSET HRQoL study commenced in October 2018. Figure 1 summarises participation in the HRQoL study. Eighty ASSET participants, enrolled prior to October 2018, did not participate in the HRQoL study and were excluded leaving 156 participants eligible for the HRQoL study (Fig. 1). Of these, 79 parents (overall response rate 50.6%) consented and provided HRQoL data on at least one survey in the first 12 months of their child’s treatment. Parents represented children whose average age at diagnosis was 5.7 years (Table 3).

**Table 3:** Demographics, treatment characteristics and questionnaire completion of the HRQoL cohort (*N* = 79 parents)

Demographics	Child/young person’s Age at Diagnosis	*M* = 5.74(*SD* = 3.81), *R* = 1.03–17.20
	Child/young person’s Gender	25 females (31.6%) 54 males (68.4%)
	Distance from Treatment Center	M = 191.9 km (SD = 479.6), R = 4.4–3922
Immunophenotype	Pre B-cell	64 (81.0%)
	T-cell	15 (19.0%)
	MPAL/Other	0 (0%)
Treatment Platform	BFM	26 (32.9%)
	COG	53 (67.1%)
Risk Classification	Low/Standard Risk	33 (41.8%)
	Medium/Average/ Intermediate Risk	22 (27.8%)
	High/Very High Risk	14 (17.7%)
Total Number of Questionnaires out of a possible 12 Completed by Participants	*Number of questionnaires*	*Number of participants*
	1	5 (6.3%)
	2	4 (5.1%)
	3	7 (8.9%)
	4	7 (8.9%)
	5	7 (8.9%)
	6	9 (11.4%)
	7	9 (11.4%)
	8	10 (12.7%)
	9	8 (10.1%)
	10	9 (11.4%)
	11	2 (2.5%)
	12	1 (1.3%)

Response rates across each HRQoL assessment varied from 66.7–85.7% (Fig. 1, Supplementary Table 1). Response rates were lowest at T4 (66.7%, M = 4.4 months post-diagnosis). On an individual response level, only one parent completed all 12 questionnaires with most parents missing at least two time points and completing fewer than 10/12 questionnaires (Table 3). The main reported reason for missed surveys was forgetting/not checking emails. Following enrolment, five families withdrew due to being too busy or distressed and 21 families were lost to contact. Two children died during the study. At least 7 h per week were required for the HRQoL study coordinator to manage the study.

**Fig. 2:**
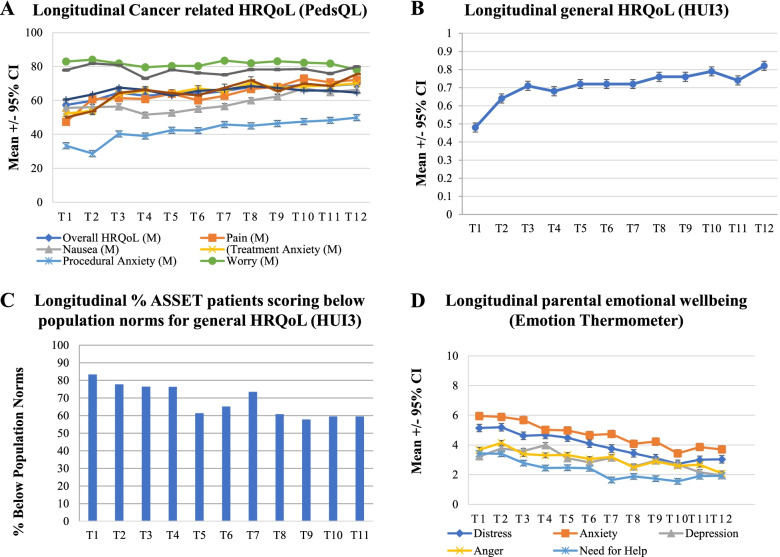
Longitudinal HRQoL and emotional wellbeing in ASSET participants. **A** Pediatric Quality of Life Inventory – Cancer Module: cancer-specific health-related quality of life in the first year of treatment. Mean PedsQL score +/− 95% CI; increased score = increased HRQoL **B** Health Utilities Index 3: health-related quality of life in the first year of treatment. Mean HUI3 score +/− 95% CI; increased score = increased HRQoL. **C** Percentage of ASSET patients with general HRQoL below the population at each time point in the first 12 months of ALL therapy. **D** Parental emotional well-being over time measured by emotion thermometer. Mean Emotional Well-being score +/− 95% CI; increased score = increased emotional concern

Analysis of parent-proxy reports demonstrated that cancer-related HRQoL (PedsQL) was poor over time, remaining ≤70/100 (Fig. 2A, Supplementary Table 1). Procedural anxiety had a consistently negative impact on HRQoL, with average scores ≤50/100 across all time points (Fig. 2A). Nausea was a consistent contributor to poor HRQoL. Average general HUI3 scores increased over time (Fig. 2B). More than 50% of parent proxy reports of children’s general HRQoL remained below the minimally clinically important difference (MCID) threshold in comparison to population norms across all time points (Fig. 2C). Parents’ emotional well-being was poorest in the first 6 months post-diagnosis. Anxiety was the most highly rated concern and need for help was greatest in the earlier months of treatment. Generally, we observed that parents’ average scores on the emotion thermometers decreased, indicating improvement in mental health over time, as did their need for help (Fig. 2D, Supplementary Table 2). Parents most frequently requested further help for emotional concerns in the first 6 months post-diagnosis (Supplementary Table 2). Seventeen parents (21.5%) requested help once from T1-T12, and three parents (3.8%) requested help twice. Regarding preferred support, 16 parents requested email support, three requested emotional concerns to be flagged with their child’s treating team, and six requested further help but not via any of the options listed.

### Comparison of longitudinal HRQoL and emotional wellbeing between COG and iBFM treatment platforms in the first 12 months of ALL treatment

Parents of children treated according to a COG protocol reported their child experienced significantly poorer cancer-specific HRQoL than the iBFM group at some discrete time points (Supplementary Table 3). At T3, COG parents reported their child experienced significantly poorer HRQoL due to physical appearance (*M* = 76.92, *SD =* 26.70, *M =* 3.36 months post-diagnosis) than iBFM parents (*M* = 93.75, *SD =* 11.57, *t*_28_ = − 2.53, *p* = 0.02). At T9 and T10, COG parents reported their child experienced significantly poorer HRQoL due to nausea (T9 *M* = 54.63, *SD* = 26.20, *M =* 9.46 months post-diagnosis; T10 *M =* 59.20, *SD =* 27.07, *M* = 10.49 months post-diagnosis) than iBFM parents (T9 *M* = 73.89, *SD =* 19.97, *t*_*43*_ *= −* 2.65). Similarly, parents with a child treated according to a COG protocol reported their child experienced significantly poorer general HRQoL on the HUI3 compared to the iBFM group at some discrete time points: T5 (COG *M* = 0.67, iBFM *M* = 0.83, *t*_*40.87*_ *=* − 2.51. *p* = 0.02), T9 (COG *M* = 0.70, iBFM *M* = 0.88, *t*_40.35_ = − 2.86, *p* = 0.007), and T10 (COG *M* = 0.74, iBFM *M* = 0.89, *t*_39.63_ = − 2.66, *p* = 0.01) (Supplementary Tables 3 and 4). However, there was no evidence of persisting significant differences in the measured cancer specific or general HRQoL of the different treatment platforms over multiple consecutive time points. Some differences in the emotional well-being of parents of patients treated on each of the two protocols were identified (Supplementary Table 5). At T2, iBFM parents reported significantly more depression than COG parents (iBFM *M* = 5.18, COG *M* = 3.15, *t*_35_ = − 2.02, *p =* 0.05). At T3 iBFM parents also reported significantly more need for help than COG parents (iBFM *M* = 4.00, COG *M =* 2.37, *t*_*34*_ = 2.00, *p* = 0.05). At T5, T8, and T11 COG parents reported significantly more anger than iBFM parents (T5 COG *M* = 3.87, iBFM *M* = 2.14, t_35.56_ = 2.32, *p* = 0.03, T8 COG *M* = 3.03, iBFM *M* = 1.50, *t*_48.21_ = 2.36, *p* = 0.02, T11 COG *M* = 3.21, iBFM *M* = 1.67, *t*_37.04_ = 2.16*, p* = 0.04). Lastly, at T10, COG parents reported significantly more distress than iBFM parents (COG *M* = 3.39, iBFM *M* = 1.56, *t*_42_ = 2.17, *p* = 0.04). However, there was no evidence of persisting significant differences in the measured parental emotional wellbeing of the different treatment platforms over multiple consecutive time points.

## Discussion

The results support the capacity of a prospective registry to document TRT, longitudinal HRQoL, and parents’ emotional well-being for children and adolescents treated for ALL across Australia and New Zealand. In ASSET, the incidence of individual TRTs is consistent with the available literature and the retrospective ERASE cohort [3–15, 32]. One discrepancy relates to bone toxicity, with 5.4% reported in ASSET and 19.1% in the ERASE cohort. Given that most bone toxicity occurs > 1 year into ALL treatment, the apparent low incidence of bone toxicity in ASSET is likely due to the shorter duration of follow-up.

We demonstrated the capacity to capture longitudinal HRQoL and emotional well-being data, providing a clear picture of change in HRQoL and well-being over time. Improvement in general HRQoL (HUI3) was contrasted by a lack of improvement in cancer-related HRQoL (PedsQL). Although there is improvement in general HRQoL with time, most families report general HRQoL below population norms throughout the entire first year of ALL therapy highlighting the sustained impact of ALL therapy. Parents’ emotional well-being also demonstrated improvement over time, although anxiety remained a consistent concern.

Although we observed discrete differences between protocol groups at some timepoints for HRQoL and emotional wellbeing, this needs to be interpreted with caution. First, we did not observe a consistent pattern of persisting difference in measures of HRQoL over multiple consecutive time points. Second we did not control for any potential confounders such as child sex and other sociodemographic factors previously shown to impact HRQoL due to sample size [20]. Furthermore, the confidence intervals for the differences in general HRQoL (HUI3) include plausible values larger than the MCID of 0.03 in both directions, suggesting that the sample size may not be sufficient to reject the possibility that there exist small but meaningful differences between protocols. On the PedsQL, the significant differences we observed at T9 and T10 seemed inconsistent with other time points and therefore could be attributed as type I errors. Taken together this may suggest that the impact of COG or iBFM based therapy on HRQoL and parental emotional wellbeing during the first 12 months of ALL treatment is no different. It is reassuring that we did not observe persistent longitudinal HRQoL and emotional wellbeing differences between these different treatment programs in the first 12 months of ALL therapy as there is substantial similarity in the intensity of treatment during this period. However, analysis of HRQoL and emotional wellbeing in the second year of treatment and beyond is likely to be informative, as this is the time period where the substantial differences in the duration of treatment (total treatment duration 2 years versus 3.5 years) and the intensity of maintenance chemotherapy (pulsed dexamethasone, intravenous vincristine and lumbar punctures versus oral chemotherapy only) may result in differing HRQoL impacts. Dutch HRQoL data suggests that prolonged and intensive maintenance therapy results in reduced HRQoL [31].

There are limitations to the HRQoL sub-study. Response rates across monthly assessments of HRQoL remained ≥60%, consistent with that of previous studies such as the UKALL2003 clinical trial (63%) [29, 30]. However, follow-up logs indicated that parents frequently missed surveys due to forgetting or not checking emails. These findings suggest room to improve the feasibility of data collection. The early months of treatment can be a busy, overwhelming time for families, when many TRTs occur. While frequent assessment can provide useful indicators of HRQoL, it creates additional burden for parents that may affect participation. Time and staff costs for study management should be considered as well. Bi-monthly or less frequent assessment may improve the feasibility of longitudinal HRQoL data collection, providing similarly useful information, with reduced burdens. One previous longitudinal study of children’s HRQoL on active treatment for ALL included only four assessment time points and reported higher retention rates across similar time points (90% at T1 to 63% at T4) to our study [29]. There is also the possibility that the thematic information for HRQoL might be different at T1 than at T6 and hence, missing some of the earlier time points may skew the results. Utilization of iBFM versus COG protocols is site specific and there are likely systematic differences in the support and resources available to families at each site, which could affect HRQoL outcomes. There is a relative deficiency in data pertaining to site-specific resource differences which has the potential to influence and skew results, with the relatively small sample size not amenable to cluster analysis. Despite these limitations, there are several strengths of this HRQoL sub-study. It is prospective in nature and although potentially burdensome, the frequent, robust questionnaires provide an ideal opportunity to thoroughly track HRQoL across the early phase of treatment. This patient population also provides a unique opportunity to prospectively compare two contemporary treatment platforms, with respect to TRT and HRQoL.

Three longitudinal studies have assessed cancer-related HRQoL in pediatric ALL using the PedsQL cancer module [23, 27, 29, 30], and two (COG [27, 29] and UKALL [30]) reported outcomes more frequently than pre- and post-maintenance [27, 30]. The cancer-related HRQoL outcomes in ASSET differed significantly from COG and UKALL findings across the 1 month, 6 months and 12 months post-diagnosis, particularly in the domains of nausea, pain, and overall HRQoL [27, 30]. There are notable differences between the ASSET, COG, and UKALL studies, including participant age (ASSET = 0–18, COG = 2–10, UKALL = 4–18), treatment risk groups (ASSET all risk groups, COG standard risk only, UKALL all risk groups), and clinical trial involvement (ASSET a combination of clinical trial (41.5%) and standard treatment compared to patients enrolled on clinical trials for the COG and UKALL).

Compared to the COG study, in the first month post-diagnosis and 6 months post-diagnosis, our sample reported similar pain, treatment anxiety, and procedural anxiety, but significant nausea [27]. At 12 months post-diagnosis, our sample reported similar pain and treatment anxiety, but poorer HRQoL due to nausea and procedural anxiety [27]. While the UKALL study did not report on cancer-related HRQoL subscales, worse overall cancer-related HRQoL at 4 weeks post-diagnosis was reported compared to our T1 (1 month post-diagnosis) results. Additionally, in the UKALL study, overall cancer-related HRQoL at T3 (24–47 weeks = 5–12 months) was similar to our T5-T8 results but poorer than our T9-T12 results.

ALL therapy impacts HRQoL and therefore collecting HRQoL data throughout treatment is important to improve care for patients and families. The finding regarding the lack of improvement in procedural anxiety may indicate a lack of intervention or support for these concerns in early treatment which could be addressed with existing, well-established, non-pharmacological interventions, such as music therapy or distraction [66, 67]. The finding regarding parents’ need for help during the first year of ALL treatment also suggests that healthcare providers have an opportunity to address parents’ emotional concerns and foster improved well-being, given it is a time when they have frequent contact with children and families. Improving our understanding of ‘real-time’ needs for this population could assist in implementing targeted support strategies. Future research priorities for the ASSET study include examining HRQoL in the 2nd and subsequent years after a diagnosis of ALL; a comparison of the short, medium and long term treatment related toxicity incidence and patterns in patients treated on iBFM and COG platforms with a specific focus of the HRQoL experience related to the duration and intensity of maintenance therapy; the impact of an ALL diagnosis, treatment and toxicity on educational achievement; a health economic analysis of the cost of ALL therapy including the additional costs of managing treatment related toxicities arising from ALL therapy. Health economic and educational outcome analyses will be undertaken by data linkage between the ASSET study with population linked administrative data sets.

With long-term survival rates for pediatric ALL reaching new highs, it is important to understand and improve the treatment experience for children and their families, to optimize both immediate and long-term well-being. Our findings (i) validate the capacity of the ASSET study to capture TRTs, children’s HRQoL, and parents’ emotional well-being, (ii) demonstrate poor ongoing cancer-related HRQoL and increased anxiety among parents in the first 12 months for children on ALL treatment, (iii) suggest there are no major differences in early HRQoL impact between different treatment platforms in Australia and New Zealand, and (iv) suggest the HRQoL study feasibility can be improved to increase response and retention rates. We aim to continue recruitment but to refine the HRQoL study. It will be important to optimize the timing of HRQoL assessment such that it can be both clinically informative and less burdensome for families and researchers alike.

## Supplementary Information

**Additional file 1: Supplementary Table 1.** ASSET Health-Related Quality of Life Outcomes (PedsQL Cancer Module and HUI3). **Supplementary Table 2.** Parents’ emotional well-being (ET). **Supplementary Table 3.** COG vs. iBFM Protocol Group Health-Related Quality of Life Comparisons (HUI3, PedsQL & PedsQL Nausea, Pain & Procedural Anxiety subscales). **Supplementary Table 4.** COG vs. iBFM Protocol Group Health Related Quality of Life Comparisons (PedsQL Anxiety, Worry, Cognitive Functioning, Physical appearance & Communication subscales). **Supplementary Table 5.** COG vs. iBFM Protocol group parental emotional well-being comparisons (ET).

**Additional file 2: Data Table 1.** HUI3 Data. **Data Table 2.** PedsQL Cancer Data. **Data Table 3.** Emotion Thermometer Data.

## Data Availability

The data that support the findings of this study are available in the Supplementary files.

## Abbreviations

AIEOP-BFM: Associazione Italiana Ematologia Oncologia Pediatrica – Berlin-Franklin-Munster protocol
iBFM: International Berlin-Franklin-Munster study group
ALL: Acute Lymphoblastic Leukemia
ANZCCSG: Australian and New Zealand Children’s Cancer Study Group
ANZCHOG: Australian and New Zealand Children’s Haematology Oncology Group
ASSET: Acute Lymphoblastic Leukemia Sub-types and Side Effects from Treatment Study
COG: Children’s Oncology Group
ERASE: Evaluation of Risk of ALL Treatment-Related Side Effects
HNE HREC: Hunter New England Human Research Ethics Committee
HRQoL: Health-related quality of life
HUI3: Health Utility Index 3
MPAL: Mixed Phenotype Acute Leukaemia
PedsQL: Pediatric Quality of Life Inventory
REDCap: Research Electronic Data Capture
TRT: Treatment-related toxicity
