# Supplementary material for: Prospective longitudinal evaluation of treatment-related toxicity and health-related quality of life during the first year of treatment for pediatric acute lymphoblastic leukemia
Source: BMC Cancer. 2022 Sep 15;22:985. doi: 10.1186/s12885-022-10072-x (PMC9479356; doi:10.1186/s12885-022-10072-x)
Supplement: Supplementary file 1 — Additional file 1: Supplementary Table 1. ASSET Health-Related Quality of Life Outcomes (PedsQL Cancer Module and HUI3). Supplementary Table 2. Parents’ emotional well-being (ET). Supplementary Table 3. COG vs. iBFM Protocol Group Health-Related Quality of Life Comparisons (HUI3, PedsQL & PedsQL Nausea, Pain & Procedural Anxiety subscales). Supplementary Table 4. COG vs. iBFM Protocol Group Health Related Quality of Life Comparisons (PedsQL Anxiety, Worry, Cognitive Functioning, Physical appearance & Communication subscales). Supplementary Table 5. COG vs. iBFM Protocol group parental emotional well-being comparisons (ET). [file 12885_2022_10072_MOESM1_ESM.docx]

| SUPPLEMENTARY TABLE 1 ASSET Health-Related Quality of Life Outcomes (PedsQL Cancer Module and HUI3) | | | | | | | | | | | | |
| --- | --- | --- | --- | --- | --- | --- | --- | --- | --- | --- | --- | --- |
| **Time** | **Response Rate** | **Time since diagnosis (M(SD), Range, months)** | **HUI HRQoL (M(SD), Range)** | **PedsQL Overall**  **(M(SD), Range)** | **PedsQL Nausea**  **(M(SD), Range)** | **PedsQL Pain (M(SD), Range)** | **PedsQL Procedural anxiety**  **(M(SD), Range)** | **PedsQL Treatment anxiety**  **(M(SD), Range)** | **PedsQL Worry**  **(M(SD), Range)** | **PedsQL Cognitive functioning (M(SD), Range)** | **PedsQL Physical appearance (M(SD), Range)** | **PedsQL Communication (M(SD), Range)** |
| T1 | 24/29  82.75% | 1.26(0.46),  0.39-1.95 | 0.48(0.29)  -0.17-1 | 57.34(17.72),  22.60-99.38 | 55.63(23.42),  0-100 | 47.39(27.58),  0-100 | 33.33(30.10),  0-100 | 52.26(32.90),  0-100 | 82.99(23.25),  25-100 | 60.50(25.04),  0-100 | 77.90(27.25),  0-100 | 50.00(35.95),  0-100 |
| T2 | 36/42  85.71% | 2.40(0.43),  1.25-2.96 | 0.64(0.25)  -0.25-1 | 59.85(16.88),  23.19-99.38 | 56.11(20.19),  15-100 | 60.76(17.97),  25-100 | 28.70(29.71),  0-100 | 53.94(32.46),  0-100 | 84.07(24.56),  16-100 | 63.54(25.13),  0-100 | 81.91(21.81),  8.33-100 | 53.70(37.50),  0-100 |
| T3 | 34/47  (72.34%) | 3.36(0.36),  2.56-3.96 | 0.71(0.26),  -0.05-1 | 64.59(17.30),  26.25-99.38 | 56.47(23.01),  15-95 | 61.39(23.31),  25-100 | 40.31(32.45),  0-100 | 63.73(28.57),  0-100 | 81.87(24.40),  16.67-100 | 67.59(21.50),  18.75-100 | 80.88(24.92),  25-100 | 64.46(35.43),  0-100 |
| T4 | 38/57  (50.00%) | 4.44(0.36),  3.62-4.99 | 0.68(0.30),  0.01-1 | 62.80(14.04),  31.61-92.19 | 51.64(18.58),  5-100 | 60.86(20.58),  25-100 | 39.04(26.85),  0-100 | 65.79(25.97),  0-100 | 79.61(24.86),  0-100 | 66.22(19.14),  31.25-100 | 73.03(23.12),  25-100 | 66.23(30.69),  0-100 |
| T5 | 44/59  (74.58%) | 5.42(0.33),  4.62-5.95 | 0.72(0.24),  0.11-1 | 63.74(15.46),  30.73-90.63 | 52.76(18.89),  20-100 | 63.95(20.08),  12.50-100 | 42.46(29.95),  0-100 | 64.34(25.41),  0-100 | 80.42(25.45),  8.33-100 | 62.79(20.21),  25-100 | 78.10(26.73),  0-100 | 64.34(28.07),  0-100 |
| T6 | 42/56  (75.00%) | 6.43(0.33),  5.98-6.96 | 0.72(0.25),  0-1 | 63.76(17.71),  20.42-91.25 | 55.09(21.35),  20-100 | 60.11(21.78),  25-100 | 42.26(34.59),  0-100 | 67.07(29.45),  0-100 | 80.35(23.98),  8.33-100 | 65.70(23.71),  25-100 | 76.23(22.01),  16.67-100 | 63.09(34.83),  0-100 |
| T7 | 48/62  (77.42%) | 7.42(0.36),  6.77-7.93 | 0.72(0.27)  0.11-1 | 65.60(19.65),  29.79-100 | 56.67(22.55),  20-100 | 62.76(22.84),  0-100 | 45.83(36.30),  0-100 | 65.45(32.34),  0-100 | 83.51(21.94),  25-100 | 66.54(24.22),  8.33-100 | 75.18(27.33),  0-100 | 67.55(31.77),  0-100 |
| T8 | 50/63  (79.37%) | 8.44(0.37),  7.52-8.99 | 0.76(0.23)  0.16-1 | 68.06(17.94),  25.94-100 | 60.10(22.96),  10-100 | 66.75(21.22),  25-100 | 45.07(33.02),  0-100 | 70.33(27.52),  0-100 | 82.00(24.24),  8.33-100 | 68.46(23.07),  18.75-100 | 78.23(24.76),  0-100 | 71.94(25.84),  25-100 |
| T9 | 45/63  (71.42%) | 9.46(0.32),  8.54-9.94 | 0.76(0.26)  -0.07-1 | 67.69(18.98),  14.22-100 | 62.33(25.51),  0-100 | 68.06(21.41),  0-100 | 46.41(30.31),  0-100 | 67.41(29.35),  0-100 | 83.14(24.08),  0-100 | 67.42(22.37),  6.25-100 | 78.22(25.81),  0-100 | 65.53(32.87),  0-100 |
| T10 | 42/62  (67.74%) | 10.49(0.34),  9.89-10.99 | 0.79(0.22),  0.06-1 | 69.31(19.54),  26.88-100 | 67.10(25.77),  5-100 | 73.01(19.43),  12.50-100 | 47.54(32.68),  0-100 | 68.37(32.02),  0-100 | 82.36(23.86),  25-100 | 65.89(23.60),  0-100 | 78.49(22.87),  25-100 | 69.96(29.55),  0-100 |
| T11 | 42/58  (72.41%) | 11.46(0.35),  10.69-11.99 | 0.74(0.26),  -0.08-1 | 68.64(19.32),  15.68-100 | 64.83(25.98),  0-100 | 70.74(22.06),  25-100 | 48.30(37.16),  0-100 | 68.75(30.61),  0-100 | 81.82(24.26),  0-100 | 65.86(23.76),  18.75-100 | 75.95(26.30),  8.33-100 | 68.64(19.33),  15.68-100 |
| T12 | 40/56  (71.42%) | 12.65(1.09),  11.67-13.57 | 0.82(0.19),  0.28-1 | 69.77(17.44),  35.26-100 | 66.34(24.52),  20-100 | 72.19(18.88),  25-100 | 49.90(32.82),  0-100 | 70.12(29.40),  0-100 | 78.05(24.27),  25-100 | 64.63(23.34),  0-100 | 79.88(22.12),  25-100 | 75.61(25.85),  0-100 |

SUPPLEMENTARY TABLE 2 Parents’ emotional well-being (ET)

| **Time** | **Distress (M(SD), Range)** | **Anxiety (M(SD), Range)** | **Depression (M(SD), Range)** | **Anger (M(SD), Range)** | **Need for Help (M(SD), Range)** | **Requested Further Help(N)** |
| --- | --- | --- | --- | --- | --- | --- |
| T1 | 5.14(2.55),  0-10 | 5.95(2.40),  2-9 | 3.23(2.43),  0-8 | 3.68(2.59),  0-9 | 3.43(2.62),  0-9 | 3 |
| T2 | 5.19(2.92),  0-10 | 5.89(2.64),  0-10 | 3.76(2.91),  0-10 | 4.14(2.85),  0-10 | 3.41(2.77),  0-10 | 7 |
| T3 | 4.62(2.89),  0-10 | 5.68(2.69),  0-10 | 3.59(2.81),  0-9 | 3.39(2.72),  0-8 | 2.78(2.21),  0-9 | 1 |
| T4 | 4.67(2.52),  0-8 | 5.02(2.68),  0-10 | 3.98(2.67),  0-8 | 3.30(2.53),  0-8 | 2.45(2.49),  0-8 | 2 |
| T5 | 4.49(3.10),  0-9 | 4.98(3.00),  0-10 | 3.11(2.88),  0-10 | 3.33(2.75),  0-10 | 2.47(2.38),  0-9 | 2 |
| T6 | 4.09(2.96),  0-10 | 4.66(2.96),  0-10 | 2.82(2.90),  0-10 | 3.05(2.79),  0-10 | 2.43(2.34),  0-10 | 3 |
| T7 | 3.76(2.97),  0-10 | 4.74(2.88),  0-10 | 3.14(3.06),  0-10 | 3.20(2.89),  0-10 | 1.64(2.21),  0-9 | 4 |
| T8 | 3.44(2.91),  0-10 | 4.08(2.90),  0-10 | 2.54(2.67),  0-10 | 2.49(2.62),  0-10 | 1.88(2.47),  0-10 | 1 |
| T9 | 3.11(2.63),  0-9 | 4.23(2.77),  0-9 | 2.95(2.53),  0-9 | 2.89(2.38),  0-8 | 1.73(2.32),  0-8 | 0 |
| T10 | 2.73(2.81),  0-10 | 3.43(2.88),  0-10 | 2.68(2.76),  0-10 | 2.59(2.64),  0-9 | 1.55(2.44),  0-8 | 0 |
| T11 | 3.00(2.48),  0-8 | 3.86(2.69),  0-10 | 2.16(2.43),  0-8 | 2.67(2.54),  0-8 | 1.90(2.34),  0-8 | 2 |
| T12 | 3.03(2.52),  0-8 | 3.70(2.84),  0-9 | 1.95(2.11),  0-7 | 2.08(2.31),  0-8 | 1.92(2.56),  0-9 | 0 |

SUPPLEMENTARY TABLE 3. COG vs. iBFM Protocol Group Health-Related Quality of Life Comparisons (HUI3, PedsQL & PedsQL Nausea, Pain & Procedural Anxiety subscales)

| **Time** | **Protocol Group** | **HUI HRQoL (M(SD))** | **T-test p and**  **95% CI** | **PedsQL Total**  **(M(SD))** | **T-test p and**  **95% CI** | **PedsQL Nausea**  **(M(SD))** | **T-test p and**  **95% CI** | **PedsQL Pain (M(SD))** | **T-test p and**  **95% CI** | **PedsQL Procedural anxiety (M(SD))** | **T-test p and**  **95% CI** |
| --- | --- | --- | --- | --- | --- | --- | --- | --- | --- | --- | --- |
| T1 | COG (N=17) | 0.52(0.31) | p=0.43  -0.17-0.39 | 57.47(19.59) | p=0.96  -16.44-17.31 | 53.24(26.16) | p=0.45  -30.20-13.81 | 49.26(29.14) | p=0.62  -19.70-32.52 | 31.37(32.74) | p=0.63  -35.23-21.78 |
|  | AIEOP-BFM (N=7) | 0.41(0.27) |  | 57.03(13.43) |  | 61.43(14.92) |  | 42.86(24.85) |  | 38.09(23.99) |  |
| T2 | COG  (N=26) | 0.65(0.28) | p=0.69  -0.15-0.23 | 60.51(19.11) | p=0.73  -10-39-14.75 | 59.40(22.09) | p=0.07  -1.23-22.75 | 62.20(18.91) | p=0.54  -9.28-17.37 | 33.67(31.96) | p=0.13  -5.19-37.67 |
|  | AIEOP-BFM (N=10) | 0.61(0.16) |  | 58.33(10.82) |  | 48.64(12.86) |  | 57.95(16.08) |  | 17.42(20.90) |  |
| T3 | COG (N=24) | 0.71(0.27) | p=0.80  -0.18-0.23 | 64.32(17.59) | p=0.87  -15.60-13.33 | 57.12(23.50) | p=0.77  -16.48-21.96 | 60.56(22.83) | p=0.72  -22.94-15.97 | 43.75(31.60) | p=0.27  -12.04-41.21 |
|  | AIEOP-BFM  (N=10) | 0.69(0.25) |  | 65.46(17.45) |  | 54.37(22.75) |  | 64.06(26.25) |  | 29.17(34.79) |  |
| T4 | COG (N=28) | 0.64(0.31) | p=0.20  -0.36-0.08 | 61.50(13.45) | p=0.34  -15.47-5.54 | 48.88(16.29) | p=0.13  -24.11-3.13 | 58.04(18.70) | p=0.16  -25.88-4.45 | 39.58(28.20) | p=0.84  -18.24-22.41 |
|  | AIEOP-BFM (N=10) | 0.78(0.26) |  | 66.46(15.73) |  | 59.38(23.09) |  | 68.75(24.47) |  | 37.50(23.98) |  |
| T5 | COG (N=30) | 0.67(0.27) | p=0.02*  -0.29- -0.03 | 61.42(15.69) | p=0.21  -16.71-3.78 | 49.60(18.27) | p=0.17  -21.05-3.82 | 61.16(19.35) | p=0.21  -21.82-4.86 | 38.27(30.51) | p=0.18  -21.82-4.86 |
|  | AIEOP-BFM (N=14) | 0.83(0.14) |  | 67.88(15.06) |  | 58.21(19.87) |  | 69.64(21.77) |  | 51.79(28.53) |  |
| T6 | COG (N=27) | 0.76(0.20) | p=0.27  -0.08-0.27 | 63.00(17.31) | p=0.64  -14.09-8.78 | 53.39(22.73) | p=0.42  -19.25-8.20 | 59.38(19.43) | p=0.74  -16.34-11.77 | 41.67(34.54) | p=0.96  -22.97-21.87 |
|  | AIEOP-BFM (N=16) | 0.66(0.30) |  | 65.66(18.43) |  | 58.92(17.99) |  | 61.67(25.65) |  | 34.62(36.30) |  |
| T7 | COG (N=31) | 0.72(0.27) | p=0.69  -0.13-0.20 | 64.58(20.15) | p=0.65  -15.47-5.54 | 52.67(24.10) | p=0.11  -23.98-2.65 | 64.58(21.80) | p=0.48  -8.92-18.64 | 41.67(36.09) | p=0.31  -32.88-10.66 |
|  | AIEOP-BFM (N=18) | 0.69(0.28) |  | 67.31(19.22) |  | 63.33(18.47) |  | 59.72(24.84) |  | 52.78(36.60) |  |
| T8 | COG (N=35) | 0.77(0.26) | p=0.73  -0.11-0.17 | 67.86(18.71) | p=0.92  -11.46-10.30 | 59.55(25.57) | p=0.82  -15.55-12.28 | 65.53(21.88) | p=0.58  -16.42-9.24 | 50.26(34.55) | p=0.13  -4.70-34.63 |
|  | AIEOP-BFM (N=16) | 0.74(0.24) |  | 68.45(16.91) |  | 61.18(17.46) |  | 69.12(20.30) |  | 35.29(28.34) |  |
| T9 | COG (N=28) | 0.70(0.29) | p=0.007*  -0.31- -0.05 | 64.90(18.59) | p=0.23  -18.55-4.63 | 54.63(26.20) | p=0.01*  -33.94- - 4.57 | 63.89(22.29) | p=0.11  -23.32-2.48 | 46.91(30.32) | p=0.34  -17.83-20.48 |
|  | AIEOP-BFM (N=17) | 0.88(0.13) |  | 71.87(19.34) |  | 73.89(19.97) |  | 74.31(18.92) |  | 45.59(31.20) |  |
| T10 | COG (N=28) | 0.74(0.25) | p=0.01*  -0.26- -0.03 | 64.89(19.53) | p=0.23  -18.55-4.62 | 59.20(27.07) | p=0.05*  -24.08- -0.26 | 66.52(18.65) | p=0.002*  -34.93- -8.56 | 44.05(31.33) | p=0.35  -28.97- -6.73 |
|  | AIEOP-BFM (N=14) | 0.89(0.11) |  | 77.07(17.52) |  | 80.94(16.25) |  | 84.38(15.49) |  | 53.65(35.09) |  |
| T11 | COG (N=30) | 0.73(0.26) | p=0.77  -0.21-0.16 | 66.59(20.10) | p=0.33  -19.16-6.56 | 60.17(27.30) | p=0.11  -30.50-3.35 | 68.97(23.53) | p=0.49  -19.89-9.61 | 46.55(38.55) | p=0.78  -28.28-21.39 |
|  | AIEOP-BFM (N=12) | 0.76(0.29) |  | 72.89(18.35) |  | 73.75(22.03) |  | 74.11(19.89) |  | 50.00(36.69) |  |
| T12 | COG (N=27) | 0.78(0.20) | p=0.13  -0.22-0.03 | 68.52(17.92) | p=0.34  -16.58-5.84 | 63.20(24.62) | p=0.16  -26.73-4.46 | 71.35(19.67) | p=0.46  -16.73-7.77 | 47.05(36.38) | p=0.41  -31.16-13.04 |
|  | AIEOP-BFM (N=13) | 0.88(0.12) |  | 73.89(15.17) |  | 74.33(21.70) |  | 75.83(15.99) |  | 56.11(26.99) |  |

*Significant difference (p<0.05)

SUPPLEMENTARY TABLE 4 COG vs. iBFM Protocol Group Health Related Quality of Life Comparisons (PedsQL Anxiety, Worry, Cognitive Functioning, Physical appearance & Communication subscales)

| **Time** | **Protocol Group** | **PedsQL Treatment anxiety**  **(M(SD))** | **T-test**  **p and**  **95% CI** | **PedsQL Worry**  **(M(SD))** | **T-test**  **p and**  **95% CI** | **PedsQL Cognitive functioning (M(SD))** | **T-test**  **p and**  **95% CI** | **PedsQL Physical appearance (M(SD))** | **T-test**  **p and**  **95% CI** | **PedsQL Communication (M(SD))** | **T-test**  **p and**  **95% CI** |
| --- | --- | --- | --- | --- | --- | --- | --- | --- | --- | --- | --- |
| T1 | COG (N=17) | 54.17(34.49) | p=0.67  -24.65-37.74 | 81.86(25.04) | p=0.72  -25.92-18.21 | 60.91(26.07) | p=0.91  -22.46-25.22 | 78.51(23.29) | p=0.47  -17.64-36.76 | 48.52(36.47) | p=0.76  -39.20-29.11 |
|  | AIEOP-BFM (N=7) | 47.61(30.70) |  | 85.71(19.67) |  | 59.52(24.26) |  | 79.63(25.72) |  | 53.57(37.22) |  |
| T2 | COG (N=18) | 55.00(32.10) | p=0.77  -20.70-27.67 | 81.25(26.95) | p=0.22  -25.32-6.15 | 60.17(26.58) | p=0.23  -29.39-7.30 | 80.56(24.16) | p=0.60  -20.62-12.03 | 56.67(37.50) | p=0.48  -18.08-37.47 |
|  | AIEOP-BFM (N=9) | 51.52(34.73) |  | 90.83(16.87) |  | 71.21(20.53) |  | 84.84(16.17) |  | 46.97(38.42) |  |
| T3 | COG (N=24) | 67.71(25.46) | p=0.18  -6.78-35.54 | 79.49(25.84) | p=0.31  -30-.18-9.98 | 65.63(23.13) | p=0.35  -26.06-9.39 | 76.92(26.70) | p=0.02*  -30.44- -3.21 | 65.06(36.52) | p=0.86  -27.05-32.18 |
|  | AIEOP-BFM (N=10) | 53.33(32.44) |  | 89.58(18.23) |  | 73.96(14.39) |  | 93.75(11.57) |  | 62.50(33.92) |  |
| T4 | COG (N=18) | 56.86(31.07) | p=0.58  -32.69-18.64 | 64.88(26.97) | p=0.67  -22.71-15.87 | 65.10(19.65) | p=0.55  -18.70-10.16 | 71.13(21.57) | p=0.41  -24.55-10.14 | 65.77(30.37) | p=0.88  -24.97- 21.51 |
|  | AIEOP-BFM (N=9) | 63.89(28.26) |  | 68.33(24.15) |  | 69.38(18.27) |  | 78.33(27.55) |  | 67.50(33.21) |  |
| T5 | COG (N=25) | 63.00(27.75) | p=0.66  -24.72-15.87 | 75.89(26.77) | p=0.12  -28.88-4.48 | 62.05(19.94) | p=0.65  -16.73-10.47 | 77.08(26.41) | p=0.87  -19.45-16.48 | 62.80(26.88) | p=0.82  -20.72-16.56 |
|  | AIEOP-BFM (N=11) | 67.42(27.25) |  | 88.10(21.61) |  | 65.18(21.78) |  | 78.57(28.63) |  | 64.88(30.69) |  |
| T6 | COG (N=24) | 66.05(28.21) | p=0.77  -21.99-16.31 | 80.36(26.37) | p=0.87  -16.93-14.31 | 63.81(24.73) | p=0.58  -19.70-11.21 | 75.22.35) | p=0.57  -18.61-10.43 | 63.69(35.01) | p=0.95  -23.56-22.05 |
|  | AIEOP-BFM (N=11) | 68.89(31.57) |  | 81.67(19.21) |  | 68.06(21.80) |  | 79.44(22.02) |  | 64.44(35.84) |  |
| T7 | COG (N=21) | 61.94(33.66) | p=0.34  -28.77-10.07 | 84.94(21.33) | p=0.70  -10.85-15.93 | 64.17(25.39) | p=0.39  -20.89-8.23 | 76.15(26.61) | p=0.76  -14.14-19.22 | 68.95(28.59) | p=0.72  -17.21-24.59 |
|  | AIEOP-BFM (N=13) | 71.30(30.00) |  | 81.94(23.43) |  | 70.49(22.27) |  | 73.61(29.18) |  | 65.28(37.08) |  |
| T8 | COG (N=20) | 67.93(27.88) | p=0.40  -23.63-9.49 | 81.06(25.62) | p=0.71  -17.44-11.91 | 67.30(24.39) | p=0.63  -17.37-10.54 | 77.34(23.88) | p=0.74  -17.65-12.52 | 71.61(25.03) | p=0.91  -16.70-14.83 |
|  | AIEOP-BFM (N=12) | 75.00(27.00) |  | 83.82(21.95) |  | 70.71(20.76) |  | 79.90(27.01) |  | 72.54(28.07) |  |
| T9 | COG (N=19) | 66.98(28.54) | p=0.91  -19.29-17.31 | 81.41(23.84) | p=0.57  -19.26-10.78 | 64.50(22.16) | p=0.30  -20.97-6.69 | 72.44(24.01) | p=0.07  -29.67-1.41 | 64.10(31.33) | p=0.73  -24.04-17.06 |
|  | AIEOP-BFM (N=9) | 68.06(31.34) |  | 85.65(24.89) |  | 71.64(22.62) |  | 86.57(26.68) |  | 67.59(35.80) |  |
| T10 | COG (N=22) | 63.99(32.71) | p=0.23  -32.20-8.09 | 78.40(26.98) | p=0.11  -23.91-2.57 | 59.80(24.29) | p=0.03*  -11.19-21.29 | 75.00(23.80) | p=0.20  -23.83-5.08 | 68.83(27.11) | p=0.75  -22.08-15.99 |
|  | AIEOP-BFM (N=12) | 76.04(30.26) |  | 89.06(16.02) |  | 76.17(18.91) |  | 84.38(20.61) |  | 71.88(34.14) |  |
| T11 | COG (N=29) | 64.94(32.61) | p=0.27  -31.53-9.03 | 83.62(25.34) | p=0.53  -30.69- - 2.05 | 62.78(24.81) | p=0.27  -24.51-6.93 | 73.28(27.04) | p=0.35  -25.78-9.23 | 72.41(27.64) | p=0.56  -22.18-12.24 |
|  | AIEOP-BFM (N=14) | 76.19(26.72) |  | 78.57(23.27) |  | 71.58(21.89) |  | 81.55(25.77) |  | 77.39(22.75) |  |
| T12 | COG (N=27) | 67.33(29.16) | p=0.44  -27.45-12.11 | 79.33(27.23) | p=0.69  -13.21-19.65 | 60.42(21.67) | p=0.02*  -12.75-16.52 | 81.33(21.42) | p=0.79  -30.69- - 2.05 | 75.67(26.87) | p=0.75  -19.56-14.23 |
|  | AIEOP-BFM (N=13) | 75.00(31.18) |  | 76.11(20.14) |  | 75.97(16.87) |  | 79.44(23.33) |  | 78.33(23.10) |  |

SUPPLEMENTARY TABLE 5 COG vs. iBFM Protocol group parental emotional well-being comparisons (ET)

| **Time** | **Protocol Group** | **Distress (M(SD))** | **T-test**  **p and**  **95% CI** | **Anxiety (M(SD))** | **T-test**  **p and**  **95% CI** | **Depression (M(SD))** | **T-test**  **p and**  **95% CI** | **Anger (M(SD))** | **T-test**  **p and**  **95% CI** | **Need for Help (M(SD))** | **T-test**  **p and**  **95% CI** |
| --- | --- | --- | --- | --- | --- | --- | --- | --- | --- | --- | --- |
| T1 | COG | 4.59(2.48) | p=0.06  -4.95-0.12 | 5.76(2.51) | p=0.51  -3.42-1.75 | 2.94(2.33) | p=0.32  -3.83-1.32 | 3.65(2.76) | p=0.91  -2.97-2.66 | 3.38(2.80) | p=0.87  -3.10-2.65 |
|  | AIEOP-BFM | 7.00(2.00) |  | 6.60(2.07) |  | 4.20(2.78) |  | 3.80(2.17) |  | 3.60(2.19) |  |
| T2 | COG | 4.88(2.92) | p=0.33  -3.16-1.11 | 5.44(2.69) | p=0.13  -3.37-0.43 | 3.15(2.68) | p=0.05*  -4.07-0.01 | 4.00(2.82) | p=0.66  -2.55-1.65 | 3.31(2.70) | p=0.75  -2.38-1.72 |
|  | AIEOP-BFM | 5.91(2.95) |  | 6.91(2.30) |  | 5.18(3.06) |  | 4.45(3.01) |  | 3.64(3.08) |  |
| T3 | COG | 4.22(2.76) | p=0.17  -3.62-0.67 | 5.26(2.82) | p=0.12  -3.52-0.44 | 3.30(2.83) | p=0.29  -3.21-1.01 | 3.37(2.78) | p=0.94  -2.23-2.09 | 2.37(1.88) | p=0.05*  -3.29-0.03 |
|  | AIEOP-BFM | 5.70(3.09) |  | 6.80(1.99) |  | 4.40(2.72) |  | 3.44(2.74) |  | 4.00(2.74) |  |
| T4 | COG | 4.94(2.42) | p=0.25  -0.75-2.80 | 4.97(2.47) | p=0.85  -2.56-2.14 | 4.06(2.66) | p=0.73  -1.58-2.25 | 3.56(2.41) | p=0.26  -0.76-2.79 | 2.45(2.54) | p=0.99  -1.93-1.94 |
|  | AIEOP-BFM | 3.91(2.77) |  | 5.18(3.34) |  | 3.73(2.87) |  | 2.55(2.84) |  | 2.44(2.45) |  |
| T5 | COG | 4.55(3.14) | p=0.85  -1.85-2.23 | 4.87(3.12) | p=0.73  -2.31-1.63 | 3.26(2.91) | p=0.62  -1.41-2.36 | 3.87(2.89) | p=0.03*  0.22-3.24 | 2.42(2.28) | p=0.84  -1.73-1.42 |
|  | AIEOP-BFM | 4.36(3.15) |  | 5.21(2.83) |  | 2.79(2.89) |  | 2.14(1.99) |  | 2.57(2.74) |  |
| T6 | COG | 3.90(2.61) | p=0.55  -2.48-1.34 | 4.38(2.95) | p=0.39  -2.72-1.08 | 2.83(2.80) | p=0.97  -1.88-1.91 | 3.14(2.66) | p=0.76  -1.54-2.08 | 2.59(2.01) | p=0.55  -1.06-1.96 |
|  | AIEOP-BFM | 4.47(3.48) |  | 5.20(3.01) |  | 2.80(3.19) |  | 2.87(3.11) |  | 2.13(2.92) |  |
| T7 | COG | 3.75(2.94) | p=0.98  -1.80-1.75 | 4.63(2.99) | p=0.71  -2.04-1.40 | 3.09(3.10) | p=0.89  -1.96-1.71 | 3.59(2.97) | p=0.20  -0.61-2.79 | 1.60(2.25) | p=0.88  -1.48-1.26 |
|  | AIEOP-BFM | 3.78(3.10) |  | 4.94(2.75) |  | 3.22(3.08) |  | 2.50(2.68) |  | 1.71(2.20) |  |
| T8 | COG | 3.79(2.99) | p=0.23  -0.68-2.71 | 4.32(2.86) | p=0.41  -0.99-2.42 | 2.74(2.91) | p=0.47  -1.00-2.14 | 3.03(2.88) | p=0.02*  0.23-2.83 | 2.12(2.77) | p=0.35  -0.77-2.12 |
|  | AIEOP-BFM | 2.78(2.69) |  | 3.61(3.01) |  | 2.17(2.18) |  | 1.50(1.76) |  | 1.44(1.76) |  |
| T9 | COG | 3.35(2.70) | p=0.49  -1.07-2.20 | 4.62(3.01) | p=0.27  -0.76-2.66 | 3.35(2.79) | p=0.20  -0.52-2.43 | 3.31(2.49) | p=0.16  -0.43-2.49 | 1.76(2.55) | p=0.92  -1.45-1.60 |
|  | AIEOP-BFM | 2.78(2.56) |  | 3.67(2.35) |  | 2.39(2.06) |  | 2.28(2.14) |  | 1.69(1.99) |  |
| T10 | COG | 3.39(2.92) | p=0.04*  0.13-3.53 | 3.82(3.06) | p=0.24  -0.74-2.88 | 3.14(3.14) | p=0.09  -0.22-2.75 | 2.96(2.96) | p=0.17  -0.45-2.50 | 1.58(2.30) | p=0.92  -1.38-1.53 |
|  | AIEOP-BFM | 1.56(2.22) |  | 2.75(2.49) |  | 1.88(1.75) |  | 1.94(1.88) |  | 1.50(2.22) |  |
| T11 | COG | 3.18(2.65) | p=0.53  -1.10-2.13 | 3.79(2.82) | p=0.81  -1.97-1.54 | 2.46(2.70) | p=0.21  -0.52-2.25 | 3.21(2.69) | p=0.04*  0.99-2.99 | 1.85(2.45) | p=0.85  -1.69-1.40 |
|  | AIEOP-BFM | 2.67(2.16) |  | 4.00(2.54) |  | 1.60(1.77) |  | 1.67(1.95) |  | 2.00(2.23) |  |
| T12 | COG | 3.24(2.55) | p=0.49  -1.10-2.25 | 3.48(2.63) | p=0.54  -2.48-1.31 | 2.08(2.45) | p=0.58  -0.90-1.59 | 2.20(2.45) | p=0.67  -1.21-1.88 | 1.75(2.56) | p=0.60  -2.18-1.28 |
|  | AIEOP-BFM | 2.67(2.50) |  | 4.07(3.24) |  | 1.73(1.43) |  | 1.87(2.13) |  | 2.20(2.65) |  |

*Significant difference (p<0.05)
