# Supplementary material for: Prospective longitudinal evaluation of treatment-related toxicity and health-related quality of life during the first year of treatment for pediatric acute lymphoblastic leukemia
Source: BMC Cancer. 2022 Sep 15;22:985. doi: 10.1186/s12885-022-10072-x (PMC9479356; doi:10.1186/s12885-022-10072-x)
Supplement: Supplementary file 2 — Additional file 2: Data Table 1. HUI3 Data. Data Table 2. PedsQL Cancer Data. Data Table 3. Emotion Thermometer Data. [file 12885_2022_10072_MOESM2_ESM.docx]

Data Table 1. HUI3 Data

| **ID** | **Protocol** | **Months**  **Post-Diagnosis** | **Utility**  **Score** | **Vision** | **Hearing** | **Speech** | **Ambulation** | **Dexterity** | **Emotion** | **Cognition** | **Pain** |
| --- | --- | --- | --- | --- | --- | --- | --- | --- | --- | --- | --- |
| 0013-0001 | iBFM | 7.00 | 1.00 | 1.00 | 1.00 | 1.00 | 1.00 | 1.00 | 1.00 | 1.00 | 1.00 |
| 0013-0001 | iBFM | 8.00 | 0.79 | 1.00 | 1.00 | 1.00 | 0.93 | 1.00 | 0.95 | 1.00 | 0.96 |
| 0013-0001 | iBFM | 9.00 | 0.85 | 1.00 | 1.00 | 1.00 | 0.93 | 1.00 | 1.00 | 1.00 | 0.96 |
| 0013-0001 | iBFM | 10.00 | 0.95 | 1.00 | 1.00 | 1.00 | 1.00 | 1.00 | 1.00 | 1.00 | 0.96 |
| 0013-0001 | iBFM | 11.00 | 1.00 | 1.00 | 1.00 | 1.00 | 1.00 | 1.00 | 1.00 | 1.00 | 1.00 |
| 0013-0001 | iBFM | 12.00 | 0.95 | 1.00 | 1.00 | 1.00 | 1.00 | 1.00 | 1.00 | 1.00 | 0.96 |
| 0013-0003 | iBFM | 4.00 | 0.80 | 1.00 | 1.00 | 0.89 | 1.00 | 1.00 | 1.00 | 1.00 | 0.96 |
| 0013-0003 | iBFM | 5.00 | 0.87 | 1.00 | 1.00 | 0.94 | 1.00 | 1.00 | 1.00 | 1.00 | 0.96 |
| 0013-0003 | iBFM | 6.00 | 0.00 | 1.00 | 1.00 | 0.94 | 0.65 | 1.00 | 0.46 | 1.00 | 0.96 |
| 0013-0003 | iBFM | 7.00 | 1.00 | 1.00 | 1.00 | 1.00 | 1.00 | 1.00 | 1.00 | 1.00 | 1.00 |
| 0013-0003 | iBFM | 8.00 | 0.22 | 1.00 | 1.00 | 0.94 | 0.93 | 1.00 | 0.64 | 1.00 | 0.77 |
| 0013-0003 | iBFM | 9.00 | 0.92 | 1.00 | 1.00 | 0.94 | 1.00 | 1.00 | 1.00 | 1.00 | 1.00 |
| 0013-0003 | iBFM | 10.00 | 0.92 | 1.00 | 1.00 | 0.94 | 1.00 | 1.00 | 1.00 | 1.00 | 1.00 |
| 0013-0003 | iBFM | 11.00 | -0.08 | 1.00 | 1.00 | 0.94 | 0.65 | 1.00 | 0.64 | 1.00 | 0.55 |
| 0013-0003 | iBFM | 12.00 | 0.92 | 1.00 | 1.00 | 0.94 | 1.00 | 1.00 | 1.00 | 1.00 | 1.00 |
| 0013-0004 | iBFM | 4.00 | 0.73 | 1.00 | 1.00 | 0.94 | 1.00 | 1.00 | 0.95 | 1.00 | 0.90 |
| 0013-0004 | iBFM | 5.00 | 0.75 | 1.00 | 1.00 | 0.89 | 1.00 | 1.00 | 1.00 | 0.92 | 1.00 |
| 0013-0004 | iBFM | 6.00 | 0.37 | 1.00 | 1.00 | 0.94 | 0.73 | 1.00 | 0.95 | 0.92 | 0.90 |
| 0013-0004 | iBFM | 7.00 | 0.33 | 1.00 | 1.00 | 1.00 | 0.73 | 1.00 | 0.85 | 0.92 | 0.90 |
| 0013-0004 | iBFM | 8.00 | 0.92 | 1.00 | 1.00 | 0.94 | 1.00 | 1.00 | 1.00 | 1.00 | 1.00 |
| 0013-0004 | iBFM | 10.00 | 0.83 | 1.00 | 1.00 | 0.94 | 0.93 | 1.00 | 1.00 | 1.00 | 1.00 |
| 0013-0004 | iBFM | 11.00 | 0.73 | 1.00 | 1.00 | 0.94 | 0.93 | 1.00 | 1.00 | 0.92 | 1.00 |
| 0013-0004 | iBFM | 12.00 | 0.92 | 1.00 | 1.00 | 0.94 | 1.00 | 1.00 | 1.00 | 1.00 | 1.00 |
| 0013-0005 | iBFM | 3.00 | 0.41 | 1.00 | 1.00 | 0.94 | 0.73 | 1.00 | 1.00 | 0.92 | 0.90 |
| 0013-0005 | iBFM | 5.00 | 0.89 | 1.00 | 1.00 | 1.00 | 1.00 | 1.00 | 1.00 | 0.92 | 1.00 |
| 0013-0005 | iBFM | 6.00 | 0.43 | 1.00 | 1.00 | 0.94 | 0.73 | 1.00 | 0.95 | 1.00 | 0.90 |
| 0013-0005 | iBFM | 7.00 | 0.84 | 1.00 | 1.00 | 1.00 | 1.00 | 1.00 | 1.00 | 0.92 | 0.96 |
| 0013-0005 | iBFM | 9.00 | 1.00 | 1.00 | 1.00 | 1.00 | 1.00 | 1.00 | 1.00 | 1.00 | 1.00 |
| 0013-0005 | iBFM | 10.00 | 1.00 | 1.00 | 1.00 | 1.00 | 1.00 | 1.00 | 1.00 | 1.00 | 1.00 |
| 0013-0006 | iBFM | 4.00 | 0.30 | 1.00 | 1.00 | 1.00 | 0.73 | 1.00 | 0.95 | 0.92 | 0.77 |
| 0013-0006 | iBFM | 5.00 | 0.71 | 1.00 | 1.00 | 1.00 | 1.00 | 1.00 | 0.95 | 0.92 | 0.90 |
| 0013-0006 | iBFM | 7.00 | 0.80 | 1.00 | 1.00 | 1.00 | 1.00 | 1.00 | 0.95 | 1.00 | 0.90 |

Note: 999.00=missing data

Data Table 1. HUI3 Data

| **ID** | **Protocol** | **Months**  **Post-Diagnosis** | **Utility**  **Score** | **Vision** | **Hearing** | **Speech** | **Ambulation** | **Dexterity** | **Emotion** | **Cognition** | **Pain** |
| --- | --- | --- | --- | --- | --- | --- | --- | --- | --- | --- | --- |
| 0013-0006 | iBFM | 8.00 | 0.41 | 1.00 | 1.00 | 0.94 | 0.86 | 1.00 | 0.85 | 0.92 | 0.90 |
| 0013-0006 | iBFM | 9.00 | 0.73 | 1.00 | 1.00 | 0.94 | 1.00 | 1.00 | 0.95 | 1.00 | 0.90 |
| 0013-0006 | iBFM | 11.00 | 0.76 | 1.00 | 1.00 | 1.00 | 1.00 | 1.00 | 1.00 | 0.92 | 0.90 |
| 0013-0006 | iBFM | 12.00 | 0.76 | 1.00 | 1.00 | 0.94 | 1.00 | 1.00 | 0.95 | 0.92 | 1.00 |
| 0013-0008 | iBFM | 5.00 | 0.95 | 1.00 | 1.00 | 1.00 | 1.00 | 1.00 | 1.00 | 1.00 | 0.96 |
| 0013-0008 | iBFM | 6.00 | 0.95 | 1.00 | 1.00 | 1.00 | 1.00 | 1.00 | 1.00 | 1.00 | 0.96 |
| 0013-0008 | iBFM | 7.00 | 0.95 | 1.00 | 1.00 | 1.00 | 1.00 | 1.00 | 1.00 | 1.00 | 0.96 |
| 0013-0008 | iBFM | 8.00 | 1.00 | 1.00 | 1.00 | 1.00 | 1.00 | 1.00 | 1.00 | 1.00 | 1.00 |
| 0013-0008 | iBFM | 9.00 | 0.95 | 1.00 | 1.00 | 1.00 | 1.00 | 1.00 | 1.00 | 1.00 | 0.96 |
| 0013-0008 | iBFM | 10.00 | 1.00 | 1.00 | 1.00 | 1.00 | 1.00 | 1.00 | 1.00 | 1.00 | 1.00 |
| 0013-0008 | iBFM | 11.00 | 0.95 | 1.00 | 1.00 | 1.00 | 1.00 | 1.00 | 1.00 | 1.00 | 0.96 |
| 0013-0008 | iBFM | 12.00 | 1.00 | 1.00 | 1.00 | 1.00 | 1.00 | 1.00 | 1.00 | 1.00 | 1.00 |
| 0014-0024 | COG | 4.00 | 0.85 | 1.00 | 1.00 | 1.00 | 0.93 | 1.00 | 1.00 | 1.00 | 0.96 |
| 0014-0024 | COG | 5.00 | 0.11 | 1.00 | 1.00 | 1.00 | 0.93 | 1.00 | 1.00 | 0.42 | 0.90 |
| 0014-0024 | COG | 6.00 | 0.56 | 1.00 | 1.00 | 1.00 | 0.93 | 1.00 | 0.95 | 1.00 | 0.77 |
| 0014-0024 | COG | 7.00 | 0.33 | 1.00 | 1.00 | 1.00 | 0.73 | 1.00 | 0.85 | 0.92 | 0.90 |
| 0014-0024 | COG | 8.00 | 0.33 | 1.00 | 1.00 | 1.00 | 0.73 | 1.00 | 0.85 | 0.92 | 0.90 |
| 0014-0024 | COG | 10.00 | 0.06 | 1.00 | 1.00 | 1.00 | 0.73 | 1.00 | 0.85 | 0.92 | 0.55 |
| 0014-0024 | COG | 11.00 | 0.28 | 1.00 | 1.00 | 1.00 | 0.73 | 1.00 | 0.85 | 1.00 | 0.77 |
| 0014-0024 | COG | 12.00 | 0.63 | 1.00 | 1.00 | 1.00 | 0.93 | 1.00 | 0.95 | 0.92 | 0.90 |
| 0014-0025 | COG | 1.00 | 0.29 | 1.00 | 1.00 | 0.89 | 0.65 | 1.00 | 0.95 | 0.92 | 0.96 |
| 0014-0025 | COG | 2.00 | 0.34 | 1.00 | 1.00 | 0.95 | 0.73 | 0.95 | 0.95 | 0.92 | 0.90 |
| 0014-0025 | COG | 4.00 | 0.29 | 1.00 | 1.00 | 0.94 | 0.73 | 1.00 | 0.85 | 0.92 | 0.90 |
| 0014-0025 | COG | 5.00 | 0.20 | 1.00 | 1.00 | 0.94 | 0.73 | 1.00 | 0.85 | 0.92 | 0.77 |
| 0015-0011 | COG | 5.00 | 0.95 | 1.00 | 1.00 | 1.00 | 1.00 | 1.00 | 1.00 | 1.00 | 0.96 |
| 0015-0011 | COG | 7.00 | 0.95 | 1.00 | 1.00 | 1.00 | 1.00 | 1.00 | 1.00 | 1.00 | 0.96 |
| 0015-0011 | COG | 8.00 | 0.88 | 1.00 | 0.95 | 1.00 | 1.00 | 1.00 | 1.00 | 1.00 | 0.96 |
| 0015-0011 | COG | 9.00 | 0.95 | 1.00 | 1.00 | 1.00 | 1.00 | 1.00 | 1.00 | 1.00 | 0.96 |

Note: 999.00=missing data

Data Table 1. HUI3 Data

| **ID** | **Protocol** | **Months Post-Diagnosis** | **Utility Score** | **Vision** | **Hearing** | **Speech** | **Ambulation** | **Dexterity** | **Emotion** | **Cognition** | **Pain** |
| --- | --- | --- | --- | --- | --- | --- | --- | --- | --- | --- | --- |
| 0015-0012 | COG | 6.00 | 0.80 | 1.00 | 1.00 | 1.00 | 1.00 | 1.00 | 0.95 | 1.00 | 0.90 |
| 0015-0012 | COG | 8.00 | 0.80 | 1.00 | 1.00 | 1.00 | 1.00 | 1.00 | 0.95 | 1.00 | 0.90 |
| 0015-0012 | COG | 11.00 | 0.84 | 1.00 | 1.00 | 1.00 | 1.00 | 1.00 | 1.00 | 0.92 | 0.96 |
| 0015-0012 | COG | 12.00 | 0.88 | 1.00 | 1.00 | 1.00 | 1.00 | 1.00 | 0.95 | 1.00 | 0.96 |
| 0015-0018 | COG | 3.00 | 0.95 | 1.00 | 1.00 | 1.00 | 1.00 | 1.00 | 1.00 | 1.00 | 0.96 |
| 0015-0018 | COG | 5.00 | 0.92 | 1.00 | 1.00 | 0.94 | 1.00 | 1.00 | 1.00 | 1.00 | 1.00 |
| 0015-0018 | COG | 6.00 | 0.92 | 1.00 | 1.00 | 0.94 | 1.00 | 1.00 | 1.00 | 1.00 | 1.00 |
| 0015-0018 | COG | 7.00 | 0.88 | 1.00 | 1.00 | 1.00 | 1.00 | 1.00 | 0.95 | 1.00 | 0.96 |
| 0015-0018 | COG | 9.00 | 0.77 | 1.00 | 1.00 | 0.94 | 1.00 | 1.00 | 1.00 | 0.92 | 0.96 |
| 0015-0018 | COG | 10.00 | 0.65 | 1.00 | 1.00 | 0.89 | 1.00 | 1.00 | 0.95 | 0.92 | 0.96 |
| 0015-0018 | COG | 11.00 | 0.77 | 1.00 | 1.00 | 0.94 | 0.93 | 1.00 | 0.95 | 1.00 | 1.00 |
| 0015-0018 | COG | 12.00 | 0.69 | 1.00 | 1.00 | 0.94 | 0.93 | 1.00 | 1.00 | 0.92 | 0.96 |
| 0015-0019 | COG | 2.00 | 0.79 | 1.00 | 1.00 | 1.00 | 0.93 | 1.00 | 0.95 | 1.00 | 0.96 |
| 0015-0019 | COG | 3.00 | 0.95 | 1.00 | 1.00 | 1.00 | 1.00 | 1.00 | 1.00 | 1.00 | 0.96 |
| 0015-0019 | COG | 4.00 | 0.84 | 1.00 | 1.00 | 1.00 | 1.00 | 1.00 | 1.00 | 0.92 | 0.96 |
| 0015-0019 | COG | 5.00 | 0.65 | 1.00 | 1.00 | 1.00 | 1.00 | 1.00 | 1.00 | 0.83 | 0.90 |
| 0015-0019 | COG | 6.00 | 0.78 | 1.00 | 1.00 | 1.00 | 1.00 | 1.00 | 0.95 | 0.92 | 0.96 |
| 0015-0019 | COG | 8.00 | 0.88 | 1.00 | 1.00 | 1.00 | 1.00 | 1.00 | 0.95 | 1.00 | 0.96 |
| 0015-0019 | COG | 9.00 | 0.95 | 1.00 | 1.00 | 1.00 | 1.00 | 1.00 | 1.00 | 1.00 | 0.96 |
| 0015-0019 | COG | 10.00 | 0.95 | 1.00 | 1.00 | 1.00 | 1.00 | 1.00 | 1.00 | 1.00 | 0.96 |
| 0015-0019 | COG | 11.00 | 0.95 | 1.00 | 1.00 | 1.00 | 1.00 | 1.00 | 1.00 | 1.00 | 0.96 |
| 0015-0019 | COG | 12.00 | 0.84 | 1.00 | 1.00 | 1.00 | 1.00 | 1.00 | 1.00 | 0.92 | 0.96 |
| 0015-0020 | COG | 2.00 | 0.90 | 1.00 | 1.00 | 1.00 | 0.93 | 1.00 | 1.00 | 1.00 | 1.00 |
| 0015-0020 | COG | 3.00 | 0.87 | 1.00 | 1.00 | 0.94 | 1.00 | 1.00 | 1.00 | 1.00 | 0.96 |
| 0015-0020 | COG | 7.00 | 0.87 | 1.00 | 1.00 | 0.94 | 1.00 | 1.00 | 1.00 | 1.00 | 0.96 |
| 0015-0020 | COG | 8.00 | 1.00 | 1.00 | 1.00 | 1.00 | 1.00 | 1.00 | 1.00 | 1.00 | 1.00 |
| 0015-0020 | COG | 9.00 | 0.95 | 1.00 | 1.00 | 1.00 | 1.00 | 1.00 | 1.00 | 1.00 | 0.96 |
| 0015-0020 | COG | 11.00 | 0.93 | 1.00 | 1.00 | 1.00 | 1.00 | 1.00 | 0.95 | 1.00 | 1.00 |
| 0015-0021 | COG | 3.00 | 1.00 | 1.00 | 1.00 | 1.00 | 1.00 | 1.00 | 1.00 | 1.00 | 1.00 |
| 0015-0021 | COG | 4.00 | 1.00 | 1.00 | 1.00 | 1.00 | 1.00 | 1.00 | 1.00 | 1.00 | 1.00 |
| 0015-0021 | COG | 5.00 | 1.00 | 1.00 | 1.00 | 1.00 | 1.00 | 1.00 | 1.00 | 1.00 | 1.00 |
| 0015-0021 | COG | 6.00 | 0.95 | 1.00 | 1.00 | 1.00 | 1.00 | 1.00 | 1.00 | 1.00 | 0.96 |

Note: 999.00=missing data

Data Table 1. HUI3 Data

| **ID** | **Protocol** | **Months Post-Diagnosis** | **Utility Score** | **Vision** | **Hearing** | **Speech** | **Ambulation** | **Dexterity** | **Emotion** | **Cognition** | **Pain** |
| --- | --- | --- | --- | --- | --- | --- | --- | --- | --- | --- | --- |
| 0015-0021 | COG | 7.00 | 1.00 | 1.00 | 1.00 | 1.00 | 1.00 | 1.00 | 1.00 | 1.00 | 1.00 |
| 0015-0021 | COG | 8.00 | 1.00 | 1.00 | 1.00 | 1.00 | 1.00 | 1.00 | 1.00 | 1.00 | 1.00 |
| 0015-0022 | COG | 2.00 | 0.93 | 1.00 | 1.00 | 1.00 | 1.00 | 1.00 | 0.95 | 1.00 | 1.00 |
| 0015-0022 | COG | 3.00 | 1.00 | 1.00 | 1.00 | 1.00 | 1.00 | 1.00 | 1.00 | 1.00 | 1.00 |
| 0015-0022 | COG | 4.00 | 1.00 | 1.00 | 1.00 | 1.00 | 1.00 | 1.00 | 1.00 | 1.00 | 1.00 |
| 0015-0022 | COG | 6.00 | 1.00 | 1.00 | 1.00 | 1.00 | 1.00 | 1.00 | 1.00 | 1.00 | 1.00 |
| 0016-0005 | iBFM | 8.00 | 1.00 | 1.00 | 1.00 | 1.00 | 1.00 | 1.00 | 1.00 | 1.00 | 1.00 |
| 0016-0005 | iBFM | 9.00 | 1.00 | 1.00 | 1.00 | 1.00 | 1.00 | 1.00 | 1.00 | 1.00 | 1.00 |
| 0016-0005 | iBFM | 10.00 | 0.90 | 1.00 | 1.00 | 1.00 | 0.93 | 1.00 | 1.00 | 1.00 | 1.00 |
| 0016-0005 | iBFM | 11.00 | 1.00 | 1.00 | 1.00 | 1.00 | 1.00 | 1.00 | 1.00 | 1.00 | 1.00 |
| 0016-0005 | iBFM | 12.00 | 1.00 | 1.00 | 1.00 | 1.00 | 1.00 | 1.00 | 1.00 | 1.00 | 1.00 |
| 0016-0008 | iBFM | 2.00 | 0.59 | 1.00 | 1.00 | 1.00 | 0.93 | 1.00 | 1.00 | 1.00 | 0.90 |
| 0016-0008 | iBFM | 3.00 | 1.00 | 1.00 | 1.00 | 1.00 | 1.00 | 1.00 | 1.00 | 1.00 | 1.00 |
| 0016-0008 | iBFM | 4.00 | 0.95 | 1.00 | 1.00 | 1.00 | 1.00 | 1.00 | 1.00 | 1.00 | 0.96 |
| 0016-0008 | iBFM | 5.00 | 0.89 | 1.00 | 1.00 | 1.00 | 1.00 | 1.00 | 1.00 | 0.92 | 1.00 |
| 0016-0008 | iBFM | 8.00 | 0.83 | 1.00 | 1.00 | 1.00 | 1.00 | 1.00 | 0.95 | 0.92 | 1.00 |
| 0016-0010 | iBFM | 2.00 | 1.00 | 1.00 | 1.00 | 1.00 | 1.00 | 1.00 | 1.00 | 1.00 | 1.00 |
| 0016-0010 | iBFM | 4.00 | 1.00 | 1.00 | 1.00 | 1.00 | 1.00 | 1.00 | 1.00 | 1.00 | 1.00 |
| 0016-0010 | iBFM | 5.00 | 1.00 | 1.00 | 1.00 | 1.00 | 1.00 | 1.00 | 1.00 | 1.00 | 1.00 |
| 0016-0010 | iBFM | 6.00 | 1.00 | 1.00 | 1.00 | 1.00 | 1.00 | 1.00 | 1.00 | 1.00 | 1.00 |
| 0016-0010 | iBFM | 7.00 | 1.00 | 1.00 | 1.00 | 1.00 | 1.00 | 1.00 | 1.00 | 1.00 | 1.00 |
| 0016-0026 | iBFM | 4.00 | 0.33 | 1.00 | 1.00 | 0.94 | 0.73 | 0.95 | 0.95 | 0.92 | 0.90 |

Note: 999.00=missing data

Data Table 1. HUI3 Data

| **ID** | **Protocol** | **Months Post-Diagnosis** | **Utility Score** | **Vision** | **Hearing** | **Speech** | **Ambulation** | **Dexterity** | **Emotion** | **Cognition** | **Pain** |
| --- | --- | --- | --- | --- | --- | --- | --- | --- | --- | --- | --- |
| 0016-0026 | iBFM | 5.00 | 0.57 | 1.00 | 1.00 | 0.81 | 0.93 | 0.95 | 1.00 | 1.00 | 0.96 |
| 0016-0026 | iBFM | 6.00 | 0.78 | 1.00 | 1.00 | 0.94 | 0.93 | 1.00 | 1.00 | 1.00 | 0.96 |
| 0016-0026 | iBFM | 7.00 | 0.34 | 1.00 | 1.00 | 0.89 | 0.73 | 0.95 | 0.95 | 0.92 | 0.96 |
| 0016-0026 | iBFM | 8.00 | 0.41 | 1.00 | 1.00 | 0.89 | 0.86 | 1.00 | 1.00 | 0.83 | 0.90 |
| 0016-0026 | iBFM | 9.00 | 0.63 | 1.00 | 1.00 | 0.89 | 0.93 | 1.00 | 1.00 | 0.92 | 0.96 |
| 0016-0026 | iBFM | 10.00 | 0.58 | 1.00 | 0.95 | 0.89 | 0.93 | 1.00 | 1.00 | 0.92 | 0.96 |
| 0016-0026 | iBFM | 11.00 | 0.69 | 1.00 | 1.00 | 0.94 | 0.93 | 1.00 | 1.00 | 0.92 | 0.96 |
| 0016-0026 | iBFM | 12.00 | 0.66 | 1.00 | 1.00 | 0.89 | 0.93 | 0.95 | 1.00 | 1.00 | 0.96 |
| 0016-0032 | iBFM | 2.00 | 0.52 | 1.00 | 1.00 | 0.94 | 0.73 | 1.00 | 0.95 | 1.00 | 1.00 |
| 0016-0032 | iBFM | 3.00 | 0.83 | 1.00 | 1.00 | 0.94 | 0.93 | 1.00 | 1.00 | 1.00 | 1.00 |
| 0016-0032 | iBFM | 5.00 | 0.85 | 1.00 | 1.00 | 0.94 | 1.00 | 1.00 | 0.95 | 1.00 | 1.00 |
| 0016-0032 | iBFM | 6.00 | 0.92 | 1.00 | 1.00 | 0.94 | 1.00 | 1.00 | 1.00 | 1.00 | 1.00 |
| 0016-0032 | iBFM | 7.00 | 0.77 | 1.00 | 1.00 | 0.94 | 0.93 | 1.00 | 0.95 | 1.00 | 1.00 |
| 0016-0032 | iBFM | 8.00 | 0.83 | 1.00 | 1.00 | 0.94 | 0.93 | 1.00 | 1.00 | 1.00 | 1.00 |
| 0016-0032 | iBFM | 9.00 | 0.92 | 1.00 | 1.00 | 0.94 | 1.00 | 1.00 | 1.00 | 1.00 | 1.00 |
| 0016-0032 | iBFM | 10.00 | 0.85 | 1.00 | 1.00 | 0.89 | 1.00 | 1.00 | 1.00 | 1.00 | 1.00 |
| 0016-0032 | iBFM | 12.00 | 0.92 | 1.00 | 1.00 | 0.94 | 1.00 | 1.00 | 1.00 | 1.00 | 1.00 |
| 0016-0033 | iBFM | 2.00 | 0.48 | 1.00 | 1.00 | 1.00 | 0.73 | 1.00 | 0.95 | 1.00 | 0.90 |
| 0016-0033 | iBFM | 3.00 | 0.53 | 1.00 | 1.00 | 1.00 | 0.73 | 1.00 | 1.00 | 1.00 | 0.90 |
| 0016-0033 | iBFM | 6.00 | 0.32 | 1.00 | 1.00 | 0.94 | 0.73 | 1.00 | 0.95 | 1.00 | 0.77 |
| 0016-0033 | iBFM | 8.00 | 0.53 | 1.00 | 1.00 | 0.94 | 0.73 | 1.00 | 1.00 | 1.00 | 0.96 |
| 0016-0033 | iBFM | 9.00 | 0.71 | 1.00 | 1.00 | 0.94 | 0.93 | 1.00 | 1.00 | 1.00 | 0.90 |
| 0016-0033 | iBFM | 10.00 | 0.92 | 1.00 | 1.00 | 0.94 | 1.00 | 1.00 | 1.00 | 1.00 | 1.00 |
| 0016-0033 | iBFM | 11.00 | 0.71 | 1.00 | 1.00 | 0.94 | 0.93 | 1.00 | 1.00 | 1.00 | 0.90 |
| 0016-0033 | iBFM | 12.00 | 0.87 | 1.00 | 1.00 | 0.94 | 1.00 | 1.00 | 1.00 | 1.00 | 0.96 |
| 0016-0034 | iBFM | 2.00 | 0.48 | 1.00 | 1.00 | 1.00 | 0.65 | 1.00 | 1.00 | 1.00 | 0.96 |
| 0016-0034 | iBFM | 6.00 | 1.00 | 1.00 | 1.00 | 1.00 | 1.00 | 1.00 | 1.00 | 1.00 | 1.00 |
| 0016-0034 | iBFM | 7.00 | 0.80 | 1.00 | 1.00 | 1.00 | 1.00 | 1.00 | 0.95 | 1.00 | 0.90 |
| 0016-0034 | iBFM | 9.00 | 0.95 | 1.00 | 1.00 | 1.00 | 1.00 | 1.00 | 1.00 | 1.00 | 0.96 |
| 0016-0035 | iBFM | 1.00 | 0.26 | 1.00 | 1.00 | 1.00 | 0.65 | 1.00 | 1.00 | 0.92 | 0.77 |
| 0016-0035 | iBFM | 3.00 | 0.25 | 1.00 | 1.00 | 1.00 | 0.65 | 0.95 | 0.95 | 1.00 | 0.77 |

Note: 999.00=missing data

Data Table 1. HUI3 Data

| **ID** | **Protocol** | **Months Post-Diagnosis** | **Utility Score** | **Vision** | **Hearing** | **Speech** | **Ambulation** | **Dexterity** | **Emotion** | **Cognition** | **Pain** |
| --- | --- | --- | --- | --- | --- | --- | --- | --- | --- | --- | --- |
| 0016-0037 | iBFM | 1.00 | 0.65 | 1.00 | 1.00 | 0.89 | 1.00 | 1.00 | 0.95 | 0.92 | 0.96 |
| 0016-0038 | iBFM | 1.00 | 0.60 | 1.00 | 1.00 | 1.00 | 0.93 | 1.00 | 0.85 | 1.00 | 0.90 |
| 0016-0043 | iBFM | 2.00 | 0.53 | 1.00 | 1.00 | 0.89 | 0.93 | 1.00 | 1.00 | 0.83 | 0.96 |
| 0016-0043 | iBFM | 6.00 | 0.71 | 1.00 | 1.00 | 0.89 | 1.00 | 1.00 | 1.00 | 0.92 | 0.96 |
| 0016-0043 | iBFM | 7.00 | 0.66 | 1.00 | 1.00 | 0.94 | 1.00 | 1.00 | 1.00 | 0.83 | 0.96 |
| 0016-0043 | iBFM | 8.00 | 0.65 | 1.00 | 1.00 | 0.89 | 1.00 | 1.00 | 0.95 | 0.92 | 0.96 |
| 0016-0043 | iBFM | 9.00 | 0.81 | 1.00 | 1.00 | 0.94 | 1.00 | 1.00 | 1.00 | 0.92 | 1.00 |
| 0016-0043 | iBFM | 11.00 | 0.75 | 1.00 | 1.00 | 0.89 | 1.00 | 1.00 | 1.00 | 0.92 | 1.00 |
| 0016-0043 | iBFM | 12.00 | 1.00 | 1.00 | 1.00 | 1.00 | 1.00 | 1.00 | 1.00 | 1.00 | 1.00 |
| 0016-0045 | iBFM | 3.00 | 0.85 | 1.00 | 1.00 | 1.00 | 0.93 | 1.00 | 1.00 | 1.00 | 0.96 |
| 0016-0045 | iBFM | 4.00 | 0.85 | 1.00 | 1.00 | 1.00 | 0.93 | 1.00 | 1.00 | 1.00 | 0.96 |
| 0016-0045 | iBFM | 5.00 | 1.00 | 1.00 | 1.00 | 1.00 | 1.00 | 1.00 | 1.00 | 1.00 | 1.00 |
| 0016-0045 | iBFM | 6.00 | 0.72 | 1.00 | 1.00 | 1.00 | 0.93 | 1.00 | 0.95 | 1.00 | 0.90 |
| 0016-0045 | iBFM | 8.00 | 0.76 | 1.00 | 1.00 | 1.00 | 0.93 | 1.00 | 1.00 | 0.92 | 0.96 |
| 0016-0045 | iBFM | 9.00 | 1.00 | 1.00 | 1.00 | 1.00 | 1.00 | 1.00 | 1.00 | 1.00 | 1.00 |
| 0016-0045 | iBFM | 10.00 | 0.90 | 1.00 | 1.00 | 1.00 | 0.93 | 1.00 | 1.00 | 1.00 | 1.00 |
| 0016-0045 | iBFM | 11.00 | 1.00 | 1.00 | 1.00 | 1.00 | 1.00 | 1.00 | 1.00 | 1.00 | 1.00 |
| 0016-0045 | iBFM | 12.00 | 1.00 | 1.00 | 1.00 | 1.00 | 1.00 | 1.00 | 1.00 | 1.00 | 1.00 |
| 0016-0046 | iBFM | 5.00 | 0.95 | 1.00 | 1.00 | 1.00 | 1.00 | 1.00 | 1.00 | 1.00 | 0.96 |
| 0016-0046 | iBFM | 6.00 | 0.44 | 1.00 | 1.00 | 1.00 | 0.73 | 1.00 | 0.95 | 0.95 | 0.90 |
| 0016-0046 | iBFM | 7.00 | 0.55 | 1.00 | 1.00 | 1.00 | 0.73 | 1.00 | 1.00 | 0.92 | 1.00 |
| 0016-0046 | iBFM | 8.00 | 0.89 | 1.00 | 1.00 | 1.00 | 1.00 | 1.00 | 1.00 | 0.92 | 1.00 |
| 0016-0046 | iBFM | 9.00 | 1.00 | 1.00 | 1.00 | 1.00 | 1.00 | 1.00 | 1.00 | 1.00 | 1.00 |
| 0016-0046 | iBFM | 10.00 | 1.00 | 1.00 | 1.00 | 1.00 | 1.00 | 1.00 | 1.00 | 1.00 | 1.00 |
| 0016-0046 | iBFM | 11.00 | 0.89 | 1.00 | 1.00 | 1.00 | 1.00 | 1.00 | 1.00 | 0.92 | 1.00 |
| 0016-0047 | iBFM | 4.00 | 1.00 | 1.00 | 1.00 | 1.00 | 1.00 | 1.00 | 1.00 | 1.00 | 1.00 |
| 0016-0047 | iBFM | 6.00 | 0.93 | 1.00 | 1.00 | 1.00 | 1.00 | 1.00 | 0.95 | 1.00 | 1.00 |
| 0016-0047 | iBFM | 7.00 | 0.59 | 1.00 | 1.00 | 1.00 | 0.73 | 1.00 | 1.00 | 1.00 | 0.96 |
| 0016-0047 | iBFM | 8.00 | 0.93 | 1.00 | 1.00 | 1.00 | 1.00 | 1.00 | 1.00 | 0.95 | 1.00 |
| 0016-0047 | iBFM | 9.00 | 0.93 | 1.00 | 1.00 | 1.00 | 1.00 | 1.00 | 1.00 | 0.95 | 1.00 |

Note: 999.00=missing data

Data Table 1. HUI3 Data

| **ID** | **Protocol** | **Months Post-Diagnosis** | **Utility Score** | **Vision** | **Hearing** | **Speech** | **Ambulation** | **Dexterity** | **Emotion** | **Cognition** | **Pain** |
| --- | --- | --- | --- | --- | --- | --- | --- | --- | --- | --- | --- |
| 0016-0047 | iBFM | 10.00 | 0.84 | 1.0000 | 1.00 | 1.00 | 1.00 | 1.00 | 1.00 | 0.92 | 0.96 |
| 0016-0047 | iBFM | 11.00 | 0.88 | 1.0000 | 1.00 | 1.00 | 1.00 | 1.00 | 0.95 | 1.00 | 0.96 |
| 0016-0047 | iBFM | 12.00 | 1.00 | 1.0000 | 1.00 | 1.00 | 1.00 | 1.00 | 1.00 | 1.00 | 1.00 |
| 0016-0048 | iBFM | 7.00 | 1.00 | 1.0000 | 1.00 | 1.00 | 1.00 | 1.00 | 1.00 | 1.00 | 1.00 |
| 0016-0048 | iBFM | 8.00 | 0.90 | 1.0000 | 1.00 | 1.00 | 0.93 | 1.00 | 1.00 | 1.00 | 1.00 |
| 0016-0048 | iBFM | 9.00 | 1.00 | 1.0000 | 1.00 | 1.00 | 1.00 | 1.00 | 1.00 | 1.00 | 1.00 |
| 0016-0048 | iBFM | 10.00 | 1.00 | 1.0000 | 1.00 | 1.00 | 1.00 | 1.00 | 1.00 | 1.00 | 1.00 |
| 0016-0048 | iBFM | 11.00 | 0.85 | 1.0000 | 1.00 | 1.00 | 0.93 | 1.00 | 1.00 | 1.00 | 0.96 |
| 0016-0048 | iBFM | 12.00 | 0.72 | 1.0000 | 1.00 | 1.00 | 0.93 | 1.00 | 0.95 | 1.00 | 0.90 |
| 0016-0051 | iBFM | 1.00 | 0.45 | 1.0000 | 1.00 | 0.89 | 0.86 | 1.00 | 0.95 | 0.92 | 0.90 |
| 0016-0051 | iBFM | 2.00 | 0.71 | 1.0000 | 1.00 | 0.89 | 1.00 | 1.00 | 1.00 | 0.92 | 0.96 |
| 0016-0051 | iBFM | 3.00 | 0.63 | 1.0000 | 1.00 | 0.89 | 0.93 | 1.00 | 1.00 | 0.92 | 0.96 |
| 0016-0051 | iBFM | 5.00 | 0.71 | 1.0000 | 1.00 | 0.89 | 1.00 | 1.00 | 1.00 | 0.92 | 0.96 |
| 0016-0051 | iBFM | 6.00 | 0.71 | 1.0000 | 1.00 | 0.89 | 1.00 | 1.00 | 1.00 | 0.92 | 0.96 |
| 0016-0051 | iBFM | 7.00 | 0.39 | 1.0000 | 1.00 | 0.89 | 0.86 | 1.00 | 0.85 | 0.95 | 0.90 |
| 0016-0051 | iBFM | 8.00 | 0.65 | 1.0000 | 1.00 | 0.89 | 0.93 | 1.00 | 1.00 | 1.00 | 0.90 |
| 0016-0051 | iBFM | 9.00 | 0.87 | 1.00 | 1.00 | 0.94 | 1.00 | 1.00 | 1.00 | 1.00 | 0.96 |
| 0016-0051 | iBFM | 10.00 | 0.87 | 1.00 | 1.00 | 0.94 | 1.00 | 1.00 | 1.00 | 1.00 | 0.96 |
| 0016-0055 | iBFM | 1.00 | -0.14 | 1.00 | 1.00 | 1.00 | 0.58 | 1.00 | 0.64 | 0.83 | 0.55 |
| 0016-0056 | iBFM | 2.00 | 0.71 | 1.00 | 1.00 | 0.89 | 0.93 | 1.00 | 0.95 | 1.00 | 1.00 |
| 0016-0056 | iBFM | 6.00 | 0.36 | 1.00 | 1.00 | 0.89 | 0.73 | 1.00 | 0.85 | 1.00 | 0.96 |
| 0016-0056 | iBFM | 7.00 | 0.37 | 1.00 | 1.00 | 0.89 | 0.86 | 1.00 | 0.85 | 0.92 | 0.90 |
| 0016-0056 | iBFM | 9.00 | 0.62 | 1.00 | 1.00 | 0.89 | 1.00 | 1.00 | 0.85 | 1.00 | 0.96 |
| 0016-0056 | iBFM | 10.00 | 0.85 | 1.00 | 1.00 | 0.89 | 1.00 | 1.00 | 1.00 | 1.00 | 1.00 |
| 0016-0061 | iBFM | 3.00 | 0.95 | 1.00 | 1.00 | 1.00 | 1.00 | 1.00 | 1.00 | 1.00 | 0.96 |
| 0016-0061 | iBFM | 4.00 | 1.00 | 1.00 | 1.00 | 1.00 | 1.00 | 1.00 | 1.00 | 1.00 | 1.00 |
| 0016-0061 | iBFM | 5.00 | 0.95 | 1.00 | 1.00 | 1.00 | 1.00 | 1.00 | 1.00 | 1.00 | 0.96 |
| 0016-0061 | iBFM | 6.00 | 0.95 | 1.00 | 1.00 | 1.00 | 1.00 | 1.00 | 1.00 | 1.00 | 0.96 |
| 0016-0061 | iBFM | 7.00 | 0.88 | 1.00 | 1.00 | 1.00 | 1.00 | 1.00 | 0.95 | 1.00 | 0.96 |
| 0016-0061 | iBFM | 8.00 | 0.95 | 1.00 | 1.00 | 1.00 | 1.00 | 1.00 | 1.00 | 1.00 | 0.96 |
| 0016-0062 | iBFM | 1.00 | 0.48 | 1.00 | 1.00 | 1.00 | 0.65 | 1.00 | 1.00 | 1.00 | 0.96 |
| 0016-0062 | iBFM | 2.00 | 0.48 | 1.00 | 1.00 | 1.00 | 0.65 | 1.00 | 1.00 | 1.00 | 0.96 |

Note: 999.00=missing data

Data Table 1. HUI3 Data

| **ID** | **Protocol** | **Months Post-Diagnosis** | **Utility Score** | **Vision** | **Hearing** | **Speech** | **Ambulation** | **Dexterity** | **Emotion** | **Cognition** | **Pain** |
| --- | --- | --- | --- | --- | --- | --- | --- | --- | --- | --- | --- |
| 0016-0062 | iBFM | 3.00 | 0.73 | 1.00 | 1.00 | 1.00 | 1.00 | 0.95 | 1.00 | 0.96 | 0.88 |
| 0016-0062 | iBFM | 4.00 | 0.84 | 1.00 | 1.00 | 1.00 | 1.00 | 0.95 | 1.00 | 1.00 | 0.93 |
| 0016-0062 | iBFM | 5.00 | 0.57 | 1.00 | 1.00 | 1.00 | 1.00 | 0.95 | 1.00 | 0.90 | 0.80 |
| 0016-0062 | iBFM | 7.00 | 0.16 | 1.00 | 1.00 | 1.00 | 0.93 | 1.00 | 0.46 | 1.00 | 0.90 |
| 0016-0062 | iBFM | 12.00 | 0.72 | 1.00 | 1.00 | 1.00 | 0.93 | 1.00 | 0.95 | 1.00 | 0.90 |
| 0016-0066 | iBFM | 1.00 | 0.53 | 1.00 | 1.00 | 1.00 | 0.86 | 1.00 | 0.85 | 1.00 | 0.90 |
| 0016-0066 | iBFM | 2.00 | 0.59 | 1.00 | 1.00 | 1.00 | 0.73 | 1.00 | 1.00 | 1.00 | 0.96 |
| 0017-0019 | COG | 7.00 | 0.88 | 1.00 | 1.00 | 1.00 | 1.00 | 1.00 | 0.95 | 1.00 | 0.96 |
| 0017-0019 | COG | 8.00 | 0.95 | 1.00 | 1.00 | 1.00 | 1.00 | 1.00 | 1.00 | 1.00 | 0.96 |
| 0017-0019 | COG | 9.00 | 0.80 | 1.00 | 1.00 | 1.00 | 1.00 | 1.00 | 0.95 | 1.00 | 0.90 |
| 0017-0019 | COG | 10.00 | 0.88 | 1.00 | 1.00 | 1.00 | 1.00 | 1.00 | 0.95 | 1.00 | 0.96 |
| 0017-0019 | COG | 11.00 | 0.78 | 1.00 | 1.00 | 1.00 | 1.00 | 1.00 | 0.95 | 0.92 | 0.96 |
| 0017-0019 | COG | 12.00 | 1.00 | 1.00 | 1.00 | 1.00 | 1.00 | 1.00 | 1.00 | 1.00 | 1.00 |
| 0017-0023 | COG | 6.00 | 0.79 | 1.00 | 1.00 | 1.00 | 0.93 | 1.00 | 0.95 | 1.00 | 0.96 |
| 0017-0023 | COG | 8.00 | 0.93 | 1.00 | 1.00 | 1.00 | 1.00 | 1.00 | 1.00 | 0.95 | 1.00 |
| 0017-0023 | COG | 10.00 | 0.89 | 1.00 | 1.00 | 1.00 | 1.00 | 1.00 | 1.00 | 0.92 | 1.00 |
| 0017-0023 | COG | 12.00 | 0.83 | 1.00 | 1.00 | 1.00 | 1.00 | 1.00 | 0.95 | 0.92 | 1.00 |
| 0017-0024 | COG | 6.00 | 1.00 | 1.00 | 1.00 | 1.00 | 1.00 | 1.00 | 1.00 | 1.00 | 1.00 |
| 0017-0024 | COG | 7.00 | 0.63 | 1.00 | 1.00 | 1.00 | 1.00 | 1.00 | 0.95 | 1.00 | 0.77 |
| 0017-0024 | COG | 9.00 | 1.00 | 1.00 | 1.00 | 1.00 | 1.00 | 1.00 | 1.00 | 1.00 | 1.00 |
| 0017-0024 | COG | 10.00 | 0.79 | 1.00 | 1.00 | 1.00 | 1.00 | 1.00 | 0.85 | 1.00 | 1.00 |
| 0017-0024 | COG | 11.00 | 1.00 | 1.00 | 1.00 | 1.00 | 1.00 | 1.00 | 1.00 | 1.00 | 1.00 |
| 0017-0024 | COG | 12.00 | 1.00 | 1.00 | 1.00 | 1.00 | 1.00 | 1.00 | 1.00 | 1.00 | 1.00 |
| 0017-0025 | COG | 5.00 | 0.79 | 1.00 | 1.00 | 1.00 | 0.93 | 1.00 | 0.95 | 1.00 | 0.96 |
| 0017-0025 | COG | 6.00 | 0.93 | 1.00 | 1.00 | 1.00 | 1.00 | 1.00 | 0.95 | 1.00 | 1.00 |
| 0017-0025 | COG | 8.00 | 0.88 | 1.00 | 1.00 | 1.00 | 1.00 | 1.00 | 0.95 | 1.00 | 0.96 |
| 0017-0025 | COG | 9.00 | 0.88 | 1.00 | 1.00 | 1.00 | 1.00 | 1.00 | 0.95 | 1.00 | 0.96 |
| 0017-0025 | COG | 10.00 | 0.80 | 1.00 | 1.00 | 0.94 | 1.00 | 1.00 | 0.95 | 1.00 | 0.96 |
| 0017-0027 | COG | 6.00 | 0.32 | 1.00 | 1.00 | 1.00 | 0.73 | 0.95 | 0.95 | 1.00 | 0.77 |
| 0017-0027 | COG | 7.00 | 0.32 | 1.00 | 1.00 | 1.00 | 0.73 | 0.95 | 0.95 | 1.00 | 0.77 |
| 0017-0027 | COG | 8.00 | 0.25 | 1.00 | 1.00 | 1.00 | 0.65 | 0.95 | 0.95 | 1.00 | 0.77 |
| 0017-0027 | COG | 9.00 | 0.25 | 1.00 | 1.00 | 1.00 | 0.65 | 0.95 | 0.95 | 1.00 | 0.77 |
| 0017-0027 | COG | 10.00 | 0.25 | 1.00 | 1.00 | 1.00 | 0.65 | 0.95 | 0.95 | 1.00 | 0.77 |
| 0017-0027 | COG | 11.00 | 0.32 | 1.00 | 1.00 | 1.00 | 0.73 | 0.95 | 0.95 | 1.00 | 0.77 |
| 0017-0027 | COG | 12.00 | 0.40 | 1.00 | 1.00 | 1.00 | 0.65 | 0.95 | 0.95 | 1.00 | 0.96 |

Note: 999.00=missing data

| 0017-0029 | COG | 4.00 | 0.17 | 1.00 | 1.00 | 1.00 | 0.73 | 1.00 | 0.85 | 0.83 | 0.77 |
| --- | --- | --- | --- | --- | --- | --- | --- | --- | --- | --- | --- |

Data Table 1. HUI3 Data

| **ID** | **Protocol** | **Months Post-Diagnosis** | **Utility Score** | **Vision** | **Hearing** | **Speech** | **Ambulation** | **Dexterity** | **Emotion** | **Cognition** | **Pain** |
| --- | --- | --- | --- | --- | --- | --- | --- | --- | --- | --- | --- |
| 0017-0029 | COG | 6.00 | 0.48 | 1.00 | 1.00 | 1.00 | 0.73 | 1.00 | 1.00 | 0.95 | 0.90 |
| 0017-0029 | COG | 9.00 | 0.62 | 1.00 | 1.00 | 1.00 | 0.93 | 1.00 | 0.85 | 0.95 | 0.96 |
| 0017-0030 | COG | 2.00 | 0.78 | 1.00 | 1.00 | 1.00 | 0.93 | 1.00 | 1.00 | 1.00 | 0.90 |
| 0017-0030 | COG | 4.00 | 1.00 | 1.00 | 1.00 | 1.00 | 1.00 | 1.00 | 1.00 | 1.00 | 1.00 |
| 0017-0030 | COG | 5.00 | 1.00 | 1.00 | 1.00 | 1.00 | 1.00 | 1.00 | 1.00 | 1.00 | 1.00 |
| 0017-0030 | COG | 6.00 | 0.95 | 1.00 | 1.00 | 1.00 | 1.00 | 1.00 | 1.00 | 1.00 | 0.96 |
| 0017-0030 | COG | 7.00 | 0.95 | 1.00 | 1.00 | 1.00 | 1.00 | 1.00 | 1.00 | 1.00 | 0.96 |
| 0017-0030 | COG | 8.00 | 0.95 | 1.00 | 1.00 | 1.00 | 1.00 | 1.00 | 1.00 | 1.00 | 0.96 |
| 0017-0031 | COG | 4.00 | 1.00 | 1.00 | 1.00 | 1.00 | 1.00 | 1.00 | 1.00 | 1.00 | 1.00 |
| 0017-0031 | COG | 8.00 | 0.88 | 1.00 | 1.00 | 1.00 | 1.00 | 1.00 | 0.95 | 1.00 | 0.96 |
| 0017-0031 | COG | 12.00 | 0.95 | 1.00 | 1.00 | 1.00 | 1.00 | 1.00 | 1.00 | 1.00 | 0.96 |
| 0017-0032 | COG | 1.00 | 0.90 | 1.00 | 1.00 | 1.00 | 0.93 | 1.00 | 1.00 | 1.00 | 1.00 |
| 0017-0032 | COG | 2.00 | 999.00 | 999.00 | 999.00 | 999.00 | 999.00 | 999.00 | 999.00 | 999.00 | 999.00 |
| 0017-0032 | COG | 5.00 | 0.79 | 1.00 | 1.00 | 1.00 | 0.93 | 1.00 | 0.95 | 1.00 | 0.96 |
| 0017-0034 | COG | 1.00 | 0.63 | 1.00 | 1.00 | 0.81 | 1.00 | 1.00 | 1.00 | 1.00 | 0.90 |
| 0017-0034 | COG | 2.00 | 0.74 | 1.00 | 1.00 | 0.81 | 1.00 | 1.00 | 1.00 | 1.00 | 1.00 |
| 0017-0034 | COG | 3.00 | 0.85 | 1.00 | 1.00 | 0.89 | 1.00 | 1.00 | 1.00 | 1.00 | 1.00 |
| 0017-0034 | COG | 4.00 | 0.92 | 1.00 | 1.00 | 0.94 | 1.00 | 1.00 | 1.00 | 1.00 | 1.00 |
| 0017-0034 | COG | 6.00 | 1.00 | 1.00 | 1.00 | 1.00 | 1.00 | 1.00 | 1.00 | 1.00 | 1.00 |
| 0017-0034 | COG | 7.00 | 1.00 | 1.00 | 1.00 | 1.00 | 1.00 | 1.00 | 1.00 | 1.00 | 1.00 |
| 0017-0034 | COG | 8.00 | 1.00 | 1.00 | 1.00 | 1.00 | 1.00 | 1.00 | 1.00 | 1.00 | 1.00 |
| 0017-0034 | COG | 9.00 | 1.00 | 1.00 | 1.00 | 1.00 | 1.00 | 1.00 | 1.00 | 1.00 | 1.00 |
| 0017-0034 | COG | 10.00 | 1.00 | 1.00 | 1.00 | 1.00 | 1.00 | 1.00 | 1.00 | 1.00 | 1.00 |
| 0017-0034 | COG | 11.00 | 1.00 | 1.00 | 1.00 | 1.00 | 1.00 | 1.00 | 1.00 | 1.00 | 1.00 |
| 0017-0034 | COG | 12.00 | 1.00 | 1.00 | 1.00 | 1.00 | 1.00 | 1.00 | 1.00 | 1.00 | 1.00 |
| 0017-0035 | COG | 1.00 | 0.39 | 1.00 | 1.00 | 1.00 | 0.65 | 1.00 | 0.95 | 1.00 | 0.90 |
| 0017-0035 | COG | 2.00 | 0.95 | 1.00 | 1.00 | 1.00 | 1.00 | 1.00 | 1.00 | 1.00 | 0.96 |
| 0017-0035 | COG | 5.00 | 0.87 | 1.00 | 1.00 | 0.94 | 1.00 | 1.00 | 1.00 | 1.00 | 0.96 |
| 0017-0036 | COG | 1.00 | 1.00 | 1.00 | 1.00 | 1.00 | 1.00 | 1.00 | 1.00 | 1.00 | 1.00 |
| 0017-0036 | COG | 2.00 | 0.60 | 1.00 | 1.00 | 0.89 | 0.93 | 0.95 | 1.00 | 1.00 | 0.90 |
| 0017-0036 | COG | 3.00 | 0.78 | 1.00 | 1.00 | 1.00 | 1.00 | 1.00 | 0.95 | 0.92 | 0.96 |
| 0017-0036 | COG | 4.00 | 0.79 | 1.00 | 1.00 | 1.00 | 0.93 | 1.00 | 0.95 | 1.00 | 0.96 |
| 0017-0036 | COG | 5.00 | 0.23 | 1.00 | 1.00 | 1.00 | 0.73 | 1.00 | 0.85 | 0.92 | 0.77 |
| 0017-0036 | COG | 6.00 | 0.80 | 1.00 | 1.00 | 1.00 | 1.00 | 1.00 | 0.95 | 1.00 | 0.90 |
| 0017-0036 | COG | 7.00 | 0.84 | 1.00 | 1.00 | 1.00 | 1.00 | 1.00 | 1.00 | 0.92 | 0.96 |

Note: 999.00=missing data

Data Table 1. HUI3 Data

| **ID** | **Protocol** | **Months Post-Diagnosis** | **Utility Score** | **Vision** | **Hearing** | **Speech** | **Ambulation** | **Dexterity** | **Emotion** | **Cognition** | **Pain** |
| --- | --- | --- | --- | --- | --- | --- | --- | --- | --- | --- | --- |
| 0017-0036 | COG | 8.00 | 0.74 | 1.00 | 1.00 | 1.00 | 0.93 | 1.00 | 0.95 | 0.92 | 1.00 |
| 0017-0036 | COG | 9.00 | 0.46 | 1.00 | 1.00 | 1.00 | 0.93 | 1.00 | 0.85 | 1.00 | 0.77 |
| 0017-0036 | COG | 10.00 | 0.78 | 1.00 | 1.00 | 1.00 | 0.93 | 1.00 | 1.00 | 1.00 | 0.90 |
| 0017-0036 | COG | 11.00 | 0.71 | 1.00 | 1.00 | 1.00 | 0.86 | 1.00 | 1.00 | 0.92 | 1.00 |
| 0017-0036 | COG | 12.00 | 0.85 | 1.00 | 1.00 | 1.00 | 0.93 | 1.00 | 1.00 | 1.00 | 0.96 |
| 0017-0038 | COG | 2.00 | 0.92 | 1.00 | 1.00 | 0.94 | 1.00 | 1.00 | 1.00 | 1.00 | 1.00 |
| 0017-0038 | COG | 3.00 | 0.90 | 1.00 | 1.00 | 1.00 | 0.93 | 1.00 | 1.00 | 1.00 | 1.00 |
| 0017-0038 | COG | 4.00 | 1.00 | 1.00 | 1.00 | 1.00 | 1.00 | 1.00 | 999.00.00 | 1.00 | 1.00 |
| 0017-0038 | COG | 5.00 | 0.92 | 1.00 | 1.00 | 0.94 | 1.00 | 1.00 | 1.00 | 1.00 | 1.00 |
| 0017-0038 | COG | 6.00 | 0.95 | 1.00 | 1.00 | 1.00 | 1.00 | 1.00 | 1.00 | 1.00 | 0.96 |
| 0017-0038 | COG | 7.00 | 0.88 | 1.00 | 1.00 | 1.00 | 1.00 | 1.00 | 1.00 | 0.95 | 0.96 |
| 0017-0038 | COG | 9.00 | 0.95 | 1.00 | 1.00 | 1.00 | 1.00 | 1.00 | 1.00 | 1.00 | 0.96 |
| 0017-0038 | COG | 10.00 | 1.00 | 1.00 | 1.00 | 1.00 | 1.00 | 1.00 | 1.00 | 1.00 | 1.00 |
| 0017-0038 | COG | 11.00 | 0.89 | 1.00 | 1.00 | 1.00 | 1.00 | 1.00 | 1.00 | 0.92 | 1.00 |
| 0017-0038 | COG | 12.00 | 0.77 | 1.00 | 1.00 | 1.00 | 1.00 | 1.00 | 1.00 | 0.83 | 1.00 |
| 0017-0040 | COG | 2.00 | 0.85 | 1.00 | 1.00 | 0.89 | 1.00 | 1.00 | 1.00 | 1.00 | 1.00 |
| 0017-0040 | COG | 3.00 | 1.00 | 1.00 | 1.00 | 1.00 | 1.00 | 1.00 | 1.00 | 1.00 | 1.00 |
| 0017-0040 | COG | 5.00 | 0.93 | 1.00 | 1.00 | 1.00 | 1.00 | 1.00 | 0.95 | 1.00 | 1.00 |
| 0017-0040 | COG | 7.00 | 1.00 | 1.00 | 1.00 | 1.00 | 1.00 | 1.00 | 1.00 | 1.00 | 1.00 |
| 0017-0040 | COG | 8.00 | 1.00 | 1.00 | 1.00 | 1.00 | 1.00 | 1.00 | 1.00 | 1.00 | 1.00 |
| 0017-0040 | COG | 9.00 | 1.00 | 1.00 | 1.00 | 1.00 | 1.00 | 1.00 | 1.00 | 1.00 | 1.00 |
| 0017-0043 | COG | 1.00 | 0.55 | 1.00 | 1.00 | 0.89 | 0.93 | 1.00 | 0.85 | 1.00 | 0.96 |
| 0017-0043 | COG | 2.00 | 0.74 | 1.00 | 1.00 | 0.89 | 1.00 | 1.00 | 0.95 | 1.00 | 0.96 |
| 0017-0043 | COG | 3.00 | 0.55 | 1.00 | 1.00 | 0.94 | 0.93 | 1.00 | 0.85 | 1.00 | 0.90 |
| 0017-0043 | COG | 4.00 | 0.72 | 1.00 | 1.00 | 0.94 | 0.93 | 1.00 | 0.95 | 1.00 | 0.96 |
| 0017-0043 | COG | 5.00 | 0.65 | 1.00 | 1.00 | 0.94 | 0.93 | 1.00 | 0.95 | 1.00 | 0.90 |
| 0017-0043 | COG | 7.00 | 0.87 | 1.00 | 1.00 | 0.94 | 1.00 | 1.00 | 1.00 | 1.00 | 0.96 |
| 0017-0043 | COG | 8.00 | 0.80 | 1.00 | 1.00 | 0.94 | 1.00 | 1.00 | 0.95 | 1.00 | 0.96 |
| 0017-0043 | COG | 9.00 | 1.00 | 1.00 | 1.00 | 1.00 | 1.00 | 1.00 | 1.00 | 1.00 | 1.00 |
| 0017-0043 | COG | 10.00 | 0.80 | 1.00 | 1.00 | 0.94 | 1.00 | 1.00 | 0.95 | 1.00 | 0.96 |
| 0017-0043 | COG | 11.00 | 0.80 | 1.00 | 1.00 | 0.94 | 1.00 | 1.00 | 0.95 | 1.00 | 0.96 |
| 0017-0045 | COG | 2.00 | 0.53 | 1.00 | 1.00 | 1.00 | 0.93 | 0.95 | 1.00 | 0.83 | 0.90 |
| 0017-0045 | COG | 4.00 | 0.23 | 1.00 | 0.95 | 0.94 | 0.73 | 0.95 | 0.95 | 0.83 | 0.90 |
| 0017-0045 | COG | 5.00 | 0.23 | 1.00 | 1.00 | 1.00 | 0.73 | 0.95 | 0.85 | 0.83 | 0.90 |
| 0017-0045 | COG | 7.00 | 0.22 | 1.00 | 1.00 | 1.00 | 0.93 | 0.65 | 0.95 | 0.83 | 0.90 |
| 0017-0045 | COG | 8.00 | 0.16 | 1.00 | 1.00 | 1.00 | 1.00 | 0.76 | 0.95 | 0.60 | 0.90 |

Note: 999.00=missing data

Data Table 1. HUI3 Data

| **ID** | **Protocol** | **Months Post-Diagnosis** | **Utility Score** | **Vision** | **Hearing** | **Speech** | **Ambulation** | **Dexterity** | **Emotion** | **Cognition** | **Pain** |
| --- | --- | --- | --- | --- | --- | --- | --- | --- | --- | --- | --- |
| 0017-0045 | COG | 10.00 | 0.35 | 1.00 | 1.00 | 0.94 | 0.93 | 0.95 | 0.85 | 0.83 | 0.90 |
| 0017-0045 | COG | 11.00 | 0.16 | 1.00 | 1.00 | 1.00 | 0.73 | 0.88 | 0.95 | 0.83 | 0.77 |
| 0017-0045 | COG | 12.00 | 0.28 | 1.00 | 1.00 | 1.00 | 0.93 | 1.00 | 0.95 | 0.60 | 0.90 |
| 0017-0047 | COG | 1.00 | 0.24 | 1.00 | 1.00 | 1.00 | 0.58 | 1.00 | 0.85 | 1.00 | 0.90 |
| 0017-0047 | COG | 2.00 | 0.41 | 1.00 | 1.00 | 0.68 | 1.00 | 1.00 | 0.95 | 0.92 | 0.96 |
| 0017-0047 | COG | 3.00 | 0.50 | 1.00 | 1.00 | 0.81 | 1.00 | 1.00 | 0.95 | 0.92 | 0.90 |
| 0017-0047 | COG | 4.00 | 0.37 | 0.98 | 1.00 | 0.68 | 1.00 | 1.00 | 0.85 | 1.00 | 0.96 |
| 0017-0047 | COG | 5.00 | 0.44 | 1.00 | 1.00 | 0.81 | 0.93 | 1.00 | 0.95 | 0.92 | 0.90 |
| 0017-0047 | COG | 6.00 | 0.80 | 1.00 | 1.00 | 0.89 | 1.00 | 1.00 | 1.00 | 1.00 | 0.96 |
| 0017-0047 | COG | 7.00 | 0.31 | 1.00 | 1.00 | 0.81 | 1.00 | 1.00 | 0.64 | 1.00 | 0.96 |
| 0017-0047 | COG | 8.00 | 0.56 | 1.00 | 1.00 | 0.81 | 1.00 | 1.00 | 0.95 | 0.92 | 0.96 |
| 0017-0047 | COG | 9.00 | 0.70 | 1.00 | 1.00 | 0.81 | 1.00 | 1.00 | 1.00 | 1.00 | 0.96 |
| 0017-0047 | COG | 10.00 | 0.32 | 0.84 | 0.95 | 0.81 | 0.93 | 1.00 | 0.95 | 0.92 | 0.96 |
| 0017-0047 | COG | 11.00 | 0.87 | 1.00 | 1.00 | 0.94 | 1.00 | 1.00 | 1.00 | 1.00 | 0.96 |
| 0017-0047 | COG | 12.00 | 0.50 | 0.98 | 0.95 | 0.81 | 1.00 | 1.00 | 0.95 | 0.92 | 0.96 |
| 0017-0048 | COG | 1.00 | 999.00 | 1.00 | 1.00 | 1.00 | 0.65 | 0.76 | 999.00.00 | 0.92 | 0.55 |
| 0017-0051 | COG | 1.00 | 0.54 | 1.00 | 1.00 | 0.94 | 1.00 | 1.00 | 0.85 | 0.92 | 0.90 |
| 0017-0051 | COG | 3.00 | 0.71 | 1.00 | 1.00 | 1.00 | 1.00 | 1.00 | 0.95 | 0.92 | 0.90 |
| 0017-0051 | COG | 4.00 | 0.50 | 1.00 | 1.00 | 0.94 | 0.93 | 1.00 | 0.85 | 0.95 | 0.90 |
| 0017-0051 | COG | 7.00 | 0.11 | 1.00 | 1.00 | 0.94 | 0.73 | 1.00 | 0.64 | 0.83 | 0.96 |
| 0017-0051 | COG | 8.00 | 0.32 | 1.00 | 1.00 | 1.00 | 0.73 | 0.95 | 0.85 | 0.95 | 0.90 |
| 0017-0054 | COG | 1.00 | 0.54 | 1.00 | 1.00 | 0.94 | 1.00 | 1.00 | 0.85 | 0.92 | 0.90 |
| 0017-0054 | COG | 2.00 | 0.17 | 1.00 | 1.00 | 0.94 | 0.93 | 1.00 | 0.64 | 0.92 | 0.77 |
| 0017-0054 | COG | 3.00 | 0.63 | 1.00 | 1.00 | 0.94 | 0.93 | 1.00 | 0.95 | 0.92 | 0.96 |
| 0017-0054 | COG | 4.00 | 0.47 | 1.00 | 1.00 | 0.94 | 0.93 | 1.00 | 0.85 | 0.92 | 0.90 |
| 0017-0054 | COG | 5.00 | 0.56 | 1.00 | 1.00 | 0.94 | 0.93 | 1.00 | 0.85 | 0.95 | 0.96 |
| 0017-0054 | COG | 6.00 | 0.53 | 1.00 | 1.00 | 0.94 | 0.93 | 1.00 | 0.85 | 0.92 | 0.96 |
| 0017-0054 | COG | 7.00 | 0.53 | 1.00 | 1.00 | 0.94 | 0.93 | 1.00 | 0.85 | 0.92 | 0.96 |
| 0017-0054 | COG | 8.00 | 0.47 | 1.00 | 1.00 | 0.94 | 0.93 | 1.00 | 0.85 | 0.92 | 0.90 |
| 0017-0054 | COG | 9.00 | -0.07 | 1.00 | 1.00 | 0.94 | 0.73 | 1.00 | 0.64 | 0.92 | 0.55 |
| 0017-0054 | COG | 10.00 | 0.47 | 1.00 | 1.00 | 0.94 | 0.93 | 1.00 | 0.85 | 0.92 | 0.90 |
| 0017-0054 | COG | 11.00 | 0.30 | 1.00 | 1.00 | 1.00 | 0.73 | 0.95 | 0.85 | 0.92 | 0.90 |
| 0017-0054 | COG | 12.00 | 0.53 | 1.00 | 1.00 | 1.00 | 0.93 | 1.00 | 0.85 | 0.92 | 0.90 |

Note: 999.00=missing data

Data Table 1. HUI3 Data

| **ID** | **Protocol** | **Months Post-Diagnosis** | **Utility Score** | **Vision** | **Hearing** | **Speech** | **Ambulation** | **Dexterity** | **Emotion** | **Cognition** | **Pain** |
| --- | --- | --- | --- | --- | --- | --- | --- | --- | --- | --- | --- |
| 0017-0056 | COG | 1.00 | 0.45 | 1.00 | 1.00 | 0.68 | 1.00 | 1.00 | 1.00 | 0.92 | 0.96 |
| 0017-0056 | COG | 2.00 | 0.37 | 1.00 | 1.00 | 0.68 | 1.00 | 1.00 | 1.00 | 0.83 | 0.96 |
| 0017-0056 | COG | 3.00 | 0.52 | 1.00 | 1.00 | 0.89 | 1.00 | 1.00 | 0.95 | 1.00 | 0.77 |
| 0017-0056 | COG | 4.00 | 0.01 | 1.00 | 1.00 | 0.81 | 1.00 | 1.00 | 0.85 | 0.42 | 0.96 |
| 0017-0056 | COG | 5.00 | 0.48 | 1.00 | 1.00 | 0.81 | 1.00 | 1.00 | 0.85 | 1.00 | 0.90 |
| 0017-0056 | COG | 6.00 | 0.59 | 1.00 | 1.00 | 0.81 | 1.00 | 1.00 | 0.95 | 0.95 | 0.96 |
| 0017-0056 | COG | 7.00 | 0.59 | 1.00 | 1.00 | 0.81 | 1.00 | 1.00 | 0.95 | 0.95 | 0.96 |
| 0017-0056 | COG | 9.00 | 0.38 | 1.00 | 1.00 | 0.81 | 1.00 | 1.00 | 0.85 | 0.83 | 0.96 |
| 0017-0056 | COG | 11.00 | 0.38 | 1.00 | 1.00 | 0.81 | 1.00 | 1.00 | 0.85 | 0.83 | 0.96 |
| 0017-0056 | COG | 12.00 | 0.49 | 1.00 | 1.00 | 0.81 | 1.00 | 1.00 | 0.85 | 0.95 | 0.96 |
| 0017-0058 | COG | 1.00 | 0.93 | 1.00 | 1.00 | 1.00 | 1.00 | 1.00 | 0.95 | 1.00 | 1.00 |
| 0017-0059 | COG | 2.00 | 0.97 | 0.98 | 1.00 | 1.00 | 1.00 | 1.00 | 1.00 | 1.00 | 1.00 |
| 0017-0059 | COG | 3.00 | 0.91 | 0.98 | 1.00 | 1.00 | 1.00 | 1.00 | 0.95 | 1.00 | 1.00 |
| 0017-0059 | COG | 4.00 | 0.92 | 0.98 | 1.00 | 1.00 | 1.00 | 1.00 | 1.00 | 1.00 | 0.96 |
| 0017-0059 | COG | 5.00 | 0.76 | 0.98 | 1.00 | 1.00 | 1.00 | 1.00 | 0.95 | 0.92 | 0.96 |
| 0017-0059 | COG | 7.00 | 0.87 | 0.98 | 1.00 | 1.00 | 1.00 | 1.00 | 1.00 | 0.92 | 1.00 |
| 0017-0059 | COG | 8.00 | 0.76 | 0.98 | 1.00 | 1.00 | 1.00 | 1.00 | 0.95 | 0.92 | 0.96 |
| 0017-0059 | COG | 10.00 | 0.92 | 0.98 | 1.00 | 1.00 | 1.00 | 1.00 | 1.00 | 1.00 | 0.96 |
| 0017-0059 | COG | 11.00 | 0.92 | 0.98 | 1.00 | 1.00 | 1.00 | 1.00 | 1.00 | 1.00 | 0.96 |
| 0017-0059 | COG | 12.00 | 0.85 | 0.98 | 1.00 | 1.00 | 1.00 | 1.00 | 0.95 | 1.00 | 0.96 |
| 0017-0060 | COG | 2.00 | 0.63 | 1.00 | 1.00 | 1.00 | 0.73 | 1.00 | 1.00 | 1.00 | 1.00 |
| 0017-0060 | COG | 3.00 | 0.85 | 1.00 | 1.00 | 1.00 | 0.93 | 1.00 | 1.00 | 1.00 | 0.96 |
| 0017-0060 | COG | 7.00 | 1.00 | 1.00 | 1.00 | 1.00 | 1.00 | 1.00 | 1.00 | 1.00 | 1.00 |
| 0017-0060 | COG | 8.00 | 0.95 | 1.00 | 1.00 | 1.00 | 1.00 | 1.00 | 1.00 | 1.00 | 0.96 |
| 0017-0060 | COG | 11.00 | 0.95 | 1.00 | 1.00 | 1.00 | 1.00 | 1.00 | 1.00 | 1.00 | 0.96 |
| 0017-0063 | COG | 1.00 | -0.17 | 0.84 | 1.00 | 0.94 | 0.65 | 1.00 | 0.85 | 0.60 | 0.55 |
| 0017-0063 | COG | 2.00 | -0.25 | 0.84 | 0.80 | 0.81 | 0.73 | 0.76 | 0.85 | 0.60 | 0.55 |
| 0017-0063 | COG | 3.00 | -0.05 | 0.84 | 0.95 | 0.81 | 0.93 | 1.00 | 0.85 | 0.60 | 0.77 |
| 0017-0063 | COG | 4.00 | 0.18 | 0.98 | 1.00 | 0.81 | 0.93 | 1.00 | 0.95 | 0.60 | 0.96 |
| 0017-0063 | COG | 5.00 | 0.47 | 0.98 | 0.95 | 0.94 | 0.93 | 1.00 | 0.95 | 0.83 | 0.96 |
| 0017-0063 | COG | 6.00 | 0.28 | 0.98 | 0.95 | 0.81 | 0.93 | 1.00 | 0.85 | 0.83 | 0.96 |
| 0017-0063 | COG | 8.00 | 0.47 | 0.84 | 0.89 | 0.94 | 1.00 | 1.00 | 0.95 | 0.92 | 1.00 |
| 0017-0063 | COG | 9.00 | 0.53 | 0.98 | 0.95 | 0.81 | 1.00 | 1.00 | 0.95 | 0.92 | 1.00 |
| 0017-0063 | COG | 10.00 | 0.68 | 0.98 | 0.95 | 0.94 | 1.00 | 1.00 | 0.95 | 0.92 | 1.00 |
| 0019-0001 | COG | 5.00 | 0.92 | 1.00 | 1.00 | 0.94 | 1.00 | 1.00 | 1.00 | 1.00 | 1.00 |
| 0019-0001 | COG | 7.00 | 0.72 | 1.00 | 1.00 | 0.89 | 0.93 | 1.00 | 1.00 | 1.00 | 0.96 |

Note: 999.00=missing data

Data Table 1. HUI3 Data

| **ID** | **Protocol** | **Months Post-Diagnosis** | **Utility Score** | **Vision** | **Hearing** | **Speech** | **Ambulation** | **Dexterity** | **Emotion** | **Cognition** | **Pain** |
| --- | --- | --- | --- | --- | --- | --- | --- | --- | --- | --- | --- |
| 0019-0001 | COG | 8.00 | 0.81 | 1.00 | 1.00 | 0.94 | 1.00 | 1.00 | 1.00 | 0.92 | 1.00 |
| 0019-0001 | COG | 9.00 | 0.69 | 1.00 | 1.00 | 0.94 | 0.93 | 1.00 | 1.00 | 0.92 | 0.96 |
| 0019-0001 | COG | 10.00 | 0.92 | 1.0 | 1.00 | 0.94 | 1.00 | 1.00 | 1.00 | 1.00 | 1.00 |
| 0019-0001 | COG | 11.00 | 0.92 | 1.00 | 1.00 | 0.94 | 1.00 | 1.00 | 1.00 | 1.00 | 1.00 |
| 0019-0001 | COG | 12.00 | 0.81 | 1.00 | 1.00 | 0.94 | 1.00 | 1.00 | 1.00 | 0.92 | 1.00 |
| 0019-0006 | COG | 2.00 | 0.90 | 1.00 | 1.00 | 1.00 | 0.93 | 1.00 | 1.00 | 1.00 | 1.00 |
| 0019-0006 | COG | 4.00 | 0.68 | 1.00 | 1.00 | 1.00 | 0.93 | 1.00 | 1.00 | 0.92 | 0.90 |
| 0019-0008 | COG | 2.00 | 0.67 | 1.00 | 1.00 | 0.89 | 0.93 | 1.00 | 1.00 | 0.92 | 1.00 |
| 0019-0008 | COG | 3.00 | 0.45 | 1.00 | 1.00 | 0.81 | 0.93 | 1.00 | 1.00 | 0.83 | 0.96 |
| 0019-0008 | COG | 4.00 | 0.72 | 1.00 | 1.00 | 0.89 | 0.93 | 1.00 | 1.00 | 1.00 | 0.96 |
| 0019-0008 | COG | 5.00 | 0.56 | 1.00 | 1.00 | 0.81 | 1.00 | 1.00 | 0.95 | 0.92 | 0.96 |
| 0019-0008 | COG | 6.00 | 0.74 | 1.00 | 1.00 | 0.89 | 1.00 | 1.00 | 1.00 | 0.95 | 0.96 |
| 0019-0008 | COG | 7.00 | 0.65 | 1.00 | 1.00 | 0.89 | 1.00 | 1.00 | 0.95 | 0.92 | 0.96 |
| 0019-0008 | COG | 8.00 | 0.88 | 1.00 | 1.00 | 1.00 | 1.00 | 1.00 | 0.95 | 1.00 | 0.96 |
| 0019-0008 | COG | 9.00 | 0.50 | 1.00 | 1.00 | 0.89 | 0.93 | 1.00 | 0.85 | 1.00 | 0.90 |
| 0019-0008 | COG | 10.00 | 0.79 | 1.00 | 1.00 | 0.89 | 1.00 | 1.00 | 0.95 | 1.00 | 1.00 |
| 0019-0008 | COG | 11.00 | 0.39 | 1.00 | 1.00 | 0.89 | 0.93 | 0.95 | 0.85 | 0.92 | 0.90 |
| 0019-0008 | COG | 12.00 | 0.62 | 1.00 | 1.00 | 0.89 | 0.93 | 1.00 | 1.00 | 0.92 | 0.96 |
| 0019-0009 | COG | 4.00 | 0.31 | 1.00 | 1.00 | 0.94 | 0.73 | 0.95 | 0.85 | 1.00 | 0.90 |
| 0019-0009 | COG | 5.00 | 0.39 | 1.00 | 1.00 | 0.94 | 0.93 | 0.76 | 0.95 | 0.92 | 0.96 |
| 0019-0009 | COG | 6.00 | 0.52 | 1.00 | 1.00 | 0.94 | 0.93 | 0.95 | 0.95 | 0.92 | 0.90 |
| 0019-0009 | COG | 7.00 | 0.69 | 1.00 | 0.95 | 0.94 | 1.00 | 0.95 | 0.95 | 1.00 | 0.96 |
| 0019-0009 | COG | 8.00 | 0.60 | 1.00 | 1.00 | 0.89 | 0.93 | 0.95 | 1.00 | 1.00 | 0.90 |
| 0019-0009 | COG | 9.00 | 0.72 | 1.00 | 1.00 | 0.89 | 0.93 | 1.00 | 1.00 | 1.00 | 0.96 |
| 0019-0009 | COG | 10.00 | 0.59 | 1.00 | 1.00 | 0.94 | 0.86 | 0.95 | 0.95 | 1.00 | 0.96 |
| 0019-0009 | COG | 11.00 | 0.57 | 1.00 | 1.00 | 0.94 | 0.93 | 0.95 | 1.00 | 0.92 | 0.90 |
| 0019-0009 | COG | 12.00 | 0.73 | 1.00 | 1.00 | 0.94 | 0.93 | 1.00 | 1.00 | 0.92 | 1.00 |
| 0019-0015 | COG | 3.00 | 0.53 | 1.00 | 1.00 | 1.00 | 1.00 | 1.00 | 0.85 | 1.00 | 0.77 |
| 0019-0015 | COG | 5.00 | 0.21 | 1.00 | 1.00 | 1.00 | 0.93 | 1.00 | 0.64 | 0.92 | 0.77 |
| 0019-0015 | COG | 6.00 | 0.66 | 1.00 | 1.00 | 1.00 | 1.00 | 1.00 | 0.85 | 0.92 | 0.96 |
| 0019-0015 | COG | 7.00 | 0.70 | 1.00 | 1.00 | 1.00 | 0.93 | 1.00 | 0.95 | 0.92 | 0.96 |
| 0019-0015 | COG | 8.00 | 0.71 | 1.00 | 1.00 | 1.00 | 1.00 | 1.00 | 0.95 | 0.92 | 0.90 |
| 0019-0015 | COG | 10.00 | 0.56 | 1.00 | 1.00 | 1.00 | 0.93 | 1.00 | 0.85 | 0.95 | 0.90 |
| 0019-0015 | COG | 11.00 | 0.95 | 1.00 | 1.00 | 1.00 | 1.00 | 1.00 | 1.00 | 1.00 | 0.96 |

Note: 999.00=missing data

Data Table 1. HUI3 Data

| **ID** | **Protocol** | **Months Post-Diagnosis** | **Utility Score** | **Vision** | **Hearing** | **Speech** | **Ambulation** | **Dexterity** | **Emotion** | **Cognition** | **Pain** |
| --- | --- | --- | --- | --- | --- | --- | --- | --- | --- | --- | --- |
| 0019-0015 | COG | 12.00 | 0.84 | 1.00 | 1.00 | 1.00 | 1.00 | 1.00 | 1.00 | 0.92 | 0.96 |
| 0019-0017 | COG | 1.00 | 0.14 | 1.00 | 1.00 | 1.00 | 0.65 | 0.95 | 0.85 | 0.92 | 0.77 |
| 0019-0017 | COG | 3.00 | 0.78 | 1.00 | 1.00 | 1.00 | 0.93 | 1.00 | 1.00 | 1.00 | 0.90 |
| 0019-0017 | COG | 4.00 | 0.33 | 1.00 | 1.00 | 0.94 | 0.73 | 0.95 | 0.95 | 0.92 | 0.90 |
| 0019-0017 | COG | 5.00 | 0.42 | 1.00 | 1.00 | 0.94 | 0.86 | 1.00 | 0.95 | 0.83 | 0.90 |
| 0019-0017 | COG | 9.00 | 0.38 | 1.00 | 1.00 | 0.94 | 0.86 | 0.95 | 0.95 | 0.83 | 0.90 |
| 0024-0001 | COG | 8.00 | 0.95 | 1.00 | 1.00 | 1.00 | 1.00 | 1.00 | 1.00 | 1.00 | 0.96 |
| 0024-0001 | COG | 9.00 | 0.95 | 1.00 | 1.00 | 1.00 | 1.00 | 1.00 | 1.00 | 1.00 | 0.96 |
| 0024-0001 | COG | 10.00 | 0.95 | 1.00 | 1.00 | 1.00 | 1.00 | 1.00 | 1.00 | 1.00 | 0.96 |
| 0024-0001 | COG | 11.00 | 1.00 | 1.00 | 1.00 | 1.00 | 1.00 | 1.00 | 1.00 | 1.00 | 1.00 |
| 0024-0001 | COG | 12.00 | 1.00 | 1.00 | 1.00 | 1.00 | 1.00 | 1.00 | 1.00 | 1.00 | 1.00 |
| 0024-0002 | COG | 4.00 | 999.00.00 | 999.00.00 | 999.00.00 | 1.00 | 1.00 | 1.00 | 1.00 | 999.00 | 999.00 |
| 0024-0002 | COG | 6.00 | 999.00 | 1.00 | 1.00 | 999.00.00 | 1.00 | 1.00 | 0.95 | 1.00 | 0.96 |
| 0024-0002 | COG | 8.00 | 0.95 | 1.00 | 1.00 | 1.00 | 1.00 | 1.00 | 1.00 | 1.00 | 0.96 |
| 0024-0002 | COG | 11.00 | 1.00 | 1.00 | 1.00 | 1.00 | 1.00 | 1.00 | 1.00 | 1.00 | 1.00 |
| 0024-0002 | COG | 12.00 | 1.00 | 1.00 | 1.00 | 1.00 | 1.00 | 1.00 | 1.00 | 1.00 | 1.00 |
| 0024-0018 | COG | 1.00 | 0.44 | 1.00 | 1.00 | 1.00 | 0.65 | 1.00 | 0.95 | 1.00 | 0.96 |
| 0024-0018 | COG | 2.00 | 0.72 | 1.00 | 1.00 | 1.00 | 0.93 | 1.00 | 0.95 | 1.00 | 0.90 |
| 0024-0018 | COG | 3.00 | 0.88 | 1.00 | 1.00 | 1.00 | 1.00 | 1.00 | 0.95 | 1.00 | 0.96 |
| 0024-0018 | COG | 4.00 | 0.85 | 1.00 | 1.00 | 1.00 | 0.93 | 1.00 | 1.00 | 1.00 | 0.96 |
| 0024-0018 | COG | 5.00 | 0.79 | 1.00 | 1.00 | 1.00 | 0.93 | 1.00 | 0.95 | 1.00 | 0.96 |
| 0024-0018 | COG | 7.00 | 0.90 | 1.00 | 1.00 | 1.00 | 0.93 | 1.00 | 1.00 | 1.00 | 1.00 |
| 0024-0018 | COG | 9.00 | 0.85 | 1.00 | 1.00 | 1.00 | 0.93 | 1.00 | 1.00 | 1.00 | 0.96 |
| 0024-0018 | COG | 10.00 | 1.00 | 1.00 | 1.00 | 1.00 | 1.00 | 1.00 | 1.00 | 1.00 | 1.00 |
| 0024-0018 | COG | 11.00 | 0.62 | 0.98 | 1.00 | 1.00 | 0.93 | 0.95 | 0.95 | 0.92 | 0.96 |
| 0024-0018 | COG | 12.00 | 0.95 | 1.00 | 1.00 | 1.00 | 1.00 | 1.00 | 1.00 | 1.00 | 0.96 |
| 0024-0019 | COG | 2.00 | 0.62 | 0.98 | 1.00 | 1.00 | 0.93 | 0.95 | 0.95 | 0.92 | 0.96 |
| 0024-0019 | COG | 4.00 | 1.00 | 1.00 | 1.00 | 1.00 | 1.00 | 1.00 | 1.00 | 1.00 | 1.00 |
| 0024-0019 | COG | 5.00 | 1.00 | 1.00 | 1.00 | 1.00 | 1.00 | 1.00 | 1.00 | 1.00 | 1.00 |
| 0024-0019 | COG | 6.00 | 0.95 | 1.00 | 1.00 | 1.00 | 1.00 | 1.00 | 1.00 | 1.00 | 0.96 |
| 0024-0019 | COG | 7.00 | 1.00 | 1.00 | 1.00 | 1.00 | 1.00 | 1.00 | 1.00 | 1.00 | 1.00 |

Note: 999.00=missing data

Data Table 1. HUI3 Data

| **ID** | **Protocol** | **Months Post-Diagnosis** | **Utility Score** | **Vision** | **Hearing** | **Speech** | **Ambulation** | **Dexterity** | **Emotion** | **Cognition** | **Pain** |
| --- | --- | --- | --- | --- | --- | --- | --- | --- | --- | --- | --- |
| 0024-0019 | COG | 8.00 | 1.00 | 1.00 | 1.00 | 1.00 | 1.00 | 1.00 | 1.00 | 1.00 | 1.00 |
| 0024-0019 | COG | 10.00 | 1.00 | 1.00 | 1.00 | 1.00 | 1.00 | 1.00 | 1.00 | 1.00 | 1.00 |
| 0024-0019 | COG | 11.00 | 1.00 | 1.00 | 1.00 | 1.00 | 1.00 | 1.00 | 1.00 | 1.00 | 1.00 |
| 0024-0020 | COG | 1.00 | 0.52 | 0.98 | 1.00 | 0.89 | 1.00 | 1.00 | 1.00 | 0.83 | 0.90 |
| 0024-0020 | COG | 3.00 | 0.03 | 0.75 | 1.00 | 0.81 | 0.65 | 0.95 | 0.95 | 0.92 | 0.90 |
| 0024-0023 | COG | 2.00 | 0.78 | 1.00 | 1.00 | 1.00 | 0.93 | 1.00 | 1.00 | 1.00 | 0.90 |
| 0024-0023 | COG | 4.00 | 0.79 | 1.00 | 1.00 | 1.00 | 0.93 | 1.00 | 0.95 | 1.00 | 0.96 |
| 0024-0023 | COG | 8.00 | 0.66 | 1.00 | 1.00 | 1.00 | 0.86 | 1.00 | 0.95 | 0.92 | 1.00 |
| 0067-0002 | COG | 9.00 | 0.10 | 1.00 | 1.00 | 1.00 | 0.73 | 0.95 | 0.64 | 1.00 | 0.77 |
| 0067-0002 | COG | 10.00 | 0.63 | 1.00 | 1.00 | 1.00 | 0.73 | 1.00 | 1.00 | 1.00 | 1.00 |
| 0067-0002 | COG | 11.00 | 0.90 | 1.00 | 1.00 | 1.00 | 0.93 | 1.00 | 1.00 | 1.00 | 1.00 |
| 0067-0002 | COG | 12.00 | 1.00 | 1.00 | 1.00 | 1.00 | 1.00 | 1.00 | 1.00 | 1.00 | 1.00 |
| 0067-0004 | COG | 3.00 | 0.54 | 0.84 | 1.00 | 0.89 | 0.93 | 1.00 | 1.00 | 1.00 | 0.96 |
| 0067-0004 | COG | 4.00 | 0.78 | 1.00 | 1.00 | 1.00 | 0.93 | 1.00 | 1.00 | 1.00 | 0.90 |
| 0067-0004 | COG | 5.00 | 0.55 | 0.89 | 0.95 | 0.89 | 0.93 | 1.00 | 1.00 | 1.00 | 0.96 |
| 0067-0004 | COG | 6.00 | 0.87 | 1.00 | 1.00 | 0.94 | 1.00 | 1.00 | 1.00 | 1.00 | 0.96 |
| 0067-0004 | COG | 7.00 | 0.18 | 0.84 | 1.00 | 0.94 | 0.93 | 1.00 | 1.00 | 1.00 | 0.55 |
| 0067-0004 | COG | 8.00 | 0.69 | 1.00 | 1.00 | 0.94 | 0.93 | 1.00 | 1.00 | 0.92 | 0.96 |
| 0067-0004 | COG | 10.00 | 0.71 | 1.00 | 0.95 | 0.94 | 1.00 | 1.00 | 1.00 | 0.92 | 0.96 |
| 0067-0004 | COG | 11.00 | 0.49 | 0.84 | 0.89 | 0.94 | 0.93 | 1.00 | 1.00 | 1.00 | 0.96 |
| 0067-0004 | COG | 12.00 | 0.69 | 1.00 | 1.00 | 0.94 | 0.93 | 1.00 | 1.00 | 0.92 | 0.96 |
| 0067-0005 | COG | 3.00 | 0.85 | 1.00 | 1.00 | 1.00 | 0.93 | 1.00 | 1.00 | 1.00 | 0.96 |
| 0067-0005 | COG | 4.00 | 0.85 | 1.00 | 1.00 | 1.00 | 0.93 | 1.00 | 1.00 | 1.00 | 0.96 |
| 0067-0005 | COG | 5.00 | 0.93 | 1.00 | 1.00 | 1.00 | 1.00 | 0.95 | 1.00 | 1.00 | 1.00 |
| 0067-0005 | COG | 6.00 | 0.78 | 1.00 | 1.00 | 1.00 | 1.00 | 0.95 | 1.00 | 0.92 | 0.96 |
| 0067-0005 | COG | 7.00 | 0.88 | 1.00 | 1.00 | 1.00 | 1.00 | 1.00 | 1.00 | 0.95 | 0.96 |
| 0067-0005 | COG | 8.00 | 0.83 | 1.00 | 1.00 | 1.00 | 1.00 | 0.95 | 1.00 | 0.92 | 1.00 |
| 0067-0005 | COG | 9.00 | 0.83 | 1.00 | 1.00 | 1.00 | 1.00 | 0.95 | 1.00 | 0.92 | 1.00 |
| 0067-0005 | COG | 10.00 | 0.82 | 1.00 | 1.00 | 1.00 | 1.00 | 0.95 | 1.00 | 0.95 | 0.96 |
| 0067-0005 | COG | 11.00 | 0.55 | 1.00 | 1.00 | 1.00 | 0.93 | 0.95 | 0.95 | 0.83 | 0.96 |
| 0067-0005 | COG | 12.00 | 0.84 | 1.00 | 1.00 | 1.00 | 1.00 | 1.00 | 1.00 | 0.92 | 0.96 |
| 0067-0006 | COG | 3.00 | 0.85 | 1.00 | 1.00 | 1.00 | 0.93 | 1.00 | 1.00 | 1.00 | 0.96 |

Note: 999.00=missing data

Data Table 1. HUI3 Data

| **ID** | **Protocol** | **Months Post-Diagnosis** | **Utility Score** | **Vision** | **Hearing** | **Speech** | **Ambulation** | **Dexterity** | **Emotion** | **Cognition** | **Pain** |
| --- | --- | --- | --- | --- | --- | --- | --- | --- | --- | --- | --- |
| 0067-0007 | COG | 1.00 | 0.85 | 1.00 | 1.00 | 0.94 | 1.00 | 1.00 | 0.95 | 1.00 | 1.00 |
| 0067-0007 | COG | 2.00 | 0.61 | 0.98 | 1.00 | 0.94 | 0.93 | 1.00 | 0.95 | 0.92 | 0.96 |
| 0067-0007 | COG | 3.00 | 0.70 | 0.98 | 1.00 | 0.94 | 0.93 | 1.00 | 0.95 | 1.00 | 0.96 |
| 0067-0007 | COG | 4.00 | 0.72 | 1.00 | 1.00 | 0.94 | 0.93 | 1.00 | 0.95 | 1.00 | 0.96 |
| 0067-0007 | COG | 5.00 | 0.92 | 1.00 | 1.00 | 0.94 | 1.00 | 1.00 | 1.00 | 1.00 | 1.00 |
| 0067-0007 | COG | 6.00 | 0.73 | 1.00 | 1.00 | 0.94 | 1.00 | 1.00 | 0.95 | 1.00 | 0.90 |
| 0067-0007 | COG | 7.00 | 0.80 | 1.00 | 1.00 | 0.94 | 1.00 | 1.00 | 0.95 | 1.00 | 0.96 |
| 0067-0007 | COG | 8.00 | 0.80 | 1.00 | 1.00 | 0.94 | 1.00 | 1.00 | 0.95 | 1.00 | 0.96 |
| 0067-0007 | COG | 9.00 | 0.80 | 1.00 | 1.00 | 0.94 | 1.00 | 1.00 | 0.95 | 1.00 | 0.96 |
| 0067-0007 | COG | 10.00 | 0.97 | 0.98 | 1.00 | 1.00 | 1.00 | 1.00 | 1.00 | 1.00 | 1.00 |
| 0067-0007 | COG | 11.00 | 0.78 | 1.00 | 1.00 | 0.94 | 0.93 | 1.00 | 1.00 | 1.00 | 0.96 |
| 0067-0007 | COG | 12.00 | 0.84 | 0.98 | 1.00 | 0.94 | 1.00 | 1.00 | 1.00 | 1.00 | 0.96 |
| 0067-0008 | COG | 2.00 | 0.54 | 1.00 | 1.00 | 1.00 | 0.73 | 1.00 | 0.95 | 1.00 | 0.96 |
| 0067-0008 | COG | 5.00 | 0.78 | 1.00 | 1.00 | 0.94 | 0.93 | 1.00 | 1.00 | 1.00 | 0.96 |
| 0067-0008 | COG | 6.00 | 0.78 | 1.00 | 1.00 | 0.94 | 0.93 | 1.00 | 1.00 | 1.00 | 0.96 |
| 0067-0008 | COG | 7.00 | 0.87 | 1.00 | 1.00 | 0.94 | 1.00 | 1.00 | 1.00 | 1.00 | 0.96 |
| 0067-0008 | COG | 8.00 | 0.92 | 1.00 | 1.00 | 0.94 | 1.00 | 1.00 | 1.00 | 1.00 | 1.00 |
| 0067-0008 | COG | 10.00 | 0.87 | 1.00 | 1.00 | 0.94 | 1.00 | 1.00 | 1.00 | 1.00 | 0.96 |

Note: 999.00=missing data

Data Table 2. PedsQL Cancer Data

| **ID** | **Protocol** | **Months**  **Post-Diagnosis** | **PedsQL Pain** | **PedsQL Nausea** | **PedsQL Procedural Anxiety** | **PedsQL Treatment Anxiety** | **PedsQL Worry** | **PedsQL Cognitive Function** | **PedsQL Physical Appearance** | **PedsQL Communication** | **PedsQL Total** |
| --- | --- | --- | --- | --- | --- | --- | --- | --- | --- | --- | --- |
| 0013-0001 | iBFM | 7.00 | 75.00 | 55.00 | 100.00 | 100.00 | 83.33 | 93.75 | 58.33 | 83.33 | 81.09 |
| 0013-0001 | iBFM | 8.00 | 75.00 | 35.00 | 100.00 | 100.00 | 75.00 | 100.00 | 50.00 | 75.00 | 76.25 |
| 0013-0001 | iBFM | 9.00 | 62.50 | 65.00 | 100.00 | 83.33 | 75.00 | 93.75 | 66.67 | 91.67 | 79.74 |
| 0013-0001 | iBFM | 10.00 | 62.50 | 60.00 | 83.33 | 75.00 | 50.00 | 100.00 | 58.33 | 66.67 | 69.48 |
| 0013-0001 | iBFM | 11.00 | 100.00 | 75.00 | 100.00 | 91.67 | 66.67 | 100.00 | 83.33 | 100.00 | 89.58 |
| 0013-0001 | iBFM | 12.00 | 62.50 | 55.00 | 100.00 | 100.00 | 58.33 | 81.25 | 75.00 | 75.00 | 75.89 |
| 0013-0003 | iBFM | 4.00 | 87.50 | 45.00 | 25.00 | 75.00 | 100.00 | 100.00 | 100.00 | 75.00 | 75.94 |
| 0013-0003 | iBFM | 5.00 | 75.00 | 30.00 | 33.33 | 100.00 | 100.00 | 100.00 | 100.00 | 100.00 | 79.79 |
| 0013-0003 | iBFM | 6.00 | 75.00 | 30.00 | 41.67 | 100.00 | 100.00 | 100.00 | 100.00 | 100.00 | 80.83 |
| 0013-0003 | iBFM | 7.00 | 100.00 | 85.00 | 100.00 | 100.00 | 100.00 | 100.00 | 100.00 | 100.00 | 98.13 |
| 0013-0003 | iBFM | 8.00 | 50.00 | 45.00 | 50.00 | 100.00 | 100.00 | 100.00 | 100.00 | 100.00 | 80.63 |
| 0013-0003 | iBFM | 9.00 | 75.00 | 70.00 | 50.00 | 100.00 | 100.00 | 100.00 | 100.00 | 100.00 | 86.88 |
| 0013-0003 | iBFM | 10.00 | 87.50 | 60.00 | 91.67 | 100.00 | 100.00 | 100.00 | 100.00 | 100.00 | 92.40 |
| 0013-0003 | iBFM | 11.00 | 37.50 | 40.00 | 75.00 | 100.00 | 100.00 | 100.00 | 100.00 | 100.00 | 81.56 |
| 0013-0003 | iBFM | 12.00 | 75.00 | 65.00 | 91.67 | 100.00 | 100.00 | 100.00 | 100.00 | 100.00 | 91.46 |
| 0013-0004 | iBFM | 4.00 | 75.00 | 68.75 | 66.67 | 75.00 | 66.67 | 75.00 | 83.33 | 8.33 | 64.84 |
| 0013-0004 | iBFM | 5.00 | 62.50 | 75.00 | 83.33 | 75.00 | 66.67 | 66.67 | 50.00 | 16.67 | 61.98 |
| 0013-0004 | iBFM | 6.00 | 25.00 | 68.75 | 50.00 | 75.00 | 100.00 | 41.67 | 75.00 | 16.67 | 56.51 |
| 0013-0004 | iBFM | 7.00 | 37.50 | 65.00 | 66.67 | 75.00 | 100.00 | 75.00 | 50.00 | 50.00 | 64.90 |
| 0013-0004 | iBFM | 8.00 | 87.50 | 95.00 | 50.00 | 75.00 | 91.67 | 75.00 | 66.67 | 100.00 | 80.10 |
| 0013-0004 | iBFM | 10.00 | 75.00 | 95.00 | 25.00 | 66.67 | 75.00 | 58.33 | 66.67 | 50.00 | 63.96 |
| 0013-0004 | iBFM | 11.00 | 75.00 | 100.00 | 50.00 | 91.67 | 66.67 | 50.00 | 66.67 | 50.00 | 68.75 |
| 0013-0004 | iBFM | 12.00 | 87.50 | 95.00 | 50.00 | 100.00 | 66.67 | 66.67 | 75.00 | 83.33 | 78.02 |
| 0013-0005 | iBFM | 3.00 | 37.50 | 45.00 | 58.33 | 66.67 | 100.00 | 58.33 | 100.00 | 8.33 | 59.27 |
| 0013-0005 | iBFM | 4.00 | 37.50 | 35.00 | 50.00 | 58.33 | 100.00 | 75.00 | 100.00 | 16.67 | 59.06 |
| 0013-0005 | iBFM | 5.00 | 75.00 | 55.00 | 50.00 | 58.33 | 100.00 | 58.33 | 75.00 | 16.67 | 61.04 |
| 0013-0005 | iBFM | 7.00 | 87.50 | 90.00 | 83.33 | 58.33 | 100.00 | 75.00 | 100.00 | 8.33 | 75.31 |
| 0013-0005 | iBFM | 9.00 | 100.00 | 95.00 | 25.00 | 75.00 | 100.00 | 91.67 | 100.00 | 0.00 | 73.33 |
| 0013-0005 | iBFM | 10.00 | 100.00 | 100.00 | 25.00 | 100.00 | 100.00 | 75.00 | 100.00 | 75.00 | 84.38 |
| 0013-0006 | iBFM | 4.00 | 25.00 | 65.00 | 58.33 | 33.33 | 41.67 | 31.25 | 25.00 | 50.00 | 41.20 |
| 0013-0006 | iBFM | 5.00 | 12.50 | 60.00 | 66.67 | 16.67 | 25.00 | 37.50 | 0.00 | 41.67 | 32.50 |
| 0013-0006 | iBFM | 7.00 | 0.00 | 55.00 | 66.67 | 33.33 | 41.67 | 43.75 | 0.00 | 25.00 | 33.18 |
| 0013-0006 | iBFM | 8.00 | 25.00 | 70.00 | 0.00 | 16.67 | 25.00 | 37.50 | 0.00 | 33.33 | 25.94 |

Note: 999.00=missing data

Data Table 2. PedsQL Cancer Data

| **ID** | **Protocol** | **Months**  **Post-Diagnosis** | **PedsQL Pain** | **PedsQL Nausea** | **PedsQL Procedural Anxiety** | **PedsQL Treatment Anxiety** | **PedsQL Worry** | **PedsQL Cognitive Function** | **PedsQL Physical Appearance** | **PedsQL Communication** | **PedsQL Total** |
| --- | --- | --- | --- | --- | --- | --- | --- | --- | --- | --- | --- |
| 0013-0006 | iBFM | 9.00 | 37.50 | 45.00 | 0.00 | 0.00 | 0.00 | 31.25 | 0.00 | 0.00 | 14.22 |
| 0013-0006 | iBFM | 11.00 | 50.00 | 50.00 | 33.33 | 33.33 | 25.00 | 43.75 | 8.33 | 50.00 | 36.72 |
| 0013-0006 | iBFM | 12.00 | 62.50 | 50.00 | 41.67 | 25.00 | 50.00 | 50.00 | 25.00 | 41.67 | 43.23 |
| 0013-0008 | iBFM | 5.00 | 75.00 | 55.00 | 75.00 | 100.00 | 100.00 | 100.00 | 100.00 | 100.00 | 88.13 |
| 0013-0008 | iBFM | 6.00 | 50.00 | 80.00 | 100.00 | 100.00 | 100.00 | 100.00 | 100.00 | 100.00 | 91.25 |
| 0013-0008 | iBFM | 7.00 | 62.50 | 60.00 | 100.00 | 100.00 | 100.00 | 100.00 | 100.00 | 100.00 | 90.31 |
| 0013-0008 | iBFM | 8.00 | 75.00 | 60.00 | 75.00 | 100.00 | 100.00 | 91.67 | 100.00 | 100.00 | 87.71 |
| 0013-0008 | iBFM | 9.00 | 87.50 | 90.00 | 100.00 | 100.00 | 100.00 | 100.00 | 100.00 | 100.00 | 97.19 |
| 0013-0008 | iBFM | 10.00 | 87.50 | 95.00 | 100.00 | 100.00 | 100.00 | 100.00 | 100.00 | 100.00 | 97.81 |
| 0013-0008 | iBFM | 11.00 | 87.50 | 100.00 | 100.00 | 100.00 | 100.00 | 100.00 | 100.00 | 100.00 | 98.44 |
| 0013-0008 | iBFM | 12.00 | 100.00 | 100.00 | 100.00 | 100.00 | 100.00 | 100.00 | 100.00 | 100.00 | 100.00 |
| 0014-0020 | COG | 3.00 | 50.00 | 75.00 | 100.00 | 100.00 | 25.00 | 33.33 | 100.00 | 41.67 | 65.63 |
| 0014-0020 | COG | 5.00 | 50.00 | 85.00 | 83.33 | 100.00 | 33.33 | 50.00 | 100.00 | 50.00 | 68.96 |
| 0014-0020 | COG | 6.00 | 50.00 | 75.00 | 100.00 | 100.00 | 83.33 | 66.67 | 100.00 | 50.00 | 78.13 |
| 0014-0024 | COG | 4.00 | 25.00 | 30.00 | 0.00 | 33.33 | 0.00 | 56.25 | 33.33 | 75.00 | 31.61 |
| 0014-0024 | COG | 5.00 | 25.00 | 25.00 | 16.67 | 16.67 | 8.33 | 62.50 | 16.67 | 75.00 | 30.73 |
| 0014-0024 | COG | 6.00 | 50.00 | 20.00 | 16.67 | 25.00 | 8.33 | 66.67 | 50.00 | 75.00 | 38.96 |
| 0014-0024 | COG | 7.00 | 50.00 | 20.00 | 50.00 | 41.67 | 25.00 | 50.00 | 8.33 | 75.00 | 40.00 |
| 0014-0024 | COG | 8.00 | 50.00 | 20.00 | 0.00 | 16.67 | 50.00 | 50.00 | 50.00 | 66.67 | 37.92 |
| 0014-0024 | COG | 10.00 | 25.00 | 15.00 | 25.00 | 16.67 | 25.00 | 25.00 | 25.00 | 58.33 | 26.88 |
| 0014-0024 | COG | 11.00 | 25.00 | 15.00 | 0.00 | 0.00 | 0.00 | 18.75 | 8.33 | 58.33 | 15.68 |
| 0014-0024 | COG | 12.00 | 50.00 | 30.00 | 0.00 | 41.67 | 25.00 | 43.75 | 33.33 | 58.33 | 35.26 |
| 0014-0025 | COG | 1.00 | 50.00 | 40.00 | 33.33 | 58.33 | 100.00 | 66.67 | 58.33 | 58.33 | 58.13 |
| 0014-0025 | COG | 2.00 | 50.00 | 55.00 | 50.00 | 75.00 | 100.00 | 41.67 | 66.67 | 41.67 | 60.00 |
| 0014-0025 | COG | 4.00 | 25.00 | 35.00 | 33.33 | 50.00 | 91.67 | 41.67 | 50.00 | 41.67 | 46.04 |
| 0014-0025 | COG | 5.00 | 25.00 | 40.00 | 50.00 | 58.33 | 83.33 | 50.00 | 50.00 | 41.67 | 49.79 |
| 0015-0011 | COG | 5.00 | 62.50 | 70.00 | 0.00 | 50.00 | 91.67 | 91.67 | 100.00 | 50.00 | 64.48 |
| 0015-0011 | COG | 7.00 | 62.50 | 85.00 | 50.00 | 75.00 | 100.00 | 75.00 | 100.00 | 83.33 | 78.85 |
| 0015-0011 | COG | 8.00 | 75.00 | 100.00 | 50.00 | 75.00 | 100.00 | 100.00 | 100.00 | 83.33 | 85.42 |
| 0015-0011 | COG | 9.00 | 75.00 | 100.00 | 50.00 | 100.00 | 100.00 | 100.00 | 100.00 | 66.67 | 86.46 |

Note: 999.00=missing data

Data Table 2. PedsQL Cancer Data

| **ID** | **Protocol** | **Months**  **Post-Diagnosis** | **PedsQL Pain** | **PedsQL Nausea** | **PedsQL Procedural Anxiety** | **PedsQL Treatment Anxiety** | **PedsQL Worry** | **PedsQL Cognitive Function** | **PedsQL Physical Appearance** | **PedsQL Communication** | **PedsQL Total** |
| --- | --- | --- | --- | --- | --- | --- | --- | --- | --- | --- | --- |
| 0015-0012 | COG | 6.00 | 25.00 | 35.00 | 33.33 | 50.00 | 41.67 | 43.75 | 33.33 | 50.00 | 39.01 |
| 0015-0012 | COG | 8.00 | 37.50 | 65.00 | 50.00 | 50.00 | 50.00 | 37.50 | 41.67 | 25.00 | 44.58 |
| 0015-0012 | COG | 11.00 | 50.00 | 65.00 | 50.00 | 50.00 | 50.00 | 37.50 | 41.67 | 25.00 | 46.15 |
| 0015-0012 | COG | 12.00 | 50.00 | 55.00 | 50.00 | 58.33 | 50.00 | 31.25 | 50.00 | 58.33 | 50.36 |
| 0015-0018 | COG | 3.00 | 100.00 | 75.00 | 50.00 | 50.00 | 83.33 | 83.33 | 66.67 | 75.00 | 72.92 |
| 0015-0018 | COG | 6.00 | 50.00 | 55.00 | 16.67 | 50.00 | 75.00 | 75.00 | 50.00 | 91.67 | 57.92 |
| 0015-0018 | COG | 7.00 | 62.50 | 55.00 | 33.33 | 58.33 | 75.00 | 66.67 | 75.00 | 83.33 | 63.65 |
| 0015-0018 | COG | 9.00 | 50.00 | 80.00 | 16.67 | 75.00 | 58.33 | 58.33 | 75.00 | 83.33 | 62.08 |
| 0015-0018 | COG | 10.00 | 50.00 | 85.00 | 33.33 | 75.00 | 58.33 | 58.33 | 58.33 | 83.33 | 62.71 |
| 0015-0018 | COG | 11.00 | 75.00 | 75.00 | 50.00 | 50.00 | 50.00 | 58.33 | 33.33 | 66.67 | 57.29 |
| 0015-0018 | COG | 12.00 | 75.00 | 70.00 | 58.33 | 58.33 | 50.00 | 58.33 | 58.33 | 83.33 | 63.96 |
| 0015-0019 | COG | 2.00 | 50.00 | 60.00 | 50.00 | 83.33 | 50.00 | 62.50 | 50.00 | 91.67 | 62.19 |
| 0015-0019 | COG | 3.00 | 50.00 | 80.00 | 50.00 | 91.67 | 66.67 | 81.25 | 50.00 | 100.00 | 71.20 |
| 0015-0019 | COG | 4.00 | 62.50 | 75.00 | 50.00 | 91.67 | 50.00 | 75.00 | 50.00 | 91.67 | 68.23 |
| 0015-0019 | COG | 5.00 | 87.50 | 55.00 | 999.00 | 75.00 | 83.33 | 68.75 | 66.67 | 100.00 | 76.61 |
| 0015-0019 | COG | 6.00 | 75.00 | 75.00 | 75.00 | 83.33 | 91.67 | 50.00 | 50.00 | 91.67 | 73.96 |
| 0015-0019 | COG | 8.00 | 62.50 | 70.00 | 66.67 | 83.33 | 100.00 | 50.00 | 66.67 | 100.00 | 74.90 |
| 0015-0019 | COG | 9.00 | 62.50 | 75.00 | 75.00 | 91.67 | 100.00 | 50.00 | 58.33 | 91.67 | 75.52 |
| 0015-0019 | COG | 10.00 | 62.50 | 95.00 | 66.67 | 91.67 | 100.00 | 50.00 | 83.33 | 91.67 | 80.10 |
| 0015-0019 | COG | 11.00 | 75.00 | 100.00 | 66.67 | 91.67 | 58.33 | 62.50 | 66.67 | 100.00 | 77.60 |
| 0015-0019 | COG | 12.00 | 999.00 | 100.00 | 83.33 | 91.67 | 83.33 | 58.33 | 83.33 | 100.00 | 85.71 |
| 0015-0020 | COG | 2.00 | 75.00 | 75.00 | 50.00 | 58.33 | 100.00 | 58.33 | 100.00 | 83.33 | 75.00 |
| 0015-0020 | COG | 3.00 | 87.50 | 50.00 | 41.67 | 50.00 | 100.00 | 66.67 | 100.00 | 75.00 | 71.35 |
| 0015-0020 | COG | 7.00 | 62.50 | 60.00 | 83.33 | 100.00 | 100.00 | 75.00 | 100.00 | 75.00 | 81.98 |
| 0015-0020 | COG | 8.00 | 62.50 | 70.00 | 75.00 | 100.00 | 100.00 | 75.00 | 100.00 | 75.00 | 82.19 |
| 0015-0020 | COG | 9.00 | 62.50 | 70.00 | 75.00 | 83.33 | 100.00 | 58.33 | 100.00 | 100.00 | 81.15 |
| 0015-0020 | COG | 11.00 | 75.00 | 70.00 | 100.00 | 100.00 | 100.00 | 83.33 | 100.00 | 100.00 | 91.04 |
| 0015-0021 | COG | 3.00 | 50.00 | 45.00 | 66.67 | 100.00 | 100.00 | 100.00 | 100.00 | 91.67 | 81.67 |
| 0015-0021 | COG | 4.00 | 75.00 | 55.00 | 33.33 | 91.67 | 100.00 | 75.00 | 100.00 | 91.67 | 77.71 |
| 0015-0021 | COG | 5.00 | 75.00 | 45.00 | 58.33 | 91.67 | 100.00 | 58.33 | 100.00 | 100.00 | 78.54 |

Note: 999.00=missing data

Data Table 2. PedsQL Cancer Data

| **ID** | **Protocol** | **Months**  **Post-Diagnosis** | **PedsQL Pain** | **PedsQL Nausea** | **PedsQL Procedural Anxiety** | **PedsQL Treatment Anxiety** | **PedsQL Worry** | **PedsQL Cognitive Function** | **PedsQL Physical Appearance** | **PedsQL Communication** | **PedsQL Total** |
| --- | --- | --- | --- | --- | --- | --- | --- | --- | --- | --- | --- |
| 0015-0021 | COG | 6.00 | 50.00 | 60.00 | 58.33 | 100.00 | 100.00 | 91.67 | 100.00 | 100.00 | 82.50 |
| 0015-0021 | COG | 7.00 | 87.50 | 95.00 | 100.00 | 100.00 | 100.00 | 100.00 | 100.00 | 100.00 | 97.81 |
| 0015-0021 | COG | 8.00 | 100.00 | 85.00 | 100.00 | 100.00 | 100.00 | 100.00 | 100.00 | 100.00 | 98.13 |
| 0015-0022 | COG | 2.00 | 87.50 | 75.00 | 83.33 | 75.00 | 50.00 | 91.67 | 75.00 | 83.33 | 77.60 |
| 0015-0022 | COG | 3.00 | 75.00 | 50.00 | 33.33 | 66.67 | 41.67 | 66.67 | 58.33 | 100.00 | 61.46 |
| 0015-0022 | COG | 4.00 | 62.50 | 60.00 | 66.67 | 100.00 | 50.00 | 91.67 | 58.33 | 100.00 | 73.65 |
| 0015-0022 | COG | 6.00 | 87.50 | 50.00 | 66.67 | 100.00 | 75.00 | 100.00 | 91.67 | 100.00 | 83.85 |
| 0016-0005 | iBFM | 8.00 | 62.50 | 65.00 | 25.00 | 100.00 | 100.00 | 66.67 | 100.00 | 66.67 | 73.23 |
| 0016-0005 | iBFM | 9.00 | 87.50 | 95.00 | 50.00 | 100.00 | 100.00 | 83.33 | 100.00 | 66.67 | 85.31 |
| 0016-0005 | iBFM | 10.00 | 100.00 | 80.00 | 58.33 | 100.00 | 100.00 | 75.00 | 100.00 | 91.67 | 88.13 |
| 0016-0005 | iBFM | 11.00 | 87.50 | 95.00 | 33.33 | 100.00 | 100.00 | 58.33 | 100.00 | 41.67 | 76.98 |
| 0016-0005 | iBFM | 12.00 | 100.00 | 100.00 | 58.33 | 100.00 | 100.00 | 75.00 | 100.00 | 100.00 | 91.67 |
| 0016-0008 | iBFM | 2.00 | 75.00 | 40.00 | 41.67 | 66.67 | 100.00 | 33.33 | 100.00 | 50.00 | 63.33 |
| 0016-0008 | iBFM | 4.00 | 75.00 | 50.00 | 25.00 | 50.00 | 75.00 | 58.33 | 75.00 | 66.67 | 59.38 |
| 0016-0008 | iBFM | 5.00 | 87.50 | 55.00 | 25.00 | 50.00 | 91.67 | 33.33 | 100.00 | 83.33 | 65.73 |
| 0016-0010 | iBFM | 2.00 | 50.00 | 35.00 | 0.00 | 25.00 | 83.33 | 75.00 | 83.33 | 75.00 | 53.33 |
| 0016-0010 | iBFM | 5.00 | 87.50 | 55.00 | 16.67 | 50.00 | 100.00 | 58.33 | 75.00 | 100.00 | 67.81 |
| 0016-0010 | iBFM | 6.00 | 75.00 | 40.00 | 8.33 | 25.00 | 41.67 | 58.33 | 58.33 | 100.00 | 50.83 |
| 0016-0010 | iBFM | 7.00 | 50.00 | 20.00 | 0.00 | 50.00 | 66.67 | 66.67 | 33.33 | 100.00 | 48.33 |
| 0016-0010 | iBFM | 8.00 | 87.50 | 45.00 | 25.00 | 66.67 | 66.67 | 75.00 | 83.33 | 100.00 | 68.65 |
| 0016-0010 | iBFM | 9.00 | 87.50 | 90.00 | 16.67 | 58.33 | 83.33 | 83.33 | 100.00 | 100.00 | 77.40 |
| 0016-0010 | iBFM | 10.00 | 100.00 | 75.00 | 0.00 | 58.33 | 75.00 | 75.00 | 58.33 | 91.67 | 66.67 |
| 0016-0010 | iBFM | 11.00 | 75.00 | 75.00 | 41.67 | 16.67 | 75.00 | 75.00 | 75.00 | 75.00 | 63.54 |
| 0016-0010 | iBFM | 12.00 | 87.50 | 55.00 | 16.67 | 16.67 | 75.00 | 83.33 | 58.33 | 75.00 | 58.44 |
| 0016-0026 | iBFM | 4.00 | 50.00 | 25.00 | 8.33 | 33.33 | 58.33 | 75.00 | 50.00 | 75.00 | 46.88 |
| 0016-0026 | iBFM | 5.00 | 62.50 | 25.00 | 25.00 | 33.33 | 75.00 | 58.33 | 50.00 | 50.00 | 47.40 |
| 0016-0026 | iBFM | 6.00 | 75.00 | 55.00 | 0.00 | 25.00 | 75.00 | 66.67 | 66.67 | 50.00 | 51.67 |
| 0016-0026 | iBFM | 7.00 | 62.50 | 65.00 | 0.00 | 16.67 | 58.33 | 58.33 | 66.67 | 16.67 | 43.02 |
| 0016-0026 | iBFM | 8.00 | 62.50 | 55.00 | 0.00 | 41.67 | 100.00 | 41.67 | 58.33 | 33.33 | 49.06 |
| 0016-0026 | iBFM | 9.00 | 75.00 | 80.00 | 33.33 | 41.67 | 75.00 | 50.00 | 50.00 | 50.00 | 56.88 |
| 0016-0026 | iBFM | 10.00 | 62.50 | 75.00 | 16.67 | 16.67 | 66.67 | 33.33 | 41.67 | 41.67 | 44.27 |

Note: 999.00=missing data

Data Table 2. PedsQL Cancer Data

| **ID** | **Protocol** | **Months**  **Post-Diagnosis** | **PedsQL Pain** | **PedsQL Nausea** | **PedsQL Procedural Anxiety** | **PedsQL Treatment Anxiety** | **PedsQL Worry** | **PedsQL Cognitive Function** | **PedsQL Physical Appearance** | **PedsQL Communication** | **PedsQL Total** |
| --- | --- | --- | --- | --- | --- | --- | --- | --- | --- | --- | --- |
| 0016-0026 | iBFM | 11.00 | 62.50 | 45.00 | 0.00 | 50.00 | 41.67 | 41.67 | 58.33 | 50.00 | 43.65 |
| 0016-0026 | iBFM | 12.00 | 75.00 | 60.00 | 41.67 | 33.33 | 58.33 | 58.33 | 50.00 | 50.00 | 53.33 |
| 0016-0032 | iBFM | 2.00 | 75.00 | 55.00 | 25.00 | 0.00 | 100.00 | 83.33 | 100.00 | 0.00 | 54.79 |
| 0016-0032 | iBFM | 3.00 | 100.00 | 60.00 | 25.00 | 25.00 | 100.00 | 58.33 | 100.00 | 50.00 | 64.79 |
| 0016-0032 | iBFM | 5.00 | 100.00 | 75.00 | 50.00 | 50.00 | 100.00 | 66.67 | 100.00 | 41.67 | 72.92 |
| 0016-0032 | iBFM | 6.00 | 100.00 | 65.00 | 25.00 | 75.00 | 100.00 | 66.67 | 100.00 | 0.00 | 66.46 |
| 0016-0032 | iBFM | 7.00 | 50.00 | 70.00 | 50.00 | 75.00 | 100.00 | 41.67 | 100.00 | 33.33 | 65.00 |
| 0016-0032 | iBFM | 8.00 | 87.50 | 75.00 | 25.00 | 41.67 | 100.00 | 58.33 | 100.00 | 33.33 | 65.10 |
| 0016-0032 | iBFM | 9.00 | 100.00 | 100.00 | 83.33 | 83.33 | 100.00 | 66.67 | 100.00 | 50.00 | 85.42 |
| 0016-0032 | iBFM | 10.00 | 87.50 | 95.00 | 50.00 | 75.00 | 100.00 | 83.33 | 100.00 | 0.00 | 73.85 |
| 0016-0032 | iBFM | 12.00 | 100.00 | 100.00 | 41.67 | 75.00 | 100.00 | 66.67 | 100.00 | 33.33 | 77.08 |
| 0016-0033 | iBFM | 2.00 | 25.00 | 25.00 | 0.00 | 25.00 | 50.00 | 66.67 | 66.67 | 50.00 | 38.54 |
| 0016-0033 | iBFM | 3.00 | 50.00 | 25.00 | 0.00 | 0.00 | 50.00 | 66.67 | 66.67 | 50.00 | 38.54 |
| 0016-0033 | iBFM | 6.00 | 25.00 | 50.00 | 25.00 | 50.00 | 75.00 | 58.33 | 75.00 | 50.00 | 51.04 |
| 0016-0033 | iBFM | 8.00 | 62.50 | 45.00 | 0.00 | 41.67 | 58.33 | 58.33 | 58.33 | 50.00 | 46.77 |
| 0016-0033 | iBFM | 9.00 | 50.00 | 50.00 | 41.67 | 41.67 | 75.00 | 41.67 | 66.67 | 50.00 | 52.08 |
| 0016-0033 | iBFM | 10.00 | 75.00 | 80.00 | 50.00 | 75.00 | 75.00 | 75.00 | 66.67 | 58.33 | 69.38 |
| 0016-0033 | iBFM | 11.00 | 50.00 | 55.00 | 0.00 | 58.33 | 75.00 | 75.00 | 75.00 | 66.67 | 56.88 |
| 0016-0033 | iBFM | 12.00 | 62.50 | 65.00 | 50.00 | 50.00 | 66.67 | 75.00 | 66.67 | 58.33 | 61.77 |
| 0016-0034 | iBFM | 2.00 | 37.50 | 45.00 | 0.00 | 41.67 | 999.00 | 100.00 | 100.00 | 100.00 | 60.60 |
| 0016-0034 | iBFM | 6.00 | 100.00 | 55.00 | 58.33 | 100.00 | 66.67 | 100.00 | 100.00 | 75.00 | 81.88 |
| 0016-0034 | iBFM | 7.00 | 37.50 | 75.00 | 0.00 | 75.00 | 25.00 | 100.00 | 58.33 | 100.00 | 58.85 |
| 0016-0034 | iBFM | 9.00 | 75.00 | 30.00 | 58.33 | 75.00 | 100.00 | 100.00 | 100.00 | 100.00 | 79.79 |
| 0016-0035 | iBFM | 1.00 | 25.00 | 50.00 | 0.00 | 0.00 | 100.00 | 41.67 | 100.00 | 83.33 | 50.00 |
| 0016-0035 | iBFM | 3.00 | 25.00 | 20.00 | 0.00 | 8.33 | 75.00 | 66.67 | 100.00 | 41.67 | 42.08 |
| 0016-0037 | iBFM | 1.00 | 75.00 | 70.00 | 33.33 | 66.67 | 75.00 | 75.00 | 66.67 | 66.67 | 66.04 |
| 0016-0038 | iBFM | 1.00 | 50.00 | 65.00 | 75.00 | 50.00 | 50.00 | 50.00 | 25.00 | 75.00 | 55.00 |
| 0016-0043 | iBFM | 2.00 | 50.00 | 45.00 | 16.67 | 83.33 | 75.00 | 41.67 | 58.33 | 0.00 | 46.25 |
| 0016-0043 | iBFM | 6.00 | 50.00 | 60.00 | 58.33 | 66.67 | 66.67 | 41.67 | 75.00 | 75.00 | 61.67 |
| 0016-0043 | iBFM | 7.00 | 62.50 | 65.00 | 50.00 | 66.67 | 58.33 | 50.00 | 66.67 | 91.67 | 63.85 |
| 0016-0043 | iBFM | 8.00 | 75.00 | 55.00 | 50.00 | 66.67 | 75.00 | 50.00 | 75.00 | 33.33 | 60.00 |

Note: 999.00=missing data

Data Table 2. PedsQL Cancer Data

| **ID** | **Protocol** | **Months**  **Post-Diagnosis** | **PedsQL Pain** | **PedsQL Nausea** | **PedsQL Procedural Anxiety** | **PedsQL Treatment Anxiety** | **PedsQL Worry** | **PedsQL Cognitive Function** | **PedsQL Physical Appearance** | **PedsQL Communication** | **PedsQL Total** |
| --- | --- | --- | --- | --- | --- | --- | --- | --- | --- | --- | --- |
| 0016-0043 | iBFM | 9.00 | 75.00 | 55.00 | 50.00 | 66.67 | 58.33 | 50.00 | 75.00 | 75.00 | 63.13 |
| 0016-0043 | iBFM | 11.00 | 100.00 | 65.00 | 75.00 | 75.00 | 75.00 | 50.00 | 75.00 | 75.00 | 73.75 |
| 0016-0043 | iBFM | 12.00 | 75.00 | 65.00 | 75.00 | 100.00 | 58.33 | 50.00 | 75.00 | 75.00 | 71.67 |
| 0016-0045 | iBFM | 2.00 | 62.50 | 60.00 | 0.00 | 91.67 | 100.00 | 66.67 | 83.33 | 75.00 | 67.40 |
| 0016-0045 | iBFM | 3.00 | 62.50 | 70.00 | 25.00 | 91.67 | 100.00 | 83.33 | 91.67 | 100.00 | 78.02 |
| 0016-0045 | iBFM | 4.00 | 62.50 | 55.00 | 25.00 | 100.00 | 100.00 | 58.33 | 50.00 | 83.33 | 66.77 |
| 0016-0045 | iBFM | 5.00 | 87.50 | 60.00 | 0.00 | 75.00 | 100.00 | 58.33 | 75.00 | 75.00 | 66.35 |
| 0016-0045 | iBFM | 6.00 | 37.50 | 55.00 | 0.00 | 66.67 | 91.67 | 66.67 | 83.33 | 75.00 | 59.48 |
| 0016-0045 | iBFM | 8.00 | 37.50 | 55.00 | 8.33 | 66.67 | 75.00 | 50.00 | 83.33 | 75.00 | 56.35 |
| 0016-0045 | iBFM | 9.00 | 75.00 | 75.00 | 999.00 | 58.33 | 83.33 | 58.33 | 100.00 | 75.00 | 75.00 |
| 0016-0045 | iBFM | 10.00 | 87.50 | 60.00 | 33.33 | 91.67 | 100.00 | 66.67 | 91.67 | 100.00 | 78.85 |
| 0016-0045 | iBFM | 11.00 | 62.50 | 62.50 | 8.33 | 83.33 | 83.33 | 58.33 | 100.00 | 75.00 | 66.67 |
| 0016-0045 | iBFM | 12.00 | 75.00 | 60.00 | 8.33 | 50.00 | 91.67 | 91.67 | 100.00 | 83.33 | 70.00 |
| 0016-0046 | iBFM | 5.00 | 50.00 | 40.00 | 66.67 | 75.00 | 75.00 | 50.00 | 91.67 | 83.33 | 66.46 |
| 0016-0046 | iBFM | 6.00 | 62.50 | 65.00 | 75.00 | 75.00 | 75.00 | 41.67 | 75.00 | 75.00 | 68.02 |
| 0016-0046 | iBFM | 7.00 | 62.50 | 55.00 | 83.33 | 100.00 | 75.00 | 58.33 | 100.00 | 75.00 | 76.15 |
| 0016-0046 | iBFM | 8.00 | 62.50 | 65.00 | 66.67 | 100.00 | 100.00 | 58.33 | 100.00 | 83.33 | 79.48 |
| 0016-0046 | iBFM | 9.00 | 75.00 | 75.00 | 75.00 | 91.67 | 91.67 | 50.00 | 100.00 | 91.67 | 81.25 |
| 0016-0046 | iBFM | 10.00 | 87.50 | 95.00 | 100.00 | 100.00 | 100.00 | 83.33 | 100.00 | 100.00 | 95.73 |
| 0016-0046 | iBFM | 11.00 | 100.00 | 100.00 | 100.00 | 100.00 | 91.67 | 75.00 | 100.00 | 100.00 | 95.83 |
| 0016-0047 | iBFM | 4.00 | 100.00 | 60.00 | 0.00 | 83.33 | 100.00 | 62.50 | 100.00 | 100.00 | 75.73 |
| 0016-0047 | iBFM | 6.00 | 87.50 | 95.00 | 16.67 | 100.00 | 91.67 | 87.50 | 83.33 | 100.00 | 82.71 |
| 0016-0047 | iBFM | 7.00 | 62.50 | 70.00 | 16.67 | 91.67 | 91.67 | 81.25 | 91.67 | 83.33 | 73.59 |
| 0016-0047 | iBFM | 8.00 | 100.00 | 90.00 | 16.67 | 91.67 | 58.33 | 81.25 | 83.33 | 100.00 | 77.66 |
| 0016-0047 | iBFM | 9.00 | 62.50 | 95.00 | 8.33 | 91.67 | 100.00 | 81.25 | 100.00 | 100.00 | 79.84 |
| 0016-0047 | iBFM | 10.00 | 87.50 | 100.00 | 50.00 | 91.67 | 83.33 | 68.75 | 100.00 | 100.00 | 85.16 |
| 0016-0047 | iBFM | 11.00 | 75.00 | 100.00 | 16.67 | 83.33 | 100.00 | 75.00 | 100.00 | 100.00 | 81.25 |
| 0016-0047 | iBFM | 12.00 | 62.50 | 100.00 | 50.00 | 91.67 | 66.67 | 75.00 | 100.00 | 100.00 | 80.73 |
| 0016-0048 | iBFM | 7.00 | 100.00 | 45.00 | 33.33 | 91.67 | 100.00 | 91.67 | 100.00 | 83.33 | 80.63 |
| 0016-0048 | iBFM | 8.00 | 87.50 | 60.00 | 33.33 | 100.00 | 100.00 | 100.00 | 100.00 | 100.00 | 85.10 |
| 0016-0048 | iBFM | 9.00 | 100.00 | 65.00 | 33.33 | 100.00 | 100.00 | 91.67 | 100.00 | 91.67 | 85.21 |

Note: 999.00=missing data

Data Table 2. PedsQL Cancer Data

| **ID** | **Protocol** | **Months**  **Post-Diagnosis** | **PedsQL Pain** | **PedsQL Nausea** | **PedsQL Procedural Anxiety** | **PedsQL Treatment Anxiety** | **PedsQL Worry** | **PedsQL Cognitive Function** | **PedsQL Physical Appearance** | **PedsQL Communication** | **PedsQL Total** |
| --- | --- | --- | --- | --- | --- | --- | --- | --- | --- | --- | --- |
| 0016-0048 | iBFM | 10.00 | 100.00 | 100.00 | 100.00 | 100.00 | 100.00 | 100.00 | 100.00 | 100.00 | 100.00 |
| 0016-0048 | iBFM | 11.00 | 75.00 | 70.00 | 66.67 | 83.33 | 100.00 | 100.00 | 100.00 | 100.00 | 86.88 |
| 0016-0048 | iBFM | 12.00 | 62.50 | 45.00 | 66.67 | 83.33 | 100.00 | 100.00 | 100.00 | 100.00 | 82.19 |
| 0016-0051 | iBFM | 1.00 | 50.00 | 50.00 | 25.00 | 41.67 | 100.00 | 58.33 | 58.33 | 16.67 | 50.00 |
| 0016-0051 | iBFM | 2.00 | 75.00 | 45.00 | 50.00 | 66.67 | 100.00 | 66.67 | 100.00 | 16.67 | 65.00 |
| 0016-0051 | iBFM | 3.00 | 87.50 | 60.00 | 100.00 | 75.00 | 100.00 | 75.00 | 100.00 | 50.00 | 80.94 |
| 0016-0051 | iBFM | 5.00 | 75.00 | 50.00 | 100.00 | 58.33 | 100.00 | 50.00 | 91.67 | 33.33 | 69.79 |
| 0016-0051 | iBFM | 6.00 | 75.00 | 55.00 | 100.00 | 75.00 | 100.00 | 83.33 | 83.33 | 50.00 | 77.71 |
| 0016-0051 | iBFM | 7.00 | 50.00 | 45.00 | 75.00 | 66.67 | 75.00 | 41.67 | 75.00 | 25.00 | 56.67 |
| 0016-0051 | iBFM | 8.00 | 50.00 | 40.00 | 25.00 | 66.67 | 100.00 | 66.67 | 100.00 | 50.00 | 62.29 |
| 0016-0051 | iBFM | 9.00 | 75.00 | 65.00 | 50.00 | 58.33 | 100.00 | 66.67 | 100.00 | 75.00 | 73.75 |
| 0016-0051 | iBFM | 10.00 | 100.00 | 65.00 | 75.00 | 66.67 | 100.00 | 75.00 | 100.00 | 75.00 | 82.08 |
| 0016-0055 | iBFM | 1.00 | 0.00 | 55.00 | 58.33 | 33.33 | 100.00 | 25.00 | 75.00 | 33.33 | 47.50 |
| 0016-0056 | iBFM | 2.00 | 62.50 | 60.00 | 0.00 | 0.00 | 100.00 | 75.00 | 100.00 | 0.00 | 49.69 |
| 0016-0056 | iBFM | 6.00 | 25.00 | 30.00 | 0.00 | 0.00 | 50.00 | 41.67 | 16.67 | 0.00 | 20.42 |
| 0016-0056 | iBFM | 7.00 | 37.50 | 45.00 | 0.00 | 0.00 | 100.00 | 33.33 | 41.67 | 0.00 | 32.19 |
| 0016-0056 | iBFM | 9.00 | 37.50 | 90.00 | 0.00 | 0.00 | 100.00 | 50.00 | 100.00 | 0.00 | 47.19 |
| 0016-0056 | iBFM | 10.00 | 50.00 | 60.00 | 0.00 | 0.00 | 100.00 | 50.00 | 66.67 | 0.00 | 40.83 |
| 0016-0061 | iBFM | 3.00 | 87.50 | 70.00 | 25.00 | 91.67 | 91.67 | 83.33 | 100.00 | 100.00 | 81.15 |
| 0016-0061 | iBFM | 4.00 | 87.50 | 90.00 | 50.00 | 75.00 | 83.33 | 75.00 | 100.00 | 100.00 | 82.60 |
| 0016-0061 | iBFM | 5.00 | 75.00 | 80.00 | 75.00 | 100.00 | 100.00 | 75.00 | 100.00 | 91.67 | 87.08 |
| 0016-0061 | iBFM | 6.00 | 62.50 | 80.00 | 75.00 | 100.00 | 91.67 | 66.67 | 100.00 | 100.00 | 84.48 |
| 0016-0061 | iBFM | 7.00 | 87.50 | 80.00 | 75.00 | 100.00 | 100.00 | 83.33 | 100.00 | 100.00 | 90.73 |
| 0016-0061 | iBFM | 8.00 | 87.50 | 85.00 | 50.00 | 100.00 | 100.00 | 91.67 | 100.00 | 100.00 | 89.27 |
| 0016-0062 | iBFM | 1.00 | 37.50 | 90.00 | 41.67 | 100.00 | 100.00 | 100.00 | 100.00 | 100.00 | 83.65 |
| 0016-0062 | iBFM | 2.00 | 62.50 | 70.00 | 8.33 | 91.67 | 100.00 | 100.00 | 66.67 | 100.00 | 74.90 |
| 0016-0062 | iBFM | 3.00 | 62.50 | 85.00 | 0.00 | 91.67 | 100.00 | 100.00 | 91.67 | 100.00 | 78.85 |
| 0016-0062 | iBFM | 4.00 | 87.50 | 100.00 | 66.67 | 100.00 | 100.00 | 83.33 | 100.00 | 100.00 | 92.19 |
| 0016-0062 | iBFM | 5.00 | 50.00 | 100.00 | 58.33 | 91.67 | 100.00 | 100.00 | 91.67 | 75.00 | 83.33 |
| 0016-0062 | iBFM | 7.00 | 50.00 | 95.00 | 50.00 | 83.33 | 100.00 | 75.00 | 83.33 | 100.00 | 79.58 |
| 0016-0062 | iBFM | 12.00 | 50.00 | 100.00 | 50.00 | 100.00 | 50.00 | 66.67 | 66.67 | 100.00 | 72.92 |

Note: 999.00=missing data

Data Table 2. PedsQL Cancer Data

| **ID** | **Protocol** | **Months**  **Post-Diagnosis** | **PedsQL Pain** | **PedsQL Nausea** | **PedsQL Procedural Anxiety** | **PedsQL Treatment Anxiety** | **PedsQL Worry** | **PedsQL Cognitive Function** | **PedsQL Physical Appearance** | **PedsQL Communication** | **PedsQL Total** |
| --- | --- | --- | --- | --- | --- | --- | --- | --- | --- | --- | --- |
| 0016-0066 | iBFM | 1.00 | 62.50 | 50.00 | 33.33 | 41.67 | 75.00 | 66.67 | 999.00 | 0.00 | 47.02 |
| 0016-0066 | iBFM | 2.00 | 62.50 | 55.00 | 50.00 | 75.00 | 100.00 | 75.00 | 75.00 | 50.00 | 67.81 |
| 0017-0019 | COG | 8.00 | 37.50 | 55.00 | 16.67 | 66.67 | 83.33 | 75.00 | 66.67 | 100.00 | 62.60 |
| 0017-0019 | COG | 9.00 | 50.00 | 15.00 | 25.00 | 41.67 | 75.00 | 68.75 | 58.33 | 100.00 | 54.22 |
| 0017-0019 | COG | 10.00 | 62.50 | 37.50 | 33.33 | 75.00 | 100.00 | 68.75 | 58.33 | 91.67 | 65.89 |
| 0017-0019 | COG | 11.00 | 75.00 | 25.00 | 33.33 | 41.67 | 100.00 | 56.25 | 66.67 | 100.00 | 62.24 |
| 0017-0019 | COG | 12.00 | 50.00 | 20.00 | 37.50 | 58.33 | 100.00 | 75.00 | 58.33 | 100.00 | 62.40 |
| 0017-0023 | COG | 6.00 | 87.50 | 85.00 | 16.67 | 50.00 | 100.00 | 66.67 | 100.00 | 83.33 | 73.65 |
| 0017-0023 | COG | 8.00 | 87.50 | 70.00 | 25.00 | 50.00 | 25.00 | 50.00 | 83.33 | 66.67 | 57.19 |
| 0017-0023 | COG | 10.00 | 75.00 | 80.00 | 16.67 | 58.33 | 33.33 | 41.67 | 100.00 | 58.33 | 57.92 |
| 0017-0023 | COG | 12.00 | 75.00 | 70.00 | 25.00 | 33.33 | 41.67 | 50.00 | 100.00 | 50.00 | 55.63 |
| 0017-0024 | COG | 6.00 | 87.50 | 80.00 | 16.67 | 66.67 | 91.67 | 100.00 | 50.00 | 100.00 | 74.06 |
| 0017-0024 | COG | 7.00 | 62.50 | 45.00 | 50.00 | 100.00 | 75.00 | 100.00 | 91.67 | 100.00 | 78.02 |
| 0017-0024 | COG | 9.00 | 87.50 | 80.00 | 16.67 | 100.00 | 100.00 | 100.00 | 66.67 | 100.00 | 81.35 |
| 0017-0024 | COG | 10.00 | 87.50 | 75.00 | 33.33 | 100.00 | 91.67 | 100.00 | 66.67 | 100.00 | 81.77 |
| 0017-0024 | COG | 11.00 | 87.50 | 70.00 | 16.67 | 100.00 | 100.00 | 100.00 | 100.00 | 100.00 | 84.27 |
| 0017-0024 | COG | 12.00 | 87.50 | 80.00 | 33.33 | 100.00 | 100.00 | 100.00 | 100.00 | 100.00 | 87.60 |
| 0017-0025 | COG | 5.00 | 62.50 | 43.75 | 50.00 | 50.00 | 25.00 | 58.33 | 83.33 | 66.67 | 54.95 |
| 0017-0025 | COG | 6.00 | 50.00 | 40.00 | 50.00 | 50.00 | 100.00 | 66.67 | 75.00 | 25.00 | 57.08 |
| 0017-0025 | COG | 8.00 | 50.00 | 30.00 | 25.00 | 50.00 | 75.00 | 75.00 | 58.33 | 75.00 | 54.79 |
| 0017-0025 | COG | 9.00 | 62.50 | 60.00 | 50.00 | 50.00 | 75.00 | 66.67 | 75.00 | 50.00 | 61.15 |
| 0017-0025 | COG | 10.00 | 62.50 | 50.00 | 25.00 | 41.67 | 25.00 | 58.33 | 66.67 | 50.00 | 47.40 |
| 0017-0027 | COG | 6.00 | 50.00 | 25.00 | 50.00 | 999.00 | 50.00 | 75.00 | 50.00 | 75.00 | 53.57 |
| 0017-0027 | COG | 7.00 | 62.50 | 25.00 | 50.00 | 50.00 | 25.00 | 56.25 | 33.33 | 50.00 | 44.01 |
| 0017-0027 | COG | 8.00 | 50.00 | 25.00 | 50.00 | 50.00 | 50.00 | 75.00 | 50.00 | 50.00 | 50.00 |
| 0017-0027 | COG | 9.00 | 50.00 | 45.00 | 58.33 | 75.00 | 41.67 | 100.00 | 50.00 | 50.00 | 58.75 |
| 0017-0027 | COG | 10.00 | 50.00 | 25.00 | 25.00 | 25.00 | 25.00 | 75.00 | 50.00 | 58.33 | 41.67 |
| 0017-0027 | COG | 11.00 | 50.00 | 40.00 | 58.33 | 75.00 | 25.00 | 75.00 | 50.00 | 50.00 | 52.92 |
| 0017-0027 | COG | 12.00 | 50.00 | 25.00 | 50.00 | 50.00 | 50.00 | 75.00 | 50.00 | 50.00 | 50.00 |
| 0017-0029 | COG | 4.00 | 37.50 | 15.00 | 41.67 | 33.33 | 66.67 | 58.33 | 50.00 | 75.00 | 47.19 |

Note: 999.00=missing data

Data Table 2. PedsQL Cancer Data

| **ID** | **Protocol** | **Months**  **Post-Diagnosis** | **PedsQL Pain** | **PedsQL Nausea** | **PedsQL Procedural Anxiety** | **PedsQL Treatment Anxiety** | **PedsQL Worry** | **PedsQL Cognitive Function** | **PedsQL Physical Appearance** | **PedsQL Communication** | **PedsQL Total** |
| --- | --- | --- | --- | --- | --- | --- | --- | --- | --- | --- | --- |
| 0017-0029 | COG | 6.00 | 25.00 | 50.00 | 0.00 | 25.00 | 75.00 | 75.00 | 75.00 | 66.67 | 48.96 |
| 0017-0029 | COG | 9.00 | 50.00 | 25.00 | 16.67 | 25.00 | 100.00 | 58.33 | 66.67 | 75.00 | 52.08 |
| 0017-0030 | COG | 2.00 | 50.00 | 35.00 | 66.67 | 100.00 | 100.00 | 93.75 | 91.67 | 100.00 | 79.64 |
| 0017-0030 | COG | 4.00 | 50.00 | 70.00 | 66.67 | 100.00 | 100.00 | 100.00 | 100.00 | 100.00 | 85.83 |
| 0017-0030 | COG | 5.00 | 75.00 | 75.00 | 75.00 | 100.00 | 100.00 | 100.00 | 100.00 | 100.00 | 90.63 |
| 0017-0030 | COG | 6.00 | 62.50 | 65.00 | 75.00 | 100.00 | 100.00 | 100.00 | 100.00 | 100.00 | 87.81 |
| 0017-0030 | COG | 7.00 | 50.00 | 65.00 | 100.00 | 100.00 | 91.67 | 100.00 | 75.00 | 100.00 | 85.21 |
| 0017-0030 | COG | 8.00 | 50.00 | 70.00 | 100.00 | 100.00 | 100.00 | 100.00 | 100.00 | 100.00 | 90.00 |
| 0017-0031 | COG | 4.00 | 75.00 | 65.00 | 100.00 | 66.67 | 41.67 | 87.50 | 100.00 | 83.33 | 77.40 |
| 0017-0031 | COG | 8.00 | 50.00 | 60.00 | 100.00 | 100.00 | 8.33 | 75.00 | 100.00 | 66.67 | 70.00 |
| 0017-0031 | COG | 12.00 | 75.00 | 50.00 | 83.33 | 66.67 | 25.00 | 68.75 | 83.33 | 75.00 | 65.89 |
| 0017-0032 | COG | 1.00 | 62.50 | 70.00 | 0.00 | 25.00 | 75.00 | 66.67 | 100.00 | 0.00 | 49.90 |
| 0017-0032 | COG | 2.00 | 50.00 | 55.00 | 0.00 | 25.00 | 100.00 | 58.33 | 100.00 | 25.00 | 51.67 |
| 0017-0032 | COG | 5.00 | 62.50 | 50.00 | 0.00 | 41.67 | 75.00 | 58.33 | 100.00 | 25.00 | 51.56 |
| 0017-0034 | COG | 1.00 | 100.00 | 95.00 | 100.00 | 100.00 | 100.00 | 100.00 | 100.00 | 100.00 | 99.38 |
| 0017-0034 | COG | 2.00 | 100.00 | 95.00 | 100.00 | 100.00 | 100.00 | 100.00 | 100.00 | 100.00 | 99.38 |
| 0017-0034 | COG | 3.00 | 100.00 | 95.00 | 100.00 | 100.00 | 100.00 | 100.00 | 100.00 | 100.00 | 99.38 |
| 0017-0034 | COG | 6.00 | 100.00 | 90.00 | 100.00 | 100.00 | 100.00 | 999.00 | 999.00 | 0.00 | 81.67 |
| 0017-0034 | COG | 7.00 | 100.00 | 85.00 | 100.00 | 100.00 | 999.00 | 100.00 | 999.00 | 999.00 | 97.00 |
| 0017-0034 | COG | 8.00 | 100.00 | 95.00 | 91.67 | 100.00 | 100.00 | 100.00 | 999.00 | 999.00 | 97.78 |
| 0017-0034 | COG | 9.00 | 100.00 | 100.00 | 100.00 | 100.00 | 999.00 | 999.00 | 999.00 | 999.00 | 100.00 |
| 0017-0034 | COG | 10.00 | 100.00 | 100.00 | 75.00 | 100.00 | 999.00 | 999.00 | 999.00 | 999.00 | 93.75 |
| 0017-0034 | COG | 11.00 | 100.00 | 100.00 | 100.00 | 100.00 | 100.00 | 100.00 | 100.00 | 100.00 | 100.00 |
| 0017-0034 | COG | 12.00 | 100.00 | 100.00 | 100.00 | 100.00 | 100.00 | 100.00 | 100.00 | 100.00 | 100.00 |
| 0017-0035 | COG | 1.00 | 37.50 | 70.00 | 50.00 | 75.00 | 100.00 | 58.33 | 100.00 | 25.00 | 64.48 |
| 0017-0035 | COG | 2.00 | 50.00 | 55.00 | 8.33 | 50.00 | 75.00 | 50.00 | 100.00 | 50.00 | 54.79 |
| 0017-0035 | COG | 5.00 | 62.50 | 65.00 | 0.00 | 50.00 | 100.00 | 75.00 | 100.00 | 50.00 | 62.81 |
| 0017-0036 | COG | 1.00 | 62.50 | 70.00 | 0.00 | 50.00 | 91.67 | 58.33 | 50.00 | 58.33 | 55.10 |
| 0017-0036 | COG | 2.00 | 87.50 | 50.00 | 41.67 | 50.00 | 91.67 | 75.00 | 66.67 | 58.33 | 65.10 |
| 0017-0036 | COG | 4.00 | 75.00 | 45.00 | 16.67 | 50.00 | 100.00 | 58.33 | 58.33 | 66.67 | 58.75 |
| 0017-0036 | COG | 5.00 | 50.00 | 25.00 | 33.33 | 50.00 | 50.00 | 58.33 | 58.33 | 58.33 | 47.92 |

Note: 999.00=missing data

Data Table 2. PedsQL Cancer Data

| **ID** | **Protocol** | **Months**  **Post-Diagnosis** | **PedsQL Pain** | **PedsQL Nausea** | **PedsQL Procedural Anxiety** | **PedsQL Treatment Anxiety** | **PedsQL Worry** | **PedsQL Cognitive Function** | **PedsQL Physical Appearance** | **PedsQL Communication** | **PedsQL Total** |
| --- | --- | --- | --- | --- | --- | --- | --- | --- | --- | --- | --- |
| 0017-0036 | COG | 6.00 | 62.50 | 30.00 | 0.00 | 33.33 | 66.67 | 41.67 | 58.33 | 50.00 | 42.81 |
| 0017-0036 | COG | 7.00 | 75.00 | 35.00 | 33.33 | 66.67 | 66.67 | 58.33 | 50.00 | 75.00 | 57.50 |
| 0017-0036 | COG | 8.00 | 87.50 | 60.00 | 33.33 | 41.67 | 75.00 | 50.00 | 58.33 | 66.67 | 59.06 |
| 0017-0036 | COG | 9.00 | 37.50 | 55.00 | 25.00 | 50.00 | 66.67 | 58.33 | 41.67 | 50.00 | 48.02 |
| 0017-0036 | COG | 10.00 | 62.50 | 40.00 | 0.00 | 25.00 | 66.67 | 50.00 | 41.67 | 50.00 | 41.98 |
| 0017-0036 | COG | 11.00 | 62.50 | 35.00 | 0.00 | 33.33 | 75.00 | 50.00 | 50.00 | 58.33 | 45.52 |
| 0017-0036 | COG | 12.00 | 75.00 | 35.00 | 0.00 | 33.33 | 75.00 | 41.67 | 50.00 | 50.00 | 45.00 |
| 0017-0038 | COG | 2.00 | 37.50 | 45.00 | 0.00 | 58.33 | 100.00 | 50.00 | 66.67 | 16.67 | 46.77 |
| 0017-0038 | COG | 3.00 | 62.50 | 55.00 | 8.33 | 66.67 | 100.00 | 75.00 | 100.00 | 16.67 | 60.52 |
| 0017-0038 | COG | 4.00 | 62.50 | 45.00 | 58.33 | 75.00 | 100.00 | 66.67 | 100.00 | 33.33 | 67.60 |
| 0017-0038 | COG | 5.00 | 62.50 | 40.00 | 33.33 | 58.33 | 100.00 | 66.67 | 100.00 | 41.67 | 62.81 |
| 0017-0038 | COG | 6.00 | 62.50 | 45.00 | 41.67 | 75.00 | 100.00 | 41.67 | 100.00 | 41.67 | 63.44 |
| 0017-0038 | COG | 7.00 | 87.50 | 30.00 | 33.33 | 75.00 | 100.00 | 33.33 | 100.00 | 33.33 | 61.56 |
| 0017-0038 | COG | 9.00 | 62.50 | 55.00 | 58.33 | 66.67 | 100.00 | 41.67 | 100.00 | 41.67 | 65.73 |
| 0017-0038 | COG | 10.00 | 87.50 | 55.00 | 58.33 | 83.33 | 100.00 | 50.00 | 100.00 | 33.33 | 70.94 |
| 0017-0038 | COG | 11.00 | 87.50 | 55.00 | 66.67 | 83.33 | 100.00 | 50.00 | 100.00 | 50.00 | 74.06 |
| 0017-0038 | COG | 12.00 | 75.00 | 80.00 | 58.33 | 100.00 | 100.00 | 50.00 | 100.00 | 50.00 | 76.67 |
| 0017-0040 | COG | 2.00 | 100.00 | 80.00 | 100.00 | 100.00 | 100.00 | 100.00 | 100.00 | 50.00 | 91.25 |
| 0017-0040 | COG | 3.00 | 100.00 | 90.00 | 83.33 | 100.00 | 100.00 | 100.00 | 100.00 | 100.00 | 96.67 |
| 0017-0040 | COG | 5.00 | 75.00 | 75.00 | 83.33 | 100.00 | 83.33 | 83.33 | 100.00 | 100.00 | 87.50 |
| 0017-0040 | COG | 7.00 | 100.00 | 100.00 | 100.00 | 100.00 | 100.00 | 100.00 | 100.00 | 100.00 | 100.00 |
| 0017-0040 | COG | 8.00 | 100.00 | 100.00 | 100.00 | 100.00 | 100.00 | 100.00 | 100.00 | 100.00 | 100.00 |
| 0017-0040 | COG | 9.00 | 100.00 | 90.00 | 100.00 | 100.00 | 100.00 | 100.00 | 100.00 | 100.00 | 98.75 |
| 0017-0043 | COG | 1.00 | 62.50 | 40.00 | 0.00 | 8.33 | 100.00 | 66.67 | 100.00 | 66.67 | 55.52 |
| 0017-0043 | COG | 2.00 | 62.50 | 35.00 | 0.00 | 0.00 | 999.00 | 41.67 | 999.00 | 0.00 | 23.19 |
| 0017-0043 | COG | 3.00 | 25.00 | 35.00 | 0.00 | 0.00 | 50.00 | 50.00 | 25.00 | 25.00 | 26.25 |
| 0017-0043 | COG | 4.00 | 50.00 | 45.00 | 0.00 | 0.00 | 100.00 | 83.33 | 66.67 | 0.00 | 43.13 |
| 0017-0043 | COG | 5.00 | 37.50 | 35.00 | 0.00 | 0.00 | 75.00 | 33.33 | 58.33 | 83.33 | 40.31 |
| 0017-0043 | COG | 7.00 | 50.00 | 40.00 | 0.00 | 0.00 | 75.00 | 41.67 | 66.67 | 33.33 | 38.33 |

Note: 999.00=missing data

Data Table 2. PedsQL Cancer Data

| **ID** | **Protocol** | **Months**  **Post-Diagnosis** | **PedsQL Pain** | **PedsQL Nausea** | **PedsQL Procedural Anxiety** | **PedsQL Treatment Anxiety** | **PedsQL Worry** | **PedsQL Cognitive Function** | **PedsQL Physical Appearance** | **PedsQL Communication** | **PedsQL Total** |
| --- | --- | --- | --- | --- | --- | --- | --- | --- | --- | --- | --- |
| 0017-0043 | COG | 8.00 | 62.50 | 40.00 | 0.00 | 0.00 | 83.33 | 41.67 | 83.33 | 41.67 | 44.06 |
| 0017-0043 | COG | 9.00 | 87.50 | 55.00 | 0.00 | 0.00 | 100.00 | 75.00 | 100.00 | 25.00 | 55.31 |
| 0017-0043 | COG | 10.00 | 62.50 | 35.00 | 0.00 | 0.00 | 100.00 | 25.00 | 83.33 | 0.00 | 38.23 |
| 0017-0043 | COG | 11.00 | 62.50 | 50.00 | 0.00 | 0.00 | 75.00 | 25.00 | 66.67 | 50.00 | 41.15 |
| 0017-0045 | COG | 2.00 | 50.00 | 50.00 | 50.00 | 66.67 | 100.00 | 25.00 | 83.33 | 58.33 | 60.42 |
| 0017-0045 | COG | 4.00 | 50.00 | 50.00 | 50.00 | 41.67 | 75.00 | 50.00 | 66.67 | 75.00 | 57.29 |
| 0017-0045 | COG | 5.00 | 25.00 | 25.00 | 25.00 | 41.67 | 75.00 | 33.33 | 33.33 | 83.33 | 42.71 |
| 0017-0045 | COG | 7.00 | 50.00 | 25.00 | 0.00 | 0.00 | 83.33 | 50.00 | 50.00 | 75.00 | 41.67 |
| 0017-0045 | COG | 8.00 | 25.00 | 30.00 | 25.00 | 25.00 | 83.33 | 33.33 | 33.33 | 58.33 | 39.17 |
| 0017-0045 | COG | 10.00 | 50.00 | 5.00 | 0.00 | 0.00 | 91.67 | 33.33 | 50.00 | 58.33 | 36.04 |
| 0017-0045 | COG | 11.00 | 25.00 | 0.00 | 0.00 | 8.33 | 91.67 | 41.67 | 41.67 | 58.33 | 33.33 |
| 0017-0045 | COG | 12.00 | 25.00 | 30.00 | 0.00 | 0.00 | 100.00 | 25.00 | 100.00 | 41.67 | 40.21 |
| 0017-0047 | COG | 1.00 | 25.00 | 80.00 | 0.00 | 100.00 | 100.00 | 91.67 | 100.00 | 0.00 | 62.08 |
| 0017-0047 | COG | 2.00 | 62.50 | 70.00 | 8.33 | 0.00 | 100.00 | 25.00 | 100.00 | 0.00 | 45.73 |
| 0017-0047 | COG | 3.00 | 62.50 | 60.00 | 66.67 | 41.67 | 100.00 | 33.33 | 100.00 | 0.00 | 58.02 |
| 0017-0047 | COG | 6.00 | 50.00 | 60.00 | 100.00 | 100.00 | 100.00 | 66.67 | 100.00 | 0.00 | 72.08 |
| 0017-0047 | COG | 7.00 | 62.50 | 45.00 | 66.67 | 100.00 | 100.00 | 58.33 | 100.00 | 66.67 | 74.90 |
| 0017-0047 | COG | 8.00 | 62.50 | 90.00 | 66.67 | 100.00 | 100.00 | 75.00 | 100.00 | 50.00 | 80.52 |
| 0017-0047 | COG | 10.00 | 62.50 | 90.00 | 83.33 | 100.00 | 100.00 | 58.33 | 100.00 | 75.00 | 83.65 |
| 0017-0047 | COG | 11.00 | 75.00 | 85.00 | 100.00 | 100.00 | 100.00 | 58.33 | 100.00 | 50.00 | 83.54 |
| 0017-0048 | COG | 1.00 | 0.00 | 0.00 | 50.00 | 25.00 | 50.00 | 58.33 | 83.33 | 100.00 | 45.83 |
| 0017-0051 | COG | 1.00 | 25.00 | 55.00 | 8.33 | 25.00 | 100.00 | 33.33 | 66.67 | 33.33 | 43.33 |
| 0017-0051 | COG | 3.00 | 50.00 | 30.00 | 25.00 | 41.67 | 50.00 | 41.67 | 41.67 | 33.33 | 39.17 |
| 0017-0051 | COG | 4.00 | 62.50 | 40.00 | 33.33 | 58.33 | 83.33 | 50.00 | 91.67 | 58.33 | 59.69 |
| 0017-0051 | COG | 7.00 | 50.00 | 35.00 | 16.67 | 50.00 | 75.00 | 8.33 | 50.00 | 33.33 | 39.79 |
| 0017-0054 | COG | 1.00 | 37.50 | 35.00 | 0.00 | 0.00 | 33.33 | 25.00 | 0.00 | 50.00 | 22.60 |
| 0017-0054 | COG | 2.00 | 37.50 | 15.00 | 0.00 | 0.00 | 41.67 | 50.00 | 8.33 | 50.00 | 25.31 |
| 0017-0054 | COG | 3.00 | 50.00 | 30.00 | 8.33 | 33.33 | 66.67 | 43.75 | 25.00 | 50.00 | 38.39 |
| 0017-0054 | COG | 4.00 | 50.00 | 50.00 | 16.67 | 66.67 | 75.00 | 31.25 | 41.67 | 58.33 | 48.70 |
| 0017-0054 | COG | 5.00 | 62.50 | 45.00 | 0.00 | 66.67 | 66.67 | 25.00 | 41.67 | 50.00 | 44.69 |
| 0017-0054 | COG | 6.00 | 50.00 | 40.00 | 8.33 | 25.00 | 58.33 | 25.00 | 58.33 | 33.33 | 37.29 |

Note: 999.00=missing data

Data Table 2. PedsQL Cancer Data

| **ID** | **Protocol** | **Months**  **Post-Diagnosis** | **PedsQL Pain** | **PedsQL Nausea** | **PedsQL Procedural Anxiety** | **PedsQL Treatment Anxiety** | **PedsQL Worry** | **PedsQL Cognitive Function** | **PedsQL Physical Appearance** | **PedsQL Communication** | **PedsQL Total** |
| --- | --- | --- | --- | --- | --- | --- | --- | --- | --- | --- | --- |
| 0017-0054 | COG | 7.00 | 37.50 | 25.00 | 8.33 | 25.00 | 66.67 | 25.00 | 50.00 | 33.33 | 33.85 |
| 0017-0054 | COG | 8.00 | 37.50 | 25.00 | 16.67 | 66.67 | 75.00 | 50.00 | 50.00 | 33.33 | 44.27 |
| 0017-0054 | COG | 9.00 | 37.50 | 30.00 | 16.67 | 75.00 | 58.33 | 6.25 | 25.00 | 16.67 | 33.18 |
| 0017-0054 | COG | 10.00 | 12.50 | 10.00 | 41.67 | 50.00 | 75.00 | 0.00 | 33.33 | 8.33 | 28.85 |
| 0017-0054 | COG | 11.00 | 25.00 | 60.00 | 0.00 | 66.67 | 91.67 | 25.00 | 50.00 | 33.33 | 43.96 |
| 0017-0056 | COG | 1.00 | 50.00 | 45.00 | 0.00 | 25.00 | 100.00 | 66.67 | 100.00 | 0.00 | 48.33 |
| 0017-0056 | COG | 2.00 | 50.00 | 40.00 | 0.00 | 25.00 | 100.00 | 33.33 | 100.00 | 0.00 | 43.54 |
| 0017-0056 | COG | 3.00 | 37.50 | 40.00 | 0.00 | 83.33 | 100.00 | 58.33 | 100.00 | 0.00 | 52.40 |
| 0017-0056 | COG | 4.00 | 75.00 | 40.00 | 0.00 | 25.00 | 100.00 | 33.33 | 100.00 | 0.00 | 46.67 |
| 0017-0056 | COG | 5.00 | 62.50 | 35.00 | 0.00 | 75.00 | 100.00 | 58.33 | 100.00 | 0.00 | 53.85 |
| 0017-0056 | COG | 6.00 | 50.00 | 20.00 | 0.00 | 83.33 | 100.00 | 25.00 | 100.00 | 0.00 | 47.29 |
| 0017-0056 | COG | 7.00 | 50.00 | 35.00 | 0.00 | 25.00 | 100.00 | 25.00 | 100.00 | 0.00 | 41.88 |
| 0017-0056 | COG | 9.00 | 62.50 | 50.00 | 0.00 | 100.00 | 100.00 | 41.67 | 100.00 | 0.00 | 56.77 |
| 0017-0056 | COG | 11.00 | 62.50 | 45.00 | 0.00 | 75.00 | 100.00 | 50.00 | 100.00 | 0.00 | 54.06 |
| 0017-0056 | COG | 12.00 | 62.50 | 55.00 | 0.00 | 100.00 | 100.00 | 50.00 | 100.00 | 0.00 | 58.44 |
| 0017-0058 | COG | 1.00 | 100.00 | 100.00 | 91.67 | 100.00 | 100.00 | 100.00 | 100.00 | 91.67 | 97.92 |
| 0017-0059 | COG | 2.00 | 62.50 | 100.00 | 50.00 | 100.00 | 100.00 | 91.67 | 100.00 | 83.33 | 85.94 |
| 0017-0059 | COG | 3.00 | 87.50 | 90.00 | 0.00 | 50.00 | 100.00 | 50.00 | 100.00 | 50.00 | 65.94 |
| 0017-0059 | COG | 4.00 | 75.00 | 55.00 | 0.00 | 58.33 | 100.00 | 41.67 | 100.00 | 58.33 | 61.04 |
| 0017-0059 | COG | 5.00 | 37.50 | 45.00 | 0.00 | 50.00 | 83.33 | 25.00 | 100.00 | 75.00 | 51.98 |
| 0017-0059 | COG | 7.00 | 100.00 | 50.00 | 0.00 | 75.00 | 100.00 | 33.33 | 100.00 | 75.00 | 66.67 |
| 0017-0059 | COG | 8.00 | 62.50 | 55.00 | 16.67 | 50.00 | 100.00 | 33.33 | 100.00 | 66.67 | 60.52 |
| 0017-0059 | COG | 10.00 | 75.00 | 50.00 | 25.00 | 66.67 | 91.67 | 50.00 | 100.00 | 75.00 | 66.67 |
| 0017-0059 | COG | 11.00 | 50.00 | 35.00 | 50.00 | 75.00 | 91.67 | 66.67 | 100.00 | 75.00 | 67.92 |
| 0017-0059 | COG | 12.00 | 87.50 | 40.00 | 50.00 | 50.00 | 100.00 | 58.33 | 100.00 | 75.00 | 70.10 |
| 0017-0060 | COG | 2.00 | 62.50 | 90.00 | 0.00 | 25.00 | 100.00 | 83.33 | 100.00 | 0.00 | 57.60 |
| 0017-0060 | COG | 3.00 | 62.50 | 80.00 | 0.00 | 58.33 | 100.00 | 100.00 | 100.00 | 83.33 | 73.02 |
| 0017-0060 | COG | 7.00 | 62.50 | 65.00 | 0.00 | 0.00 | 91.67 | 66.67 | 100.00 | 75.00 | 57.60 |
| 0017-0060 | COG | 8.00 | 62.50 | 75.00 | 8.33 | 41.67 | 100.00 | 100.00 | 100.00 | 33.33 | 65.10 |
| 0017-0060 | COG | 11.00 | 62.50 | 80.00 | 0.00 | 16.67 | 100.00 | 100.00 | 100.00 | 100.00 | 69.90 |
| 0017-0063 | COG | 1.00 | 0.00 | 30.00 | 25.00 | 91.67 | 25.00 | 0.00 | 75.00 | 0.00 | 30.83 |

Note: 999.00=missing data

Data Table 2. PedsQL Cancer Data

| **ID** | **Protocol** | **Months**  **Post-Diagnosis** | **PedsQL Pain** | **PedsQL Nausea** | **PedsQL Procedural Anxiety** | **PedsQL Treatment Anxiety** | **PedsQL Worry** | **PedsQL Cognitive Function** | **PedsQL Physical Appearance** | **PedsQL Communication** | **PedsQL Total** |
| --- | --- | --- | --- | --- | --- | --- | --- | --- | --- | --- | --- |
| 0017-0063 | COG | 2.00 | 37.50 | 25.00 | 41.67 | 58.33 | 16.67 | 0.00 | 50.00 | 0.00 | 28.65 |
| 0017-0063 | COG | 3.00 | 25.00 | 25.00 | 37.50 | 75.00 | 16.67 | 18.75 | 75.00 | 0.00 | 34.11 |
| 0017-0063 | COG | 4.00 | 75.00 | 55.00 | 41.67 | 100.00 | 25.00 | 50.00 | 50.00 | 50.00 | 55.83 |
| 0017-0063 | COG | 5.00 | 62.50 | 45.00 | 50.00 | 75.00 | 25.00 | 68.75 | 75.00 | 25.00 | 53.28 |
| 0017-0063 | COG | 6.00 | 62.50 | 30.00 | 50.00 | 75.00 | 16.67 | 25.00 | 50.00 | 0.00 | 38.65 |
| 0017-0063 | COG | 8.00 | 75.00 | 75.00 | 75.00 | 75.00 | 25.00 | 18.75 | 75.00 | 25.00 | 55.47 |
| 0017-0063 | COG | 9.00 | 75.00 | 50.00 | 75.00 | 75.00 | 25.00 | 66.67 | 75.00 | 33.33 | 59.38 |
| 0017-0063 | COG | 10.00 | 75.00 | 75.00 | 58.33 | 75.00 | 50.00 | 58.33 | 75.00 | 58.33 | 65.63 |
| 0019-0001 | COG | 5.00 | 75.00 | 50.00 | 66.67 | 58.33 | 100.00 | 91.67 | 100.00 | 58.33 | 75.00 |
| 0019-0001 | COG | 7.00 | 87.50 | 50.00 | 25.00 | 75.00 | 100.00 | 91.67 | 100.00 | 66.67 | 74.48 |
| 0019-0001 | COG | 8.00 | 100.00 | 95.00 | 0.00 | 75.00 | 100.00 | 41.67 | 100.00 | 50.00 | 70.21 |
| 0019-0001 | COG | 9.00 | 87.50 | 80.00 | 50.00 | 83.33 | 100.00 | 50.00 | 100.00 | 50.00 | 75.10 |
| 0019-0001 | COG | 10.00 | 75.00 | 80.00 | 25.00 | 100.00 | 100.00 | 75.00 | 100.00 | 66.67 | 77.71 |
| 0019-0001 | COG | 11.00 | 100.00 | 100.00 | 0.00 | 100.00 | 100.00 | 50.00 | 100.00 | 50.00 | 75.00 |
| 0019-0001 | COG | 12.00 | 100.00 | 100.00 | 0.00 | 75.00 | 100.00 | 41.67 | 100.00 | 75.00 | 73.96 |
| 0019-0006 | COG | 2.00 | 37.50 | 70.00 | 0.00 | 41.67 | 100.00 | 66.67 | 66.67 | 100.00 | 60.31 |
| 0019-0006 | COG | 4.00 | 25.00 | 55.00 | 33.33 | 75.00 | 100.00 | 68.75 | 83.33 | 100.00 | 67.55 |
| 0019-0008 | COG | 2.00 | 87.50 | 75.00 | 0.00 | 41.67 | 100.00 | 41.67 | 100.00 | 100.00 | 68.23 |
| 0019-0008 | COG | 3.00 | 87.50 | 90.00 | 50.00 | 50.00 | 100.00 | 50.00 | 100.00 | 100.00 | 78.44 |
| 0019-0008 | COG | 4.00 | 62.50 | 68.75 | 25.00 | 50.00 | 100.00 | 50.00 | 100.00 | 100.00 | 69.53 |
| 0019-0008 | COG | 6.00 | 62.50 | 65.00 | 25.00 | 66.67 | 100.00 | 50.00 | 100.00 | 100.00 | 71.15 |
| 0019-0008 | COG | 7.00 | 62.50 | 55.00 | 25.00 | 50.00 | 100.00 | 50.00 | 100.00 | 100.00 | 67.81 |
| 0019-0008 | COG | 8.00 | 50.00 | 45.00 | 25.00 | 58.33 | 100.00 | 100.00 | 100.00 | 100.00 | 72.29 |
| 0019-0008 | COG | 9.00 | 37.50 | 35.00 | 25.00 | 50.00 | 100.00 | 75.00 | 100.00 | 100.00 | 65.31 |
| 0019-0008 | COG | 10.00 | 62.50 | 50.00 | 25.00 | 33.33 | 100.00 | 41.67 | 100.00 | 100.00 | 64.06 |
| 0019-0008 | COG | 11.00 | 37.50 | 30.00 | 50.00 | 50.00 | 100.00 | 25.00 | 100.00 | 100.00 | 61.56 |
| 0019-0009 | COG | 4.00 | 50.00 | 45.00 | 75.00 | 83.33 | 58.33 | 62.50 | 50.00 | 75.00 | 62.40 |
| 0019-0009 | COG | 5.00 | 87.50 | 45.00 | 58.33 | 83.33 | 58.33 | 62.50 | 25.00 | 66.67 | 60.83 |
| 0019-0009 | COG | 7.00 | 62.50 | 85.00 | 58.33 | 58.33 | 66.67 | 81.25 | 58.33 | 83.33 | 69.22 |
| 0019-0009 | COG | 8.00 | 62.50 | 50.00 | 41.67 | 66.67 | 58.33 | 50.00 | 25.00 | 75.00 | 53.65 |
| 0019-0009 | COG | 9.00 | 87.50 | 55.00 | 50.00 | 66.67 | 91.67 | 87.50 | 50.00 | 75.00 | 70.42 |

Note: 999.00=missing data

Data Table 2. PedsQL Cancer Data

| **ID** | **Protocol** | **Months**  **Post-Diagnosis** | **PedsQL Pain** | **PedsQL Nausea** | **PedsQL Procedural Anxiety** | **PedsQL Treatment Anxiety** | **PedsQL Worry** | **PedsQL Cognitive Function** | **PedsQL Physical Appearance** | **PedsQL Communication** | **PedsQL Total** |
| --- | --- | --- | --- | --- | --- | --- | --- | --- | --- | --- | --- |
| 0019-0009 | COG | 10.00 | 75.00 | 75.00 | 91.67 | 100.00 | 75.00 | 100.00 | 58.33 | 100.00 | 84.38 |
| 0019-0009 | COG | 11.00 | 62.50 | 75.00 | 100.00 | 83.33 | 100.00 | 75.00 | 50.00 | 91.67 | 79.69 |
| 0019-0009 | COG | 12.00 | 100.00 | 75.00 | 100.00 | 91.67 | 91.67 | 81.25 | 66.67 | 100.00 | 88.28 |
| 0019-0015 | COG | 3.00 | 37.50 | 15.00 | 16.67 | 50.00 | 58.33 | 62.50 | 41.67 | 100.00 | 47.71 |
| 0019-0015 | COG | 4.00 | 25.00 | 5.00 | 0.00 | 33.33 | 83.33 | 56.25 | 50.00 | 91.67 | 43.07 |
| 0019-0015 | COG | 6.00 | 37.50 | 25.00 | 25.00 | 16.67 | 75.00 | 31.25 | 66.67 | 100.00 | 47.14 |
| 0019-0015 | COG | 7.00 | 12.50 | 40.00 | 25.00 | 33.33 | 100.00 | 56.25 | 50.00 | 75.00 | 49.01 |
| 0019-0015 | COG | 8.00 | 25.00 | 10.00 | 25.00 | 25.00 | 83.33 | 56.25 | 58.33 | 75.00 | 44.74 |
| 0019-0015 | COG | 9.00 | 50.00 | 5.00 | 33.33 | 25.00 | 75.00 | 50.00 | 50.00 | 75.00 | 45.42 |
| 0019-0015 | COG | 12.00 | 62.50 | 50.00 | 50.00 | 50.00 | 100.00 | 50.00 | 58.33 | 100.00 | 65.10 |
| 0019-0017 | COG | 1.00 | 37.50 | 40.00 | 33.33 | 50.00 | 66.67 | 75.00 | 100.00 | 58.33 | 57.60 |
| 0019-0017 | COG | 3.00 | 50.00 | 50.00 | 50.00 | 58.33 | 91.67 | 91.67 | 83.33 | 100.00 | 71.88 |
| 0019-0017 | COG | 4.00 | 62.50 | 30.00 | 58.33 | 75.00 | 58.33 | 50.00 | 66.67 | 66.67 | 58.44 |
| 0019-0017 | COG | 5.00 | 50.00 | 25.00 | 58.33 | 58.33 | 58.33 | 33.33 | 66.67 | 58.33 | 51.04 |
| 0019-0017 | COG | 9.00 | 62.50 | 30.00 | 75.00 | 66.67 | 66.67 | 50.00 | 58.33 | 50.00 | 57.40 |
| 0024-0001 | COG | 8.00 | 87.50 | 70.00 | 100.00 | 100.00 | 100.00 | 87.50 | 83.33 | 100.00 | 91.04 |
| 0024-0001 | COG | 9.00 | 75.00 | 55.00 | 75.00 | 75.00 | 83.33 | 81.25 | 75.00 | 75.00 | 74.32 |
| 0024-0001 | COG | 10.00 | 75.00 | 70.00 | 100.00 | 100.00 | 100.00 | 87.50 | 100.00 | 100.00 | 91.56 |
| 0024-0001 | COG | 11.00 | 87.50 | 75.00 | 100.00 | 100.00 | 100.00 | 87.50 | 83.33 | 100.00 | 91.67 |
| 0024-0001 | COG | 12.00 | 87.50 | 90.00 | 999.00 | 100.00 | 100.00 | 87.50 | 100.00 | 100.00 | 95.00 |
| 0024-0002 | COG | 6.00 | 100.00 | 100.00 | 66.67 | 50.00 | 100.00 | 100.00 | 100.00 | 83.33 | 87.50 |
| 0024-0002 | COG | 11.00 | 100.00 | 90.00 | 50.00 | 50.00 | 100.00 | 100.00 | 100.00 | 100.00 | 86.25 |
| 0024-0002 | COG | 12.00 | 75.00 | 85.00 | 0.00 | 25.00 | 33.33 | 66.67 | 100.00 | 100.00 | 60.63 |
| 0024-0018 | COG | 1.00 | 75.00 | 55.00 | 25.00 | 25.00 | 100.00 | 75.00 | 100.00 | 100.00 | 69.38 |
| 0024-0018 | COG | 2.00 | 75.00 | 45.00 | 50.00 | 25.00 | 25.00 | 100.00 | 100.00 | 100.00 | 65.00 |
| 0024-0018 | COG | 3.00 | 75.00 | 65.00 | 41.67 | 50.00 | 100.00 | 83.33 | 100.00 | 100.00 | 76.88 |
| 0024-0018 | COG | 4.00 | 62.50 | 65.00 | 58.33 | 41.67 | 100.00 | 100.00 | 66.67 | 91.67 | 73.23 |
| 0024-0018 | COG | 5.00 | 62.50 | 65.00 | 16.67 | 25.00 | 100.00 | 75.00 | 83.33 | 66.67 | 61.77 |
| 0024-0018 | COG | 7.00 | 50.00 | 85.00 | 8.33 | 16.67 | 100.00 | 83.33 | 66.67 | 83.33 | 61.67 |
| 0024-0018 | COG | 9.00 | 62.50 | 55.00 | 25.00 | 25.00 | 100.00 | 66.67 | 58.33 | 66.67 | 57.40 |

Note: 999.00=missing data

Data Table 2. PedsQL Cancer Data

| **ID** | **Protocol** | **Months**  **Post-Diagnosis** | **PedsQL Pain** | **PedsQL Nausea** | **PedsQL Procedural Anxiety** | **PedsQL Treatment Anxiety** | **PedsQL Worry** | **PedsQL Cognitive Function** | **PedsQL Physical Appearance** | **PedsQL Communication** | **PedsQL Total** |
| --- | --- | --- | --- | --- | --- | --- | --- | --- | --- | --- | --- |
| 0024-0018 | COG | 10.00 | 75.00 | 55.00 | 25.00 | 25.00 | 100.00 | 83.33 | 66.67 | 75.00 | 63.13 |
| 0024-0018 | COG | 11.00 | 100.00 | 80.00 | 8.33 | 25.00 | 100.00 | 83.33 | 66.67 | 91.67 | 69.38 |
| 0024-0018 | COG | 12.00 | 50.00 | 55.00 | 16.67 | 41.67 | 100.00 | 58.33 | 83.33 | 75.00 | 60.00 |
| 0024-0019 | COG | 2.00 | 62.50 | 50.00 | 33.33 | 50.00 | 58.33 | 50.00 | 50.00 | 66.67 | 52.60 |
| 0024-0019 | COG | 4.00 | 100.00 | 70.00 | 33.33 | 83.33 | 83.33 | 81.25 | 75.00 | 33.33 | 69.95 |
| 0024-0019 | COG | 5.00 | 100.00 | 85.00 | 58.33 | 100.00 | 100.00 | 62.50 | 100.00 | 100.00 | 88.23 |
| 0024-0019 | COG | 6.00 | 50.00 | 70.00 | 66.67 | 100.00 | 100.00 | 100.00 | 58.33 | 100.00 | 80.63 |
| 0024-0019 | COG | 7.00 | 100.00 | 90.00 | 83.33 | 100.00 | 100.00 | 93.75 | 75.00 | 100.00 | 92.76 |
| 0024-0019 | COG | 8.00 | 100.00 | 100.00 | 91.67 | 100.00 | 100.00 | 100.00 | 100.00 | 100.00 | 98.96 |
| 0024-0019 | COG | 10.00 | 100.00 | 100.00 | 83.33 | 100.00 | 100.00 | 100.00 | 100.00 | 100.00 | 97.92 |
| 0024-0019 | COG | 11.00 | 100.00 | 100.00 | 100.00 | 100.00 | 100.00 | 100.00 | 100.00 | 100.00 | 100.00 |
| 0024-0020 | COG | 1.00 | 37.50 | 20.00 | 66.67 | 75.00 | 75.00 | 37.50 | 66.67 | 25.00 | 50.42 |
| 0024-0020 | COG | 3.00 | 37.50 | 30.00 | 83.33 | 83.33 | 83.33 | 43.75 | 100.00 | 33.33 | 61.82 |
| 0024-0023 | COG | 2.00 | 62.50 | 90.00 | 33.33 | 75.00 | 75.00 | 58.33 | 91.67 | 100.00 | 73.23 |
| 0024-0023 | COG | 4.00 | 50.00 | 60.00 | 66.67 | 91.67 | 91.67 | 100.00 | 83.33 | 75.00 | 77.29 |
| 0024-0023 | COG | 8.00 | 62.50 | 20.00 | 25.00 | 58.33 | 83.33 | 50.00 | 100.00 | 100.00 | 62.40 |
| 0067-0002 | COG | 9.00 | 0.00 | 0.00 | 16.67 | 25.00 | 25.00 | 37.50 | 25.00 | 0.00 | 16.15 |
| 0067-0002 | COG | 10.00 | 75.00 | 40.00 | 50.00 | 50.00 | 50.00 | 75.00 | 75.00 | 50.00 | 58.13 |
| 0067-0002 | COG | 11.00 | 100.00 | 45.00 | 75.00 | 75.00 | 75.00 | 75.00 | 50.00 | 75.00 | 71.25 |
| 0067-0002 | COG | 12.00 | 100.00 | 90.00 | 100.00 | 100.00 | 100.00 | 100.00 | 100.00 | 100.00 | 98.75 |
| 0067-0004 | COG | 3.00 | 50.00 | 85.00 | 25.00 | 58.33 | 91.67 | 66.67 | 50.00 | 50.00 | 59.58 |
| 0067-0004 | COG | 4.00 | 50.00 | 50.00 | 50.00 | 50.00 | 100.00 | 66.67 | 50.00 | 0.00 | 52.08 |
| 0067-0004 | COG | 5.00 | 50.00 | 60.00 | 50.00 | 66.67 | 83.33 | 75.00 | 50.00 | 25.00 | 57.50 |
| 0067-0004 | COG | 6.00 | 50.00 | 55.00 | 0.00 | 41.67 | 100.00 | 50.00 | 75.00 | 50.00 | 52.71 |
| 0067-0004 | COG | 7.00 | 25.00 | 30.00 | 0.00 | 33.33 | 66.67 | 50.00 | 33.33 | 0.00 | 29.79 |
| 0067-0004 | COG | 8.00 | 62.50 | 55.00 | 50.00 | 66.67 | 100.00 | 50.00 | 50.00 | 41.67 | 59.48 |
| 0067-0004 | COG | 10.00 | 62.50 | 60.00 | 50.00 | 75.00 | 100.00 | 50.00 | 83.33 | 50.00 | 66.35 |
| 0067-0004 | COG | 11.00 | 50.00 | 25.00 | 25.00 | 75.00 | 91.67 | 50.00 | 83.33 | 50.00 | 56.25 |
| 0067-0004 | COG | 12.00 | 62.50 | 55.00 | 50.00 | 66.67 | 100.00 | 33.33 | 83.33 | 50.00 | 62.60 |
| 0067-0005 | COG | 3.00 | 75.00 | 55.00 | 91.67 | 91.67 | 83.33 | 83.33 | 50.00 | 100.00 | 78.75 |
| 0067-0005 | COG | 4.00 | 75.00 | 50.00 | 91.67 | 100.00 | 91.67 | 75.00 | 83.33 | 91.67 | 82.29 |

Note: 999.00=missing data

Data Table 2. PedsQL Cancer Data

| **ID** | **Protocol** | **Months**  **Post-Diagnosis** | **PedsQL Pain** | **PedsQL Nausea** | **PedsQL Procedural Anxiety** | **PedsQL Treatment Anxiety** | **PedsQL Worry** | **PedsQL Cognitive Function** | **PedsQL Physical Appearance** | **PedsQL Communication** | **PedsQL Total** |
| --- | --- | --- | --- | --- | --- | --- | --- | --- | --- | --- | --- |
| 0067-0005 | COG | 5.00 | 87.50 | 60.00 | 100.00 | 100.00 | 100.00 | 91.67 | 75.00 | 100.00 | 89.27 |
| 0067-0005 | COG | 6.00 | 75.00 | 80.00 | 100.00 | 100.00 | 91.67 | 83.33 | 75.00 | 100.00 | 88.13 |
| 0067-0005 | COG | 7.00 | 87.50 | 50.00 | 100.00 | 100.00 | 100.00 | 58.33 | 75.00 | 91.67 | 82.81 |
| 0067-0005 | COG | 8.00 | 87.50 | 60.00 | 100.00 | 100.00 | 100.00 | 75.00 | 66.67 | 91.67 | 85.10 |
| 0067-0005 | COG | 9.00 | 87.50 | 70.00 | 100.00 | 91.67 | 100.00 | 66.67 | 83.33 | 100.00 | 87.40 |
| 0067-0005 | COG | 10.00 | 62.50 | 90.00 | 100.00 | 83.33 | 83.33 | 50.00 | 50.00 | 91.67 | 76.35 |
| 0067-0005 | COG | 11.00 | 75.00 | 65.00 | 66.67 | 58.33 | 75.00 | 50.00 | 41.67 | 75.00 | 63.33 |
| 0067-0005 | COG | 12.00 | 62.50 | 80.00 | 100.00 | 91.67 | 83.33 | 37.50 | 83.33 | 100.00 | 79.79 |
| 0067-0006 | COG | 3.00 | 50.00 | 35.00 | 58.33 | 91.67 | 100.00 | 66.67 | 75.00 | 100.00 | 72.08 |
| 0067-0007 | COG | 1.00 | 75.00 | 60.00 | 50.00 | 87.50 | 75.00 | 56.25 | 66.67 | 58.33 | 66.09 |
| 0067-0007 | COG | 2.00 | 62.50 | 50.00 | 25.00 | 91.67 | 66.67 | 56.25 | 66.67 | 58.33 | 59.64 |
| 0067-0007 | COG | 3.00 | 37.50 | 55.00 | 50.00 | 75.00 | 58.33 | 56.25 | 58.33 | 66.67 | 57.14 |
| 0067-0007 | COG | 4.00 | 75.00 | 40.00 | 8.33 | 91.67 | 75.00 | 62.50 | 66.67 | 58.33 | 59.69 |
| 0067-0007 | COG | 5.00 | 75.00 | 55.00 | 50.00 | 75.00 | 66.67 | 62.50 | 75.00 | 58.33 | 64.69 |
| 0067-0007 | COG | 6.00 | 50.00 | 30.00 | 8.33 | 66.67 | 50.00 | 56.25 | 66.67 | 58.33 | 48.28 |
| 0067-0007 | COG | 7.00 | 62.50 | 55.00 | 50.00 | 75.00 | 66.67 | 62.50 | 100.00 | 50.00 | 65.21 |
| 0067-0007 | COG | 8.00 | 62.50 | 55.00 | 58.33 | 75.00 | 75.00 | 62.50 | 75.00 | 75.00 | 67.29 |
| 0067-0007 | COG | 9.00 | 62.50 | 55.00 | 58.33 | 91.67 | 75.00 | 62.50 | 91.67 | 91.67 | 73.54 |
| 0067-0007 | COG | 10.00 | 75.00 | 70.00 | 83.33 | 91.67 | 75.00 | 75.00 | 100.00 | 91.67 | 82.71 |
| 0067-0007 | COG | 11.00 | 62.50 | 55.00 | 83.33 | 100.00 | 75.00 | 66.67 | 75.00 | 91.67 | 76.15 |
| 0067-0007 | COG | 12.00 | 75.00 | 60.00 | 83.33 | 100.00 | 75.00 | 68.75 | 91.67 | 100.00 | 81.72 |
| 0067-0008 | COG | 5.00 | 62.50 | 20.00 | 16.67 | 50.00 | 100.00 | 58.33 | 100.00 | 50.00 | 57.19 |
| 0067-0008 | COG | 6.00 | 50.00 | 40.00 | 0.00 | 50.00 | 100.00 | 50.00 | 100.00 | 58.33 | 56.04 |
| 0067-0008 | COG | 7.00 | 62.50 | 20.00 | 0.00 | 75.00 | 100.00 | 75.00 | 100.00 | 83.33 | 64.48 |
| 0067-0008 | COG | 8.00 | 75.00 | 40.00 | #NULL! | 75.00 | 91.67 | 83.33 | 100.00 | 100.00 | 80.71 |
| 0067-0008 | COG | 10.00 | 62.50 | 45.00 | 0.00 | 50.00 | 100.00 | 75.00 | 100.00 | 83.33 | 64.48 |

Note: 999.00=missing data

Data Table 3. Emotion Thermometer Data

| **ID** | **Protocol** | **Months**  **Post-Diagnosis** | **Parent**  **Distress** | **Parent**  **Anxiety** | **Parent**  **Depression** | **Parent**  **Anger** | **Parent**  **Need Help** |
| --- | --- | --- | --- | --- | --- | --- | --- |
| 0013-0001 | iBFM | 7.00 | 4 | 2 | 2 | 3 | 0 |
| 0013-0001 | iBFM | 8.00 | 2 | 4 | 2 | 2 | 0 |
| 0013-0001 | iBFM | 9.00 | 3 | 2 | 2 | 2 | 0 |
| 0013-0001 | iBFM | 10.00 | 1 | 2 | 2 | 2 | 0 |
| 0013-0001 | iBFM | 11.00 | 3 | 3 | 2 | 1 | 0 |
| 0013-0001 | iBFM | 12.00 | 2 | 2 | 1 | 2 | 0 |
| 0013-0003 | iBFM | 4.00 | 0 | 7 | 0 | 0 | 0 |
| 0013-0003 | iBFM | 5.00 | 8 | 7 | 0 | 0 | 0 |
| 0013-0003 | iBFM | 6.00 | 9 | 10 | 0 | 0 | 0 |
| 0013-0003 | iBFM | 7.00 | 0 | 6 | 0 | 0 | 0 |
| 0013-0003 | iBFM | 8.00 | 3 | 8 | 0 | 0 | 0 |
| 0013-0003 | iBFM | 9.00 | 0 | 0 | 0 | 0 | 0 |
| 0013-0003 | iBFM | 10.00 | 0 | 5 | 0 | 2 | 0 |
| 0013-0003 | iBFM | 11.00 | 4 | 4 | 0 | 0 | 0 |
| 0013-0003 | iBFM | 12.00 | 4 | 8 | 2 | 2 | 0 |
| 0013-0004 | iBFM | 4.00 | 2 | 3 | 2 | 3 | 0 |
| 0013-0004 | iBFM | 5.00 | 1 | 2 | 2 | 2 | 0 |
| 0013-0004 | iBFM | 6.00 | 6 | 7 | 7 | 7 | 0 |
| 0013-0004 | iBFM | 7.00 | 3 | 3 | 3 | 3 | 0 |
| 0013-0004 | iBFM | 8.00 | 0 | 0 | 3 | 1 | 0 |
| 0013-0004 | iBFM | 10.00 | 0 | 1 | 0 | 0 | 0 |
| 0013-0004 | iBFM | 11.00 | 3 | 3 | 2 | 2 | 0 |
| 0013-0004 | iBFM | 12.00 | 3 | 3 | 3 | 1 | 0 |
| 0013-0005 | iBFM | 3.00 | 5 | 5 | 7 | 2 | 0 |
| 0013-0005 | iBFM | 5.00 | 7 | 8 | 8 | 2 | 0 |
| 0013-0005 | iBFM | 6.00 | 8 | 8 | 9 | 4 | 0 |
| 0013-0005 | iBFM | 7.00 | 3 | 5 | 5 | 1 | 0 |
| 0013-0005 | iBFM | 9.00 | 4 | 4 | 4 | 0 | 0 |
| 0013-0005 | iBFM | 10.00 | 0 | 2 | 2 | 0 | 0 |
| 0013-0006 | iBFM | 4.00 | 2 | 2 | 2 | 2 | 0 |
| 0013-0006 | iBFM | 5.00 | 8 | 9 | 7 | 7 | 1 |

Note: 999.00=missing data

Data Table 3. Emotion Thermometer Data

| **ID** | **Protocol** | **Months**  **Post-Diagnosis** | **Parent**  **Distress** | **Parent**  **Anxiety** | **Parent**  **Depression** | **Parent**  **Anger** | **Parent**  **Need Help** |
| --- | --- | --- | --- | --- | --- | --- | --- |
| 0013-0006 | iBFM | 7.00 | 8 | 8 | 8 | 5 | 0 |
| 0013-0006 | iBFM | 8.00 | 8 | 8 | 8 | 7 | 0 |
| 0013-0006 | iBFM | 9.00 | 6 | 6 | 6 | 6 | 0 |
| 0013-0006 | iBFM | 11.00 | 6 | 8 | 6 | 7 | 0 |
| 0013-0006 | iBFM | 12.00 | 8 | 8 | 5 | 7 | 0 |
| 0013-0008 | iBFM | 5.00 | 1 | 3 | 0 | 0 | 0 |
| 0013-0008 | iBFM | 6.00 | 0 | 3 | 6 | 6 | 0 |
| 0013-0008 | iBFM | 7.00 | 2 | 2 | 1 | 6 | 0 |
| 0013-0008 | iBFM | 8.00 | 0 | 0 | 0 | 0 | 0 |
| 0013-0008 | iBFM | 9.00 | 0 | 5 | 0 | 2 | 0 |
| 0013-0008 | iBFM | 10.00 | 0 | 0 | 5 | 4 | 0 |
| 0013-0008 | iBFM | 11.00 | 0 | 0 | 0 | 0 | 0 |
| 0014-0020 | COG | 3.00 | 2 | 6 | 2 | 7 | 4 |
| 0014-0020 | COG | 4.00 | 6 | 4 | 3 | 1 | 3 |
| 0014-0020 | COG | 5.00 | 5 | 5 | 1 | 2 | 3 |
| 0014-0020 | COG | 6.00 | 5 | 3 | 1 | 2 | 4 |
| 0014-0024 | COG | 4.00 | 8 | 8 | 3 | 3 | 0 |
| 0014-0024 | COG | 5.00 | 8 | 7 | 3 | 3 | 0 |
| 0014-0024 | COG | 6.00 | 7 | 7 | 7 | 2 | 0 |
| 0014-0024 | COG | 7.00 | 8 | 6 | 6 | 4 | 0 |
| 0014-0024 | COG | 8.00 | 7 | 5 | 4 | 3 | 0 |
| 0014-0024 | COG | 11.00 | 7 | 8 | 5 | 4 | 0 |
| 0014-0024 | COG | 12.00 | 3 | 2 | 1 | 1 | 0 |
| 0014-0025 | COG | 1.00 | 3 | 2 | 3 | 2 | 0 |
| 0014-0025 | COG | 2.00 | 4 | 3 | 2 | 7 | 0 |
| 0014-0025 | COG | 4.00 | 3 | 1 | 3 | 1 | 0 |
| 0015-0011 | COG | 5.00 | 9 | 7 | 0 | 7 | 0 |
| 0015-0011 | COG | 7.00 | 2 | 6 | 3 | 3 | 0 |

Note: 999=missing data

Data Table 3. Emotion Thermometer Data

| **ID** | **Protocol** | **Months Post-Diagnosis** | **Parent Distress** | **Parent Anxiety** | **Parent Depression** | **Parent Anger** | **Parent Need Help** |
| --- | --- | --- | --- | --- | --- | --- | --- |
| 0015-0011 | COG | 8.00 | 6 | 8 | 6 | 7 | 0 |
| 0015-0011 | COG | 9.00 | 0 | 6 | 2 | 3 | 0 |
| 0015-0012 | COG | 6.00 | 6 | 8 | 3 | 9 | 0 |
| 0015-0012 | COG | 8.00 | 3 | 4 | 3 | 5 | 0 |
| 0015-0012 | COG | 11.00 | 3 | 6 | 2 | 3 | 0 |
| 0015-0012 | COG | 12.00 | 6 | 8 | 4 | 2 | 0 |
| 0015-0018 | COG | 3.00 | 3 | 4 | 2 | 0 | 0 |
| 0015-0018 | COG | 5.00 | 8 | 8 | 8 | 8 | 0 |
| 0015-0018 | COG | 6.00 | 7 | 7 | 7 | 1 | 1 |
| 0015-0018 | COG | 7.00 | 7 | 10 | 10 | 7 | 0 |
| 0015-0018 | COG | 9.00 | 7 | 9 | 7 | 5 | 0 |
| 0015-0018 | COG | 10.00 | 10 | 10 | 6 | 8 | 0 |
| 0015-0018 | COG | 11.00 | 7 | 7 | 7 | 7 | 0 |
| 0015-0018 | COG | 12.00 | 8 | 8 | 6 | 3 | 0 |
| 0015-0019 | COG | 2.00 | 3 | 5 | 3 | 3 | 0 |
| 0015-0019 | COG | 3.00 | 2 | 4 | 3 | 3 | 0 |
| 0015-0019 | COG | 4.00 | 2 | 5 | 2 | 4 | 0 |
| 0015-0019 | COG | 5.00 | 4 | 4 | 2 | 6 | 0 |
| 0015-0019 | COG | 6.00 | 5 | 3 | 0 | 4 | 0 |
| 0015-0019 | COG | 8.00 | 4 | 4 | 0 | 4 | 0 |
| 0015-0019 | COG | 9.00 | 5 | 5 | 2 | 2 | 0 |
| 0015-0019 | COG | 10.00 | 0 | 2 | 0 | 0 | 0 |
| 0015-0019 | COG | 11.00 | 3 | 3 | 2 | 3 | 0 |
| 0015-0019 | COG | 12.00 | 2 | 2 | 0 | 0 | 0 |
| 0015-0020 | COG | 2.00 | 5 | 5 | 2 | 0 | 0 |
| 0015-0020 | COG | 3.00 | 1 | 1 | 1 | 1 | 0 |
| 0015-0020 | COG | 7.00 | 0 | 2 | 0 | 0 | 0 |
| 0015-0020 | COG | 8.00 | 0 | 0 | 0 | 0 | 0 |
| 0015-0020 | COG | 9.00 | 0 | 1 | 0 | 0 | 0 |
| 0015-0020 | COG | 11.00 | 1 | 5 | 0 | 0 | 0 |
| 0015-0021 | COG | 3.00 | 3 | 7 | 5 | 4 | 0 |
| 0015-0021 | COG | 4.00 | 2 | 3 | 2 | 1 | 0 |

Note: 999=missing data

Data Table 3. Emotion Thermometer Data

| **ID** | **Protocol** | **Months Post-Diagnosis** | **Parent Distress** | **Parent Anxiety** | **Parent Depression** | **Parent Anger** | **Parent Need Help** |
| --- | --- | --- | --- | --- | --- | --- | --- |
| 0015-0021 | COG | 5.00 | 1 | 1 | 1 | 1 | 0 |
| 0015-0021 | COG | 6.00 | 0 | 0 | 0 | 0 | 0 |
| 0015-0021 | COG | 7.00 | 0 | 0 | 0 | 0 | 0 |
| 0015-0021 | COG | 8.00 | 0 | 0 | 0 | 0 | 0 |
| 0015-0021 | COG | 5.00 | 1 | 1 | 1 | 1 | 0 |
| 0015-0022 | COG | 2.00 | 8 | 5 | 0 | 1 | 0 |
| 0015-0022 | COG | 3.00 | 4 | 4 | 1 | 3 | 0 |
| 0015-0022 | COG | 4.00 | 4 | 2 | 0 | 2 | 0 |
| 0015-0022 | COG | 6.00 | 4 | 3 | 1 | 1 | 0 |
| 0016-0005 | iBFM | 8.00 | 3 | 4 | 4 | 1 | 1 |
| 0016-0005 | iBFM | 9.00 | 3 | 4 | 3 | 2 | 2 |
| 0016-0005 | iBFM | 10.00 | 1 | 3 | 3 | 3 | 0 |
| 0016-0005 | iBFM | 11.00 | 0 | 3 | 0 | 2 | 0 |
| 0016-0005 | iBFM | 12.00 | 1 | 1 | 3 | 0 | 1 |
| 0016-0008 | iBFM | 2.00 | 8 | 9 | 6 | 7 | 7 |
| 0016-0008 | iBFM | 3.00 | 7 | 7 | 6 | 5 | 6 |
| 0016-0008 | iBFM | 4.00 | 5 | 7 | 4 | 6 | 6 |
| 0016-0008 | iBFM | 5.00 | 5 | 5 | 3 | 3 | 5 |
| 0016-0008 | iBFM | 8.00 | 6 | 5 | 4 | 3 | 2 |
| 0016-0010 | iBFM | 2.00 | 8 | 8 | 7 | 8 | 6 |
| 0016-0010 | iBFM | 4.00 | 1 | 1 | 0 | 0 | 1 |
| 0016-0010 | iBFM | 5.00 | 3 | 3 | 2 | 2 | 0 |
| 0016-0010 | iBFM | 6.00 | 7 | 7 | 3 | 3 | 4 |
| 0016-0010 | iBFM | 7.00 | 4 | 2 | 2 | 2 | 1 |
| 0016-0010 | iBFM | 8.00 | 2 | 2 | 3 | 3 | 4 |
| 0016-0010 | iBFM | 9.00 | 2 | 2 | 2 | 2 | 2 |
| 0016-0010 | iBFM | 10.00 | 3 | 3 | 3 | 5 | 5 |
| 0016-0010 | iBFM | 11.00 | 3 | 3 | 3 | 3 | 3 |
| 0016-0010 | iBFM | 12.00 | 2 | 2 | 2 | 2 | 2 |
| 0016-0026 | iBFM | 4.00 | 6 | 9 | 7 | 7 | 0 |
| 0016-0026 | iBFM | 5.00 | 8 | 8 | 0 | 4 | 0 |
| 0016-0026 | iBFM | 6.00 | 5 | 5 | 0 | 2 | 0 |

Note: 999=missing data

Data Table 3. Emotion Thermometer Data

| **ID** | **Protocol** | **Months Post-Diagnosis** | **Parent Distress** | **Parent Anxiety** | **Parent Depression** | **Parent Anger** | **Parent Need Help** |
| --- | --- | --- | --- | --- | --- | --- | --- |
| 0016-0026 | iBFM | 7.00 | 4 | 6 | 1 | 1 | 0 |
| 0016-0026 | iBFM | 8.00 | 2 | 3 | 1 | 1 | 0 |
| 0016-0026 | iBFM | 9.00 | 1 | 5 | 5 | 1 | 0 |
| 0016-0026 | iBFM | 10.00 | 6 | 8 | 3 | 5 | 0 |
| 0016-0026 | iBFM | 11.00 | 6 | 8 | 1 | 1 | 0 |
| 0016-0026 | iBFM | 12.00 | 3 | 9 | 3 | 3 | 0 |
| 0016-0032 | iBFM | 2.00 | 1 | 4 | 3 | 2 | 0 |
| 0016-0032 | iBFM | 3.00 | 2 | 5 | 2 | 2 | 0 |
| 0016-0032 | iBFM | 5.00 | 1 | 5 | 2 | 2 | 0 |
| 0016-0032 | iBFM | 6.00 | 0 | 4 | 0 | 0 | 0 |
| 0016-0032 | iBFM | 7.00 | 0 | 4 | 1 | 1 | 0 |
| 0016-0032 | iBFM | 8.00 | 0 | 2 | 0 | 0 | 0 |
| 0016-0034 | iBFM | 6.00 | 6 | 6 | 6 | 6 | 0 |
| 0016-0034 | iBFM | 7.00 | 6 | 6 | 6 | 6 | 0 |
| 0016-0034 | iBFM | 9.00 | 5 | 5 | 5 | 5 | 0 |
| 0016-0035 | iBFM | 1.00 | 9 | 7 | 0 | 5 | 0 |
| 0016-0035 | iBFM | 3.00 | 9 | 9 | 6 | 7 | 0 |
| 0016-0037 | iBFM | 1.00 | 7 | 3 | 3 | 2 | 0 |
| 0016-0038 | iBFM | 1.00 | 4 | 7 | 6 | 5 | 1 |
| 0016-0043 | iBFM | 2.00 | 2 | 3 | 0 | 0 | 1 |
| 0016-0043 | iBFM | 6.00 | 2 | 3 | 2 | 2 | 0 |
| 0016-0043 | iBFM | 7.00 | 3 | 3 | 3 | 2 | 0 |
| 0016-0043 | iBFM | 8.00 | 2 | 3 | 2 | 0 | 0 |
| 0016-0043 | iBFM | 9.00 | 1 | 1 | 1 | 1 | 0 |
| 0016-0043 | iBFM | 11.00 | 1 | 1 | 1 | 0 | 0 |
| 0016-0043 | iBFM | 12.00 | 1 | 1 | 1 | 0 | 0 |
| 0016-0045 | iBFM | 3.00 | 2 | 7 | 0 | 1 | 0 |
| 0016-0045 | iBFM | 4.00 | 8 | 9 | 6 | 7 | 0 |
| 0016-0045 | iBFM | 5.00 | 3 | 8 | 0 | 0 | 1 |
| 0016-0045 | iBFM | 6.00 | 8 | 8 | 0 | 4 | 0 |
| 0016-0045 | iBFM | 8.00 | 7 | 8 | 2 | 1 | 0 |

Note: 999=missing data

Data Table 3. Emotion Thermometer Data

| **ID** | **Protocol** | **Months Post-Diagnosis** | **Parent Distress** | **Parent Anxiety** | **Parent Depression** | **Parent Anger** | **Parent Need Help** |
| --- | --- | --- | --- | --- | --- | --- | --- |
| 0016-0045 | iBFM | 9.00 | 7 | 7 | 4 | 8 | 0 |
| 0016-0045 | iBFM | 10.00 | 5 | 7 | 3 | 3 | 0 |
| 0016-0045 | iBFM | 11.00 | 3 | 6 | 0 | 1 | 0 |
| 0016-0045 | iBFM | 12.00 | 1 | 5 | 0 | 0 | 0 |
| 0016-0046 | iBFM | 5.00 | 2 | 1 | 1 | 1 | 0 |
| 0016-0046 | iBFM | 6.00 | 1 | 1 | 1 | 1 | 0 |
| 0016-0046 | iBFM | 7.00 | 2 | 2 | 2 | 2 | 0 |
| 0016-0046 | iBFM | 8.00 | 2 | 2 | 2 | 2 | 0 |
| 0016-0046 | iBFM | 9.00 | 1 | 1 | 1 | 1 | 0 |
| 0016-0046 | iBFM | 10.00 | 0 | 0 | 0 | 0 | 0 |
| 0016-0046 | iBFM | 11.00 | 1 | 1 | 1 | 1 | 0 |
| 0016-0047 | iBFM | 4.00 | 3 | 1 | 1 | 0 | 0 |
| 0016-0047 | iBFM | 6.00 | 1 | 1 | 0 | 0 | 0 |
| 0016-0047 | iBFM | 7.00 | 8 | 8 | 0 | 0 | 0 |
| 0016-0047 | iBFM | 8.00 | 1 | 0 | 0 | 1 | 0 |
| 0016-0047 | iBFM | 9.00 | 1 | 0 | 0 | 1 | 0 |
| 0016-0047 | iBFM | 10.00 | 0 | 0 | 0 | 1 | 0 |
| 0016-0047 | iBFM | 11.00 | 1 | 2 | 1 | 0 | 0 |
| 0016-0047 | iBFM | 12.00 | 1 | 0 | 0 | 1 | 0 |
| 0016-0048 | iBFM | 7.00 | 0 | 8 | 0 | 0 | 0 |
| 0016-0048 | iBFM | 8.00 | 0 | 0 | 0 | 0 | 0 |
| 0016-0048 | iBFM | 9.00 | 0 | 5 | 0 | 2 | 0 |
| 0016-0048 | iBFM | 10.00 | 0 | 0 | 0 | 0 | 0 |
| 0016-0048 | iBFM | 11.00 | 0 | 6 | 0 | 0 | 0 |
| 0016-0048 | iBFM | 12.00 | 8 | 8 | 0 | 6 | 0 |
| 0016-0051 | iBFM | 1.00 | 5 | 7 | 6 | 7 | 0 |
| 0016-0051 | iBFM | 2.00 | 8 | 7 | 3 | 4 | 0 |
| 0016-0051 | iBFM | 3.00 | 3 | 4 | 3 | 999.00 | 0 |
| 0016-0051 | iBFM | 5.00 | 8 | 8 | 6 | 2 | 0 |
| 0016-0051 | iBFM | 6.00 | 6 | 6 | 4 | 0 | 0 |
| 0016-0051 | iBFM | 7.00 | 3 | 3 | 5 | 2 | 0 |
| 0016-0051 | iBFM | 8.00 | 5 | 5 | 5 | 2 | 0 |
| 0016-0051 | iBFM | 9.00 | 8 | 8 | 3 | 3 | 0 |

Note: 999=missing data

Data Table 3. Emotion Thermometer Data

| **ID** | **Protocol** | **Months Post-Diagnosis** | **Parent Distress** | **Parent Anxiety** | **Parent Depression** | **Parent Anger** | **Parent Need Help** |
| --- | --- | --- | --- | --- | --- | --- | --- |
| 0016-0051 | iBFM | 10.00 | 5 | 5 | 4 | 2 | 0 |
| 0016-0055 | iBFM | 1.00 | 8 | 8 | 3 | 1 | 7 |
| 0016-0056 | iBFM | 2.00 | 10 | 10 | 10 | 7 | 0 |
| 0016-0056 | iBFM | 6.00 | 10 | 10 | 10 | 10 | 0 |
| 0016-0056 | iBFM | 7.00 | 10 | 10 | 10 | 10 | 0 |
| 0016-0056 | iBFM | 9.00 | 5 | 5 | 5 | 5 | 0 |
| 0016-0056 | iBFM | 10.00 | 5 | 5 | 5 | 5 | 4 |
| 0016-0061 | iBFM | 3.00 | 3 | 6 | 1 | 0 | 0 |
| 0016-0061 | iBFM | 4.00 | 7 | 8 | 6 | 1 | 0 |
| 0016-0061 | iBFM | 5.00 | 0 | 1 | 2 | 0 | 0 |
| 0016-0061 | iBFM | 6.00 | 0 | 1 | 0 | 0 | 0 |
| 0016-0061 | iBFM | 7.00 | 0 | 2 | 1 | 0 | 0 |
| 0016-0061 | iBFM | 8.00 | 0 | 2 | 0 | 0 | 0 |
| 0016-0062 | iBFM | 1.00 | 5 | 8 | 5 | 2 | 0 |
| 0016-0062 | iBFM | 2.00 | 7 | 9 | 7 | 1 | 0 |
| 0016-0062 | iBFM | 3.00 | 9 | 9 | 6 | 4 | 0 |
| 0016-0062 | iBFM | 4.00 | 2 | 2 | 5 | 0 | 0 |
| 0016-0062 | iBFM | 5.00 | 5 | 5 | 5 | 3 | 0 |
| 0016-0062 | iBFM | 7.00 | 8 | 9 | 8 | 1 | 1 |
| 0016-0066 | iBFM | 1.00 | 9 | 8 | 7 | 3 | 0 |
| 0016-0066 | iBFM | 2.00 | 7 | 6 | 5 | 2 | 0 |
| 0017-0019 | COG | 7.00 | 3 | 7 | 0 | 5 | 0 |
| 0017-0019 | COG | 8.00 | 0 | 2 | 0 | 4 | 0 |
| 0017-0019 | COG | 9.00 | 1 | 3 | 0 | 3 | 0 |
| 0017-0019 | COG | 10.00 | 2 | 2 | 0 | 2 | 0 |
| 0017-0019 | COG | 11.00 | 1 | 1 | 0 | 1 | 0 |
| 0017-0019 | COG | 12.00 | 0 | 0 | 0 | 0 | 0 |
| 0017-0023 | COG | 6.00 | 7 | 8 | 0 | 1 | 0 |
| 0017-0023 | COG | 8.00 | 0 | 6 | 0 | 3 | 0 |
| 0017-0023 | COG | 10.00 | 0 | 0 | 0 | 4 | 0 |
| 0017-0023 | COG | 12.00 | 6 | 6 | 0 | 8 | 0 |
| 0017-0024 | COG | 6.00 | 2 | 9 | 0 | 7 | 0 |
| 0017-0024 | COG | 7.00 | 7 | 8 | 3 | 8 | 0 |
| 0017-0024 | COG | 9.00 | 0 | 0 | 0 | 0 | 0 |
| 0017-0024 | COG | 10.00 | 0 | 3 | 0 | 0 | 0 |

Note: 999=missing data

Data Table 3. Emotion Thermometer Data

| **ID** | **Protocol** | **Months Post-Diagnosis** | **Parent Distress** | **Parent Anxiety** | **Parent Depression** | **Parent Anger** | **Parent Need Help** |
| --- | --- | --- | --- | --- | --- | --- | --- |
| 0017-0024 | COG | 11.00 | 0 | 0 | 0 | 0 | 0 |
| 0017-0024 | COG | 12.00 | 0 | 0 | 0 | 0 | 0 |
| 0017-0025 | COG | 5.00 | 9 | 9 | 10 | 8 | 1 |
| 0017-0025 | COG | 6.00 | 9 | 9 | 9 | 8 | 1 |
| 0017-0025 | COG | 8.00 | 8 | 8 | 8 | 8 | 0 |
| 0017-0025 | COG | 9.00 | 6 | 6 | 6 | 6 | 0 |
| 0017-0025 | COG | 10.00 | 8 | 8 | 8 | 9 | 0 |
| 0017-0027 | COG | 6.00 | 5 | 5 | 5 | 5 | 0 |
| 0017-0027 | COG | 7.00 | 7 | 8 | 6 | 4 | 0 |
| 0017-0027 | COG | 8.00 | 5 | 5 | 5 | 5 | 0 |
| 0017-0027 | COG | 9.00 | 6 | 6 | 6 | 6 | 0 |
| 0017-0027 | COG | 10.00 | 6 | 6 | 6 | 3 | 0 |
| 0017-0027 | COG | 11.00 | 6 | 6 | 6 | 6 | 0 |
| 0017-0027 | COG | 12.00 | 6 | 6 | 6 | 1 | 0 |
| 0017-0029 | COG | 4.00 | 5 | 5 | 5 | 5 | 0 |
| 0017-0029 | COG | 6.00 | 4 | 7 | 7 | 3 | 0 |
| 0017-0029 | COG | 9.00 | 5 | 7 | 5 | 5 | 0 |
| 0017-0030 | COG | 2.00 | 4 | 4 | 4 | 3 | 0 |
| 0017-0030 | COG | 4.00 | 6 | 6 | 3 | 3 | 0 |
| 0017-0030 | COG | 5.00 | 2 | 2 | 1 | 1 | 0 |
| 0017-0030 | COG | 6.00 | 2 | 1 | 1 | 2 | 0 |
| 0017-0030 | COG | 7.00 | 1 | 3 | 1 | 1 | 0 |
| 0017-0030 | COG | 8.00 | 2 | 2 | 2 | 2 | 0 |
| 0017-0031 | COG | 4.00 | 7 | 6 | 3 | 7 | 0 |
| 0017-0031 | COG | 8.00 | 5 | 5 | 5 | 999.00 | 0 |
| 0017-0031 | COG | 12.00 | 3 | 3 | 3 | 2 | 0 |
| 0017-0032 | COG | 1.00 | 3 | 3 | 1 | 1 | 0 |
| 0017-0032 | COG | 2.00 | 8 | 8 | 2 | 7 | 0 |
| 0017-0032 | COG | 5.00 | 8 | 8 | 2 | 7 | 0 |
| 0017-0034 | COG | 1.00 | 3 | 7 | 3 | 3 | 0 |
| 0017-0034 | COG | 2.00 | 2 | 2 | 2 | 2 | 0 |
| 0017-0034 | COG | 3.00 | 0 | 0 | 0 | 0 | 0 |
| 0017-0034 | COG | 4.00 | 2 | 2 | 2 | 2 | 0 |
| 0017-0034 | COG | 6.00 | 2 | 2 | 2 | 2 | 0 |
| 0017-0034 | COG | 7.00 | 2 | 2 | 2 | 2 | 0 |
| 0017-0034 | COG | 8.00 | 2 | 2 | 2 | 2 | 0 |

Note: 999=missing data

Data Table 3. Emotion Thermometer Data

| **ID** | **Protocol** | **Months Post-Diagnosis** | **Parent Distress** | **Parent Anxiety** | **Parent Depression** | **Parent Anger** | **Parent Need Help** |
| --- | --- | --- | --- | --- | --- | --- | --- |
| 0017-0034 | COG | 9.00 | 2 | 2 | 2 | 2 | 0 |
| 0017-0034 | COG | 10.00 | 2 | 2 | 2 | 2 | 0 |
| 0017-0034 | COG | 11.00 | 1 | 1 | 1 | 1 | 0 |
| 0017-0034 | COG | 12.00 | 1 | 1 | 1 | 1 | 0 |
| 0017-0035 | COG | 1.00 | 6 | 6 | 2 | 2 | 0 |
| 0017-0035 | COG | 2.00 | 7 | 7 | 3 | 3 | 0 |
| 0017-0035 | COG | 5.00 | 4 | 4 | 4 | 4 | 0 |
| 0017-0036 | COG | 1.00 | 3 | 5 | 2 | 3 | 1 |
| 0017-0036 | COG | 2.00 | 2 | 5 | 2 | 2 | 0 |
| 0017-0036 | COG | 3.00 | 6 | 6 | 2 | 2 | 0 |
| 0017-0036 | COG | 4.00 | 7 | 8 | 6 | 3 | 0 |
| 0017-0036 | COG | 5.00 | 6 | 8 | 8 | 6 | 0 |
| 0017-0036 | COG | 6.00 | 8 | 7 | 5 | 5 | 0 |
| 0017-0036 | COG | 7.00 | 3 | 6 | 4 | 2 | 0 |
| 0017-0036 | COG | 8.00 | 9 | 9 | 7 | 6 | 0 |
| 0017-0036 | COG | 9.00 | 5 | 8 | 7 | 4 | 0 |
| 0017-0036 | COG | 10.00 | 2 | 2 | 2 | 2 | 0 |
| 0017-0036 | COG | 11.00 | 2 | 2 | 2 | 1 | 0 |
| 0017-0036 | COG | 12.00 | 3 | 6 | 3 | 2 | 0 |
| 0017-0038 | COG | 2.00 | 8 | 6 | 6 | 3 | 1 |
| 0017-0038 | COG | 3.00 | 4 | 7 | 2 | 2 | 0 |
| 0017-0038 | COG | 4.00 | 5 | 4 | 2 | 5 | 0 |
| 0017-0038 | COG | 5.00 | 3 | 6 | 5 | 7 | 0 |
| 0017-0038 | COG | 6.00 | 3 | 6 | 6 | 6 | 0 |
| 0017-0038 | COG | 7.00 | 7 | 5 | 5 | 6 | 0 |
| 0017-0038 | COG | 9.00 | 2 | 4 | 4 | 4 | 0 |
| 0017-0038 | COG | 10.00 | 2 | 2 | 2 | 2 | 0 |
| 0017-0038 | COG | 11.00 | 4 | 4 | 2 | 2 | 0 |
| 0017-0038 | COG | 12.00 | 7 | 7 | 2 | 2 | 0 |
| 0017-0040 | COG | 2.00 | 0 | 0 | 0 | 2 | 0 |
| 0017-0040 | COG | 3.00 | 0 | 0 | 0 | 0 | 0 |
| 0017-0040 | COG | 5.00 | 0 | 0 | 0 | 0 | 0 |
| 0017-0040 | COG | 7.00 | 0 | 0 | 0 | 0 | 0 |
| 0017-0040 | COG | 8.00 | 0 | 0 | 0 | 0 | 0 |
| 0017-0040 | COG | 9.00 | 0 | 0 | 0 | 1 | 0 |

Note: 999=missing data

Data Table 3. Emotion Thermometer Data

| **ID** | **Protocol** | **Months Post-Diagnosis** | **Parent Distress** | **Parent Anxiety** | **Parent Depression** | **Parent Anger** | **Parent Need Help** |
| --- | --- | --- | --- | --- | --- | --- | --- |
| 0017-0043 | COG | 1.00 | 5 | 9 | 8 | 6 | 0 |
| 0017-0043 | COG | 2.00 | 4 | 6 | 6 | 4 | 0 |
| 0017-0043 | COG | 3.00 | 8 | 9 | 8 | 8 | 0 |
| 0017-0043 | COG | 4.00 | 5 | 5 | 5 | 5 | 0 |
| 0017-0043 | COG | 5.00 | 4 | 7 | 5 | 5 | 0 |
| 0017-0043 | COG | 7.00 | 4 | 7 | 5 | 4 | 0 |
| 0017-0043 | COG | 8.00 | 6 | 7 | 5 | 4 | 0 |
| 0017-0043 | COG | 9.00 | 2 | 7 | 4 | 1 | 0 |
| 0017-0043 | COG | 10.00 | 5 | 7 | 4 | 3 | 0 |
| 0017-0043 | COG | 11.00 | 8 | 6 | 3 | 7 | 0 |
| 0017-0045 | COG | 2.00 | 2 | 10 | 2 | 2 | 1 |
| 0017-0045 | COG | 4.00 | 8 | 8 | 8 | 4 | 0 |
| 0017-0045 | COG | 5.00 | 8 | 8 | 8 | 8 | 0 |
| 0017-0045 | COG | 7.00 | 6 | 7 | 7 | 7 | 0 |
| 0017-0045 | COG | 8.00 | 10 | 10 | 10 | 10 | 0 |
| 0017-0045 | COG | 10.00 | 7 | 10 | 10 | 7 | 0 |
| 0017-0045 | COG | 11.00 | 5 | 10 | 5 | 3 | 0 |
| 0017-0045 | COG | 12.00 | 6 | 6 | 6 | 6 | 0 |
| 0017-0047 | COG | 1.00 | 7 | 8 | 0 | 8 | 0 |
| 0017-0047 | COG | 2.00 | 7 | 10 | 0 | 10 | 1 |
| 0017-0047 | COG | 3.00 | 7 | 7 | 2 | 6 | 0 |
| 0017-0047 | COG | 4.00 | 8 | 5 | 0 | 8 | 0 |
| 0017-0047 | COG | 5.00 | 8 | 0 | 0 | 10 | 0 |
| 0017-0047 | COG | 6.00 | 4 | 4 | 0 | 7 | 0 |
| 0017-0047 | COG | 7.00 | 8 | 2 | 0 | 9 | 0 |
| 0017-0047 | COG | 8.00 | 4 | 4 | 0 | 1 | 0 |
| 0017-0047 | COG | 9.00 | 3 | 2 | 0 | 5 | 0 |
| 0017-0047 | COG | 10.00 | 6 | 8 | 8 | 0 | 0 |
| 0017-0047 | COG | 11.00 | 3 | 3 | 0 | 7 | 0 |
| 0017-0047 | COG | 12.00 | 3 | 2 | 0 | 2 | 0 |
| 0017-0048 | COG | 1.00 | 10 | 9 | 2 | 2 | 0 |
| 0017-0051 | COG | 1.00 | 2 | 2 | 2 | 2 | 0 |
| 0017-0051 | COG | 3.00 | 7 | 7 | 7 | 7 | 0 |
| 0017-0051 | COG | 4.00 | 6 | 6 | 8 | 5 | 0 |
| 0017-0051 | COG | 7.00 | 6 | 8 | 8 | 6 | 0 |

Note: 999=missing data

Data Table 3. Emotion Thermometer Data

| **ID** | **Protocol** | **Months Post-Diagnosis** | **Parent Distress** | **Parent Anxiety** | **Parent Depression** | **Parent Anger** | **Parent Need Help** |
| --- | --- | --- | --- | --- | --- | --- | --- |
| 0017-0054 | COG | 1.00 | 8 | 4 | 4 | 1 | 1 |
| 0017-0054 | COG | 2.00 | 10 | 8 | 9 | 4 | 0 |
| 0017-0054 | COG | 3.00 | 10 | 10 | 7 | 2 | 0 |
| 0017-0054 | COG | 4.00 | 8 | 10 | 7 | 3 | 0 |
| 0017-0054 | COG | 5.00 | 9 | 10 | 6 | 3 | 0 |
| 0017-0054 | COG | 6.00 | 1 | 1 | 1 | 2 | 0 |
| 0017-0054 | COG | 7.00 | 1 | 1 | 1 | 2 | 0 |
| 0017-0054 | COG | 8.00 | 6 | 5 | 7 | 7 | 0 |
| 0017-0054 | COG | 9.00 | 5 | 3 | 7 | 4 | 0 |
| 0017-0054 | COG | 10.00 | 4 | 3 | 7 | 8 | 0 |
| 0017-0054 | COG | 11.00 | 7 | 3 | 8 | 8 | 1 |
| 0017-0056 | COG | 1.00 | 6 | 9 | 7 | 9 | 0 |
| 0017-0056 | COG | 2.00 | 8 | 8 | 5 | 8 | 0 |
| 0017-0056 | COG | 3.00 | 9 | 9 | 9 | 2 | 0 |
| 0017-0056 | COG | 4.00 | 6 | 6 | 3 | 3 | 0 |
| 0017-0056 | COG | 5.00 | 8 | 8 | 5 | 7 | 0 |
| 0017-0056 | COG | 6.00 | 10 | 10 | 8 | 8 | 0 |
| 0017-0056 | COG | 7.00 | 6 | 6 | 8 | 4 | 0 |
| 0017-0056 | COG | 9.00 | 7 | 9 | 6 | 7 | 0 |
| 0017-0056 | COG | 11.00 | 5 | 8 | 4 | 5 | 0 |
| 0017-0056 | COG | 12.00 | 6 | 6 | 4 | 3 | 0 |
| 0017-0058 | COG | 1.00 | 4 | 4 | 0 | 1 | 0 |
| 0017-0059 | COG | 2.00 | 0 | 1 | 0 | 0 | 0 |
| 0017-0059 | COG | 3.00 | 2 | 4 | 4 | 4 | 0 |
| 0017-0059 | COG | 4.00 | 0 | 3 | 4 | 2 | 0 |
| 0017-0059 | COG | 5.00 | 0 | 6 | 3 | 2 | 0 |
| 0017-0059 | COG | 7.00 | 0 | 0 | 0 | 0 | 0 |
| 0017-0059 | COG | 8.00 | 0 | 3 | 1 | 0 | 0 |
| 0017-0059 | COG | 10.00 | 0 | 4 | 0 | 0 | 0 |
| 0017-0059 | COG | 11.00 | 0 | 2 | 0 | 0 | 0 |
| 0017-0059 | COG | 12.00 | 0 | 4 | 0 | 0 | 0 |
| 0017-0060 | COG | 2.00 | 0 | 4 | 1 | 2 | 0 |
| 0017-0060 | COG | 3.00 | 2 | 5 | 1 | 1 | 0 |
| 0017-0060 | COG | 7.00 | 0 | 3 | 1 | 5 | 0 |
| 0017-0060 | COG | 8.00 | 2 | 3 | 0 | 2 | 0 |
| 0017-0060 | COG | 11.00 | 0 | 4 | 0 | 5 | 0 |

Note: 999=missing data

Data Table 3. Emotion Thermometer Data

| **ID** | **Protocol** | **Months Post-Diagnosis** | **Parent Distress** | **Parent Anxiety** | **Parent Depression** | **Parent Anger** | **Parent Need Help** |
| --- | --- | --- | --- | --- | --- | --- | --- |
| 0017-0063 | COG | 1.00 | 7 | 9 | 6 | 0 | 0 |
| 0017-0063 | COG | 2.00 | 7 | 9 | 7 | 1 | 0 |
| 0017-0063 | COG | 3.00 | 5 | 5 | 5 | 0 | 0 |
| 0017-0063 | COG | 4.00 | 2 | 3 | 2 | 0 | 0 |
| 0017-0063 | COG | 5.00 | 3 | 3 | 4 | 0 | 0 |
| 0017-0063 | COG | 6.00 | 5 | 5 | 5 | 0 | 0 |
| 0017-0063 | COG | 8.00 | 4 | 3 | 3 | 0 | 0 |
| 0017-0063 | COG | 9.00 | 2 | 3 | 3 | 1 | 0 |
| 0017-0063 | COG | 10.00 | 3 | 2 | 2 | 0 | 0 |
| 0019-0001 | COG | 5.00 | 7 | 8 | 5 | 6 | 0 |
| 0019-0001 | COG | 7.00 | 5 | 8 | 3 | 0 | 0 |
| 0019-0001 | COG | 8.00 | 2 | 5 | 7 | 0 | 0 |
| 0019-0001 | COG | 9.00 | 4 | 7 | 7 | 3 | 0 |
| 0019-0001 | COG | 10.00 | 7 | 4 | 5 | 2 | 0 |
| 0019-0001 | COG | 11.00 | 7 | 7 | 7 | 4 | 0 |
| 0019-0001 | COG | 12.00 | 6 | 8 | 7 | 5 | 0 |
| 0019-0001 | COG | 5.00 | 7 | 8 | 5 | 6 | 0 |
| 0019-0006 | COG | 2.00 | 7 | 2 | 0 | 7 | 0 |
| 0019-0006 | COG | 4.00 | 5 | 5 | 6 | 1 | 0 |
| 0019-0008 | COG | 2.00 | 2 | 4 | 8 | 8 | 0 |
| 0019-0008 | COG | 3.00 | 4 | 7 | 6 | 6 | 0 |
| 0019-0008 | COG | 4.00 | 5 | 7 | 8 | 7 | 0 |
| 0019-0008 | COG | 5.00 | 2 | 6 | 4 | 5 | 0 |
| 0019-0008 | COG | 6.00 | 2 | 4 | 3 | 3 | 0 |
| 0019-0008 | COG | 7.00 | 5 | 2 | 6 | 7 | 0 |
| 0019-0008 | COG | 8.00 | 7 | 8 | 4 | 9 | 0 |
| 0019-0008 | COG | 9.00 | 5 | 8 | 5 | 5 | 0 |
| 0019-0008 | COG | 10.00 | 3 | 4 | 6 | 7 | 0 |
| 0019-0008 | COG | 11.00 | 3 | 3 | 6 | 6 | 0 |
| 0019-0008 | COG | 12.00 | 4 | 3 | 7 | 8 | 0 |
| 0019-0009 | COG | 4.00 | 3 | 1 | 8 | 0 | 0 |
| 0019-0009 | COG | 5.00 | 2 | 2 | 2 | 1 | 0 |
| 0019-0009 | COG | 6.00 | 1 | 0 | 1 | 0 | 0 |
| 0019-0009 | COG | 7.00 | 0 | 3 | 0 | 0 | 0 |
| 0019-0009 | COG | 8.00 | 4 | 2 | 1 | 0 | 0 |

Note: 999=missing data

Data Table 3. Emotion Thermometer Data

| **ID** | **Protocol** | **Months Post-Diagnosis** | **Parent Distress** | **Parent Anxiety** | **Parent Depression** | **Parent Anger** | **Parent Need Help** |
| --- | --- | --- | --- | --- | --- | --- | --- |
| 0019-0009 | COG | 9.00 | 0 | 0 | 0 | 0 | 0 |
| 0019-0009 | COG | 10.00 | 1 | 0 | 0 | 0 | 0 |
| 0019-0009 | COG | 11.00 | 1 | 1 | 0 | 0 | 0 |
| 0019-0009 | COG | 12.00 | 0 | 0 | 0 | 0 | 0 |
| 0019-0015 | COG | 3.00 | 5 | 5 | 1 | 4 | 0 |
| 0019-0015 | COG | 5.00 | 5 | 5 | 1 | 1 | 0 |
| 0019-0015 | COG | 6.00 | 1 | 1 | 2 | 2 | 0 |
| 0019-0015 | COG | 7.00 | 2 | 1 | 1 | 1 | 0 |
| 0019-0015 | COG | 8.00 | 8 | 8 | 3 | 3 | 0 |
| 0019-0015 | COG | 10.00 | 2 | 0 | 0 | 0 | 0 |
| 0019-0015 | COG | 11.00 | 2 | 1 | 0 | 0 | 0 |
| 0019-0015 | COG | 12.00 | 2 | 1 | 1 | 2 | 0 |
| 0019-0017 | COG | 1.00 | 4 | 6 | 4 | 5 | 0 |
| 0019-0017 | COG | 3.00 | 4 | 3 | 8 | 3 | 0 |
| 0019-0017 | COG | 4.00 | 6 | 7 | 6 | 4 | 0 |
| 0019-0017 | COG | 5.00 | 5 | 4 | 9 | 5 | 0 |
| 0019-0017 | COG | 9.00 | 7 | 6 | 7 | 8 | 0 |
| 0024-0001 | COG | 8.00 | 3 | 7 | 0 | 0 | 0 |
| 0024-0001 | COG | 9.00 | 4 | 4 | 0 | 0 | 0 |
| 0024-0001 | COG | 10.00 | 7 | 8 | 0 | 1 | 0 |
| 0024-0001 | COG | 11.00 | 1 | 1 | 0 | 0 | 0 |
| 0024-0001 | COG | 12.00 | 0 | 2 | 0 | 0 |  |
| 0024-0002 | COG | 4.00 | 0 | 0 | 0 | 0 | 0 |
| 0024-0002 | COG | 6.00 | 2 | 1 | 0 | 0 | 0 |
| 0024-0002 | COG | 8.00 | 0 | 0 | 0 | 0 | 0 |
| 0024-0002 | COG | 11.00 | 0 | 0 | 0 | 0 | 0 |
| 0024-0002 | COG | 12.00 | 0 | 0 | 0 | 0 | 0 |

Note: 999=missing data

Data Table 3. Emotion Thermometer Data

| **ID** | **Protocol** | **Months Post-Diagnosis** | **Parent Distress** | **Parent Anxiety** | **Parent Depression** | **Parent Anger** | **Parent Need Help** |
| --- | --- | --- | --- | --- | --- | --- | --- |
| 0024-0018 | COG | 1.00 | 0 | 7 | 0 | 8 | 0 |
| 0024-0018 | COG | 10.00 | 2 | 2 | 2 | 2 | 0 |
| 0024-0018 | COG | 11.00 | 0 | 0 | 0 | 0 | 0 |
| 0024-0018 | COG | 12.00 | 4 | 2 | 0 | 0 | 0 |
| 0024-0019 | COG | 2.00 | 3 | 999 | 2 | 5 | 0 |
| 0024-0019 | COG | 4.00 | 6 | 1 | 1 | 1 | 0 |
| 0024-0019 | COG | 5.00 | 2 | 0 | 2 | 0 | 0 |
| 0024-0019 | COG | 6.00 | 1 | 1 | 2 | 1 | 0 |
| 0024-0019 | COG | 7.00 | 7 | 7 | 3 | 7 | 0 |
| 0024-0019 | COG | 8.00 | 0 | 0 | 0 | 0 | 0 |
| 0024-0019 | COG | 10.00 | 0 | 0 | 2 | 0 | 0 |
| 0024-0019 | COG | 11.00 | 1 | 1 | 1 | 3 | 0 |
| 0024-0020 | COG | 1.00 | 4 | 5 | 3 | 5 | 0 |
| 0024-0020 | COG | 3.00 | 3 | 3 | 3 | 6 | 0 |
| 0024-0023 | COG | 2.00 | 5 | 5 | 5 | 5 | 0 |
| 0024-0023 | COG | 4.00 | 6 | 6 | 6 | 6 | 0 |
| 0024-0023 | COG | 8.00 | 8 | 8 | 6 | 2 | 0 |
| 0067-0002 | COG | 9.00 | 9 | 9 | 5 | 8 | 0 |
| 0067-0002 | COG | 10.00 | 2 | 2 | 5 | 5 | 0 |
| 0067-0002 | COG | 11.00 | 6 | 6 | 6 | 6 | 0 |
| 0067-0002 | COG | 12.00 | 6 | 6 | 6 | 6 | 0 |
| 0067-0004 | COG | 3.00 | 0 | 6 | 0 | 0 | 0 |
| 0067-0004 | COG | 4.00 | 1 | 5 | 5 | 3 | 0 |
| 0067-0004 | COG | 5.00 | 1 | 5 | 0 | 0 | 0 |
| 0067-0004 | COG | 6.00 | 3 | 3 | 3 | 3 | 0 |
| 0067-0004 | COG | 7.00 | 10 | 10 | 10 | 10 | 0 |
| 0067-0004 | COG | 8.00 | 1 | 1 | 1 | 4 | 0 |
| 0067-0004 | COG | 10.00 | 1 | 1 | 1 | 1 | 0 |
| 0067-0004 | COG | 12.00 | 2 | 2 | 1 | 2 | 0 |
| 0067-0005 | COG | 3.00 | 7 | 8 | 6 | 8 | 0 |
| 0067-0005 | COG | 4.00 | 4 | 5 | 1 | 5 | 0 |
| 0067-0005 | COG | 5.00 | 1 | 1 | 0 | 1 | 0 |

Note: 999=missing data

Data Table 3. Emotion Thermometer Data

| **ID** | **Protocol** | **Months Post-Diagnosis** | **Parent Distress** | **Parent Anxiety** | **Parent Depression** | **Parent Anger** | **Parent Need Help** |
| --- | --- | --- | --- | --- | --- | --- | --- |
| 0067-0005 | COG | 6.00 | 0 | 4 | 1 | 1 | 0 |
| 0067-0005 | COG | 7.00 | 3 | 3 | 2 | 4 | 0 |
| 0067-0005 | COG | 8.00 | 5 | 5 | 2 | 4 | 0 |
| 0067-0005 | COG | 9.00 | 1 | 2 | 0 | 0 | 0 |
| 0067-0005 | COG | 10.00 | 2 | 2 | 1 | 5 | 0 |
| 0067-0005 | COG | 11.00 | 5 | 6 | 2 | 6 | 0 |
| 0067-0005 | COG | 12.00 | 3 | 4 | 1 | 4 | 0 |
| 0067-0006 | COG | 3.00 | 3 | 0 | 0 | 0 | 0 |
| 0067-0007 | COG | 1.00 | 3 | 3 | 3 | 4 | 0 |
| 0067-0007 | COG | 2.00 | 6 | 4 | 3 | 6 | 0 |
| 0067-0007 | COG | 3.00 | 7 | 9 | 4 | 4 | 0 |
| 0067-0007 | COG | 4.00 | 6 | 6 | 2 | 4 | 0 |
| 0067-0007 | COG | 5.00 | 0 | 0 | 1 | 3 | 0 |
| 0067-0007 | COG | 6.00 | 4 | 5 | 2 | 3 | 0 |
| 0067-0007 | COG | 7.00 | 4 | 5 | 1 | 4 | 0 |
| 0067-0007 | COG | 9.00 | 1 | 1 | 0 | 1 | 0 |
| 0067-0007 | COG | 10.00 | 1 | 1 | 0 | 1 | 0 |
| 0067-0007 | COG | 11.00 | 2 | 2 | 0 | 2 | 0 |
| 0067-0007 | COG | 12.00 | 0 | 0 | 0 | 0 | 0 |
| 0067-0008 | COG | 2.00 | 8 | 8 | 1 | 8 | 0 |
| 0067-0008 | COG | 5.00 | 8 | 8 | 1 | 6 | 0 |
| 0067-0008 | COG | 6.00 | 3 | 3 | 0 | 3 | 0 |
| 0067-0008 | COG | 7.00 | 2 | 2 | 2 | 2 | 0 |
| 0067-0008 | COG | 8.00 | 3 | 5 | 0 | 2 | 0 |
| 0067-0008 | COG | 10.00 | 4 | 4 | 1 | 1 | 0 |

Note: 999=missing data
